# Supplementary material for: Post-traumatic stress disorder and associated factors among road traffic accident survivors in Sub-Saharan Africa: A systematic review and meta-analysis
Source: PLoS One. 2025 Feb 24;20(2):e0318714. doi: 10.1371/journal.pone.0318714 (PMC11849852; doi:10.1371/journal.pone.0318714)
Supplement: S1 Information — (DOCX) [file pone.0318714.s001.docx]

**Supporting information 1**: Lists and reasons of excluded studies on a study of post-traumatic stress disorder and associated factors among road traffic accident survivors in Sub-Saharan Africa: A systematic review and meta-analysis

| **Authors and Publication years** | | **Reasons** **of exclusion** |
| --- | --- | --- |
|  | (Dehghan-Nayeri et al. 2017) | Title and outcome |
|  | (Chossegros et al. 2011) | Title and outcome |
|  | (Wang, Tsay, and Elaine Bond 2005) | Title and outcome |
|  | (Javidi and Yadollahie 2012) | Title and outcome |
|  | (Mekonnen et al. 2022) | Title |
|  | (Bezabh et al. 2018) | Title and outcome |
|  | (Sabahi et al. 2024) | Title |
|  | (Dai et al. 2018) | Title and outcome |
|  | (Mehta and Ameratunga 2012) | Title |
|  | (Brom, Kleber, and Hofman 1993) | Study area and outcome |
|  | (Heron-Delaney et al. 2013) | Title |
|  | (SHaH, Patel, and Patel 2024) | Study area and outcome |
|  | (Koren, Arnon, and Klein 1999) | Title and abstract |
|  | (Haque et al. 2022) | Study area and outcome |
|  | (Mason and Rowlands 1997) | Title and abstract |
|  | (Stallard, Velleman, and Baldwin 1998) | Study area |
|  | (Lin et al. 2018) | Title |
|  | (Coronas et al. 2008) | Title, abstract and study area |
|  | (Udwin et al. 2000) | Title, outcome and study area |
|  | (Arora, Belsiyal, and Rawat 2021) | Study area |
|  | (Pelissier et al. 2017) | Title and outcome |
|  | (Fuglsang, Moergeli, and Schnyder 2004) | Title and abstract |
|  | (Lee and Young 2001) | Title and abstract |
|  | (Mak et al. 2010) | Title and abstract |
|  | (Ehring, Ehlers, and Glucksman 2006) | Title and outcome |
|  | (Mirza et al. 1998) | Study area |
|  | (Elklit and Brink 2004) | Title and outcome |
|  | (Guédon-Moreau et al. 2012) | Title and outcome |
|  | (Blanchard et al. 1995) | Title and outcome |
|  | (Bown et al. 2019) | Title and outcome |
|  | (Feki et al. 2024) | Study area |
|  | (Sakuma et al. 2015) | Title and outcome |
|  | (Kaseda and Levine 2020) | Title and outcome |
|  | (Paparrigopoulos et al. 2014) | Title and outcome |
|  | (Skogstad et al. 2013) | Title and outcome |
|  | (Dai, Chen, Lai, et al. 2016) | Title and outcome |
|  | (van den Heuvel et al. 2016) | Title and outcome |
|  | (Bryant and Harvey 1995a) | Title and abstract |
|  | (Salcioglu, Basoglu, and Livanou 2007) | Title and outcome |
|  | (Neria, Nandi, and Galea 2008) | Title and outcome |
|  | (Green and Lindy 1994) | Title and outcome |
|  | (Dörfel, Rabe, and Karl* 2008) | Title and outcome |
|  | (Kleim, Ehlers, and Glucksman 2007) | Title and outcome |
|  | (Green et al. 1993) | Title and abstract |
|  | (Melkam et al. 2023) | Title |
|  | (Stallard and Smith 2007) | Title and outcome |
|  | (Haque and Sultana 2023) | Study area |
|  | (Smith et al. 1999) | Title and outcome |
|  | (Flores, Carnero, and Bayer 2014) | Title and study area |
|  | (Griffiths et al. 2007) | Title and study area |
|  | (Davis and Breslau 1994) | Title and abstract |
|  | (Yehuda 2002b) | Study area and outcome |
|  | (Mayou, Bryant, and Duthie 1993) | Title and abstract |
|  | (Tang, Deng, et al. 2017) | Title and outcome |
|  | (Bal and Jensen 2007) | Study area |
|  | (Righy et al. 2019) | Title and abstract |
|  | (Khodadadi-Hassankiadeh et al. 2017) | Study area |
|  | (Segman et al. 2005) | Title and outcome |
|  | (Fan et al. 2015) | Title and study area |
|  | (Hickling and Blanchard 1992) | Study area |
|  | (Maes, Mylle, et al. 2001) | Title and abstract |
|  | (Yehuda 2009) | Title and outcome |
|  | (Dai, Chen, Tan, et al. 2016) | Title and outcome |
|  | (Kassa et al. 2024) | Title and outcome |
|  | (Zatzick et al. 2007) | Title and abstract |
|  | (Kar 2011) | Title and outcome |
|  | (Jackson et al. 2007) | Title and outcome |
|  | (Galea, Nandi, and Vlahov 2005) | Title and outcome |
|  | (Bromet et al. 2017) | Title and outcome |
|  | (Ali et al. 2022) | Title and outcome |
|  | (Dunmore, Clark, and Ehlers 1997) | Title and outcome |
|  | (Matthews 2005) | Title and outcome |
|  | (Kilpatrick et al. 1989) | Title and outcome |
|  | (Bryant 2011) | Title and outcome |
|  | (Kun et al. 2009) | Title and outcome |
|  | (Wang et al. 2022) | Title and outcome |
|  | (Qi, Gevonden, and Shalev 2016) | Title and outcome |
|  | (Oflaz, Hatipoğlu, and Aydin 2008) | Title and outcome |
|  | (Shalev et al. 2008) | Title and abstract |
|  | (Jin, Xu, and Liu 2014) | Title and abstract |
|  | (Klein et al. 2003) | Title and abstract |
|  | (Bienvenu et al. 2013) | Title and abstract |
|  | (Johnson and Thompson 2008) | Title and outcome |
|  | (Butler, Moffic, and TURKAL 1999) | Title and abstract |
|  | (Tarsitani et al. 2021) | Title and outcome |
|  | (Goldfinger et al. 2014) | Title and outcome |
|  | (Rowan and Foy 1993) | Title and outcome |
|  | (Adhikari Baral and KC 2019) | Title and outcome |
|  | (Jaspers 1998) | Title and outcome |
|  | (Yule and Udwin 1991) | Title and abstract |
|  | (Carmassi et al. 2013) | Title and outcome |
|  | (Cuthbertson et al. 2004) | Title and outcome |
|  | (Hepp et al. 2008) | Title and outcome |
|  | (Chen et al. 2015) | Title and outcome |
|  | (Johansen, Wahl, Eilertsen, and Weisaeth 2007) | Title and outcome |
|  | (Nickerson et al. 2017) | Title and abstract |
|  | (Cha and Bang 2019) | Title and study area |
|  | (Bal 2008) | Title and outcome |
|  | (Orr, Metzger, and Pitman 2002) | Title and outcome |
|  | (Thrasher, Dalgleish, and Yule 1994) | Title and outcome |
|  | (BRYANT and HARVEY 1999a) | Title and abstract |
|  | (Ullman, Najdowski, and Filipas 2009) | Title and outcome |
|  | (Maes, Delmeire, et al. 2001) | Title and outcome |
|  | (Hu et al. 2016) | Title and outcome |
|  | (Keppel‐Benson, Ollendick, and Benson 2002) | Study area |
|  | (Vukojevic et al. 2014) | Title and outcome |
|  | (Jones, Hughes, and Unterstaller 2001) | Title and outcome |
|  | (Kar and Bastia 2006) | Title and abstract |
|  | (Mellman et al. 2001) | Study area |
|  | (Jackson et al. 2014) | Study area and outcome |
|  | (Verger et al. 2004) | Title and outcome |
|  | (Forneris et al. 2013) | Title and abstract |
|  | (Morina et al. 2014) | Title and outcome |
|  | (Hong and Efferth 2016) | Title and outcome |
|  | (Stein and Kennedy 2001) | Title and outcome |
|  | (Carr 2004) | Title and abstract |
|  | (Yule and Smith 2015) | Study area |
|  | (Foa, Zinbarg, and Rothbaum 1992) | Title and abstract |
|  | (Riggs et al. 1992) | Title and outcome |
|  | (King et al. 1998) | Title and outcome |
|  | (Kleim, Ehring, and Ehlers 2012) | Title and outcome |
|  | (Galea et al. 2003) | Title, outcome and study area |
|  | (Kolkow et al. 2007) | Title, outcome and study area |
|  | (Morina et al. 2014) | Title and abstract |
|  | (Jones, Hughes, and Unterstaller 2001) | Title and abstract |
|  | (Sutherland and Bryant 2005) | Title and abstract |
|  | (McFarlane 1988) | Title and abstract |
|  | (Stein and Kennedy 2001) | Title and abstract |
|  | (Jonsson, Segesten, and Mattsson 2003) | Title, outcome and study area |
|  | (Keppel‐Benson, Ollendick, and Benson 2002) | Study area |
|  | (Yehuda 2002a) | Title and outcome |
|  | (Kemp, Rawlings, and Green 1991) | Title and outcome |
|  | (Grunert et al. 2007) | Title and outcome |
|  | (Walters, Bisson, and Shepherd 2007) | Title and outcome |
|  | (Glynn et al. 2003) | Title and abstract |
|  | (Seedat, Stein, and Carey 2005) | Title and abstract |
|  | (Shalev, Liberzon, and Marmar 2017) | Study area |
|  | (BRYANT and HARVEY 1999a) | Title and outcome |
|  | (Kabunga et al. 2022) | Title and outcome |
|  | (Macleod 1994) | Title and outcome |
|  | (Norman et al. 2008) | Title and outcome |
|  | (Cohen et al. 2002) | Title and abstract |
|  | (Kashdan, Morina, and Priebe 2009) | Title and abstract |
|  | (Kar et al. 2007) | Title, outcome and study area |
|  | (Smith et al. 2008) | Title and outcome |
|  | (Lee, Isaac, and Janca 2002) | Title and outcome |
|  | (Maher, Rego, and Asnis 2006) | Title and outcome |
|  | (Blanchard, Hickling, Barton, et al. 1996) | Title and outcome |
|  | (Kaźmierczak, Strelau, and Zawadzki 2016) | Title and outcome |
|  | (Matsumura et al. 2017) | Title and outcome |
|  | (Kwak et al. 2013) | Title and outcome |
|  | (Gallo, Barton, and Parry-Jones 1997) | Title and outcome |
|  | (Utzon-Frank et al. 2014) | Title and abstract |
|  | (Helzer, Robins, and McEvoy 1987) | Title and outcome |
|  | (Bryant et al. 2009) | Title and outcome |
|  | (Groome and Soureti 2004) | Title and outcome |
|  | (Sloan 1988) | Title and outcome |
|  | (Girard et al. 2007) | Title and outcome |
|  | (Barton, Blanchard, and Hickling 1996) | Title and outcome |
|  | (Rose et al. 1996) | Title and outcome |
|  | (Golier and Yehuda 2002) | Title and outcome |
|  | (Hébert, Lavoie, and Blais 2014) | Title and outcome |
|  | (Chen et al. 2001) | Study area |
|  | (Descilo et al. 2010) | Title and outcome |
|  | (Landolt et al. 1998) | Title and outcome |
|  | (Patel et al. 2016) | Title and outcome |
|  | (Pillar, Malhotra, and Lavie 2000) | Title and outcome |
|  | (Turnbull 1998) | Title and abstract |
|  | (Silva 1993) | Title and outcome |
|  | (Zohar et al. 2011) | Title and outcome |
|  | (Manne, Duhamel, and Redd 2000) | Title and outcome |
|  | (Hageman, Andersen, and Jørgensen 2001) | Title and outcome |
|  | (Schreiber and Galai-Gat 1993) | Title and outcome |
|  | (Creamer and O'Donnell 2002) | Title and abstract |
|  | (Summerfield 2001) | Title and outcome |
|  | (Spoormaker and Montgomery 2008) | Title and outcome |
|  | (Fecteau and Nicki 1999) | Title and outcome |
|  | (Liriano, Hatten, and Schwartz 2019) | Title and outcome |
|  | (Khamis 2008) | Study area |
|  | (Fichera et al. 2015) | Title and outcome |
|  | (Gander and Känel 2006) | Title and outcome |
|  | (Guest et al. 2018) | Title and outcome |
|  | (Busuttil 2004) | Title and outcome |
|  | (Mitani et al. 2006) | Title and outcome |
|  | (Iversen et al. 2008) | Title, outcome and study area |
|  | (Yule 2001) | Study area |
|  | (Dyb et al. 2014) | Title, outcome and study area |
|  | (Pervanidou and Chrousos 2010) | Title and outcome |
|  | (Tanveer et al. 2023) | Study area |
|  | (Tehrani 2004) | Title and outcome |
|  | (Holbrook et al. 2010) | Title and outcome |
|  | (Blanchard, Hickling, Taylor, et al. 1996) | Title and abstract |
|  | (Nakajima et al. 2012) | Title and outcome |
|  | (Yehuda et al. 2007) | Title and outcome |
|  | (Foreman and Hough 1995) | Title and outcome |
|  | (Candel and Merckelbach 2004) | Title and outcome |
|  | (Duffy, Gillespie, and Clark 2007) | Title, outcome and study area |
|  | (Telles et al. 2010) | Title and outcome |
|  | (Kovacevic et al. 2020) | Title and abstract |
|  | (Blanc et al. 2015) | Title and outcome |
|  | (Ackerman et al. 1998) | Title and outcome |
|  | (Johansen, Wahl, Eilertsen, Weisaeth, et al. 2007) | Title and outcome |
|  | (Laffaye et al. 2007) | Title and outcome |
|  | (Maes, Delmeire, et al. 1998) | Title and outcome |
|  | (McMillan 1996) | Title and abstract |
|  | (Carlier et al. 1998) | Title and outcome |
|  | (Danielsson et al. 2018) | Title and outcome |
|  | (Perry 1994) | Title and outcome |
|  | (Hatch et al. 2018) | Title and outcome |
|  | (Vincent et al. 2008) | Title and outcome |
|  | (Mikkelsen and Einarsen 2002) | Title and outcome |
|  | (Samuelson 2011) | Title and outcome |
|  | (McNally 2006) | Title and outcome |
|  | (Ginzburg et al. 2006) | Title and outcome |
|  | (Haagsma et al. 2011) | Title and outcome |
|  | (Videlock et al. 2008) | Title and outcome |
|  | (Pynoos et al. 1993) | Title and outcome |
|  | (Naim et al. 2014) | Title and outcome |
|  | (Burstein 1986) | Title and outcome |
|  | (Jobson and O'Kearney 2008) | Title and outcome |
|  | (Landolt et al. 2009) | Title and outcome |
|  | (Miragoli, Camisasca, and Di Blasio 2017) | Title and outcome |
|  | (Boeschen et al. 2001) | Title and outcome |
|  | (Hull, Alexander, and Klein 2002) | Title and outcome |
|  | (Tay et al. 2015) | Title and outcome |
|  | (Armstrong, Shakespeare‐Finch, and Shochet 2014) | Title and outcome |
|  | (Krauss, Trankle, and Kopp 1996) | Title and outcome |
|  | (Shrestha 2015) | Title and outcome |
|  | (Meiser-Stedman et al. 2007) | Title and outcome |
|  | (Greenberg, Brooks, and Dunn 2015) | Title and outcome |
|  | (Hu et al. 2018) | Title and outcome |
|  | (Derivois et al. 2017) | Title and outcome |
|  | (Wei et al. 2017) | Title and outcome |
|  | (Pelcovitz et al. 1994) | Title and outcome |
|  | (Yehuda et al. 2015) | Study area |
|  | (Jones, Harvey, and Brewin 2007) | Title and outcome |
|  | (Shea et al. 2005) | Title and outcome |
|  | (Hughes and Shin 2011) | Title and outcome |
|  | (Abbey et al. 2015) | Title and outcome |
|  | (Lee, Shin, et al. 2019) | Title and outcome |
|  | (Housen et al. 2017) | Title and outcome |
|  | (Joseph, Williams, and Yule 1995) | Title and outcome |
|  | (Forte et al. 2020) | Title and outcome |
|  | (Cyniak-Cieciura and Zawadzki 2021) | Title and outcome |
|  | (Green, Wilson, and Lindy 2013) | Title and outcome |
|  | (Liedl et al. 2010) | Title and outcome |
|  | (Davidson et al. 1997) | Title and outcome |
|  | (Lee et al. 2009) | Title and outcome |
|  | (Joseph, Williams, and Yule 1997) | Title and outcome |
|  | (Karanci and Acarturk 2005) | Title and outcome |
|  | (Jubran et al. 2010) | Title and outcome |
|  | (LoSavio, Dillon, and Resick 2017) | Title and abstract |
|  | (Steketee and Foa 1987) | Title and abstract |
|  | (Sepahvand et al. 2019) | Outcome and study area |
|  | (Shannon et al. 1994) | Title and abstract |
|  | (Pervanidou 2008) | Title and abstract |
|  | (Ghisi et al. 2013) | Title and outcome |
|  | (Amir and Ramati 2002) | Title and outcome |
|  | (Jaapar, Abidin, and Othman 2014) | Title and outcome |
|  | (Morina et al. 2010) | Title and outcome |
|  | (Wu, Xu, and He 2014) | Title and outcome |
|  | (Sojka et al. 2006) | Title and outcome |
|  | (Haisch and Meyers 2004) | Title and outcome |
|  | (Fishbain et al. 2017) | Title and outcome |
|  | (Brousse et al. 2011) | Title and outcome |
|  | (Cantón-Cortés and Cantón 2010) | Title and outcome |
|  | (Newman, Simpson, and Handschuh 2003) | Title and outcome |
|  | (Bennett et al. 2002) | Title and outcome |
|  | (Vera and Sanz 2017) | Title and outcome |
|  | (Tomb 1994) | Title and outcome |
|  | (Livanou et al. 2005) | Title and outcome |
|  | (Koopman et al. 1995) | Title and outcome |
|  | (Kienzler 2008) | Title and outcome |
|  | (Greenspan et al. 2006) | Title and outcome |
|  | (Long et al. 2013) | Title and outcome |
|  | (Bryant 2019) | Title and outcome |
|  | (Daskalakis, Lehrner, and Yehuda 2013) | Title and outcome |
|  | (Wise and Delahanty 2017) | Study area |
|  | (Stoll et al. 2000) | Title and outcome |
|  | (O'Shea 2001) | Title and outcome |
|  | (Sherin and Nemeroff 2011) | Title and outcome |
|  | (Peterson, Prout, and Schwarz 2013) | Title and outcome |
|  | (Mojtabavi et al. 2020) | Title and outcome |
|  | (Reynolds and Brewin 1998) | Title and outcome |
|  | (Freedman et al. 1999) | Study area |
|  | (Armenian et al. 2000) | Title and outcome |
|  | (Högberg et al. 2008) | Title and outcome |
|  | (Zlotnick et al. 2006) | Title and outcome |
|  | (Dewar, Paradis, and Fortin 2020) | Title and outcome |
|  | (Dobie et al. 2002) | Title and outcome |
|  | (Grinage 2003) | Title and outcome |
|  | (Kemp, Drummond, and McDermott 2010) | Title and outcome |
|  | (Nielsen et al. 2015) | Title and outcome |
|  | (Weisæth 1989b) | Title and outcome |
|  | (Paxson et al. 2012) | Title and outcome |
|  | (Yi et al. 2022) | Title and outcome |
|  | (Farooqui et al. 2017) | Title and outcome |
|  | (Moulaert et al. 2010) | Title and outcome |
|  | (Parvaresh and Bahramnezhad 2009) | Title and outcome |
|  | (Papadakaki et al. 2017) | Title and outcome |
|  | (Rivera-Vélez et al. 2014) | Title and outcome |
|  | (Bedard-Gilligan and Zoellner 2012) | Title and outcome |
|  | (Hyman, Zelikoff, and Clarke 1988) | Title and outcome |
|  | (McFarlane 1994) | Title and outcome |
|  | (Lee, Joo, et al. 2019) | Title, outcome and study area |
|  | (Kukihara et al. 2014) | Title and outcome |
|  | (Seng et al. 2011) | Title and outcome |
|  | (Powell, Ekin-Wood, and Collin 2007) | Title and outcome |
|  | (Wilson and Zigelbaum 1983) | Title and outcome |
|  | (Perrin et al. 2014) | Title and outcome |
|  | (Beck et al. 2008) | Title and outcome |
|  | (Bisson and Deahl 1994) | Title and outcome |
|  | (AlShardan et al. 2020) | Title and outcome |
|  | (Bennice et al. 2003) | Title and outcome |
|  | (Palyo and Beck 2005) | Title and outcome |
|  | (Feinstein and Dolan 1991) | Title and outcome |
|  | (Wahab et al. 2021) | Title and outcome |
|  | (Joseph, Yule, and Williams 1993) | Title and outcome |
|  | (Patterson et al. 1990) | Title and outcome |
|  | (Saunders, Arata, and Kilpatrick 1990) | Title and outcome |
|  | (Stallard et al. 2001) | Title and outcome |
|  | (Tang, Pan, et al. 2017) | Title and outcome |
|  | (Trickey et al. 2012) | Title and abstract |
|  | (Ressler et al. 2022) | Title and outcome |
|  | (Ratnani) | Study area |
|  | (Gavranidou and Rosner 2003) | Title and outcome |
|  | (Schnyder, Moergeli, Trentz, et al. 2001) | Title and outcome |
|  | (Deja et al. 2006) | Title and outcome |
|  | (Simani et al. 2021) | Title and abstract |
|  | (Iz et al. 2019) | Title and abstract |
|  | (Asmundson et al. 2003) | Title and abstract |
|  | (Taubman-Ben-Ari et al. 2001) | Title and abstract |
|  | (McLean and Foa 2011) | Title and outcome |
|  | (Altaf et al. 2021) | Title and outcome |
|  | (Parikh et al. 2015) | Title and outcome |
|  | (Wadsworth, Santiago, and Einhorn 2009) | Title and outcome |
|  | (Başoğlu et al. 1997) | Title and outcome |
|  | (Yaalaoui et al. 2002) | Title and outcome |
|  | (Weems et al. 2010) | Title and outcome |
|  | (Hapke et al. 2006) | Title and outcome |
|  | (CHOU et al. 2007) | Title and outcome |
|  | (Neugebauer et al. 2009) | Title and outcome |
|  | (Silver 2014) | Title and outcome |
|  | (Thapa et al. 2018) | Title and outcome |
|  | (Thabet and Vostanis 1999) | Title and outcome |
|  | (Leymann and Gustafsson 1996) | Title and outcome |
|  | (Alway et al. 2016) | Title and outcome |
|  | (Ochberg 2013) | Title and outcome |
|  | (Yang et al. 2020) | Title and outcome |
|  | (Tucker et al. 2000) | Title and outcome |
|  | (Gilpin and Weiner 2017) | Title and outcome |
|  | (Mitchell and Everly Jr 1995) | Title and outcome |
|  | (Young 1997) | Title and outcome |
|  | (Elsesser and Sartory 2007) | Title and outcome |
|  | (Edmondson and von Känel 2017) | Title and outcome |
|  | (Schultebraucks et al. 2020) | Title and outcome |
|  | (Bryant et al. 2004) | Title and outcome |
|  | (Barawi et al. 2020) | Title and outcome |
|  | (Merecz, Waszkowska, and Wezyk 2012) | Title and outcome |
|  | (Feldner, Babson, and Zvolensky 2007) | Title and outcome |
|  | (Kennedy and Duff 2001) | Study area |
|  | (Johnsen et al. 2002) | Title and outcome |
|  | (Onyencho, Omeiza, and Wakil 2014) | Title and outcome |
|  | (Schindel-Allon et al. 2010) | Title and outcome |
|  | (Mayou and Bryant 2002) | Study area |
|  | (Cordova, Riba, and Spiegel 2017) | Title and outcome |
|  | (Bolton, Holohan, et al. 2004) | Title and outcome |
|  | (Maes, Lin, et al. 1998) | Title and outcome |
|  | (Maercker and Augsburger 2022) | Title and outcome |
|  | (Gorst-Unsworth and Goldenberg 1998) | Title and outcome |
|  | (Zhang et al. 2011) | Title and outcome |
|  | (Miniati et al. 2021) | Study area |
|  | (John, Russell, and Russell 2007) | Title and outcome |
|  | (Carmassi et al. 2021) | Title and outcome |
|  | (Carlier, Voerman, and Gersons 2000) | Title and outcome |
|  | (Rissanen, Berg, and Hasselberg 2017) | Title and outcome |
|  | (Daud, Skoglund, and Rydelius 2005) | Title and outcome |
|  | (Maes et al. 2000) | Title and outcome |
|  | (Logue et al. 2013) | Title and outcome |
|  | (Cohen and Scheeringa 2009) | Title and outcome |
|  | (Kokai et al. 2004) | Title and outcome |
|  | (Roberts et al. 2011) | Title and outcome |
|  | (Ma et al. 2011) | Title and outcome |
|  | (Hall, Hoerster, and Yancy Jr 2015) | Title and outcome |
|  | (Creamer, Burgess, and McFarlane 2001) | Title and outcome |
|  | (Speckens et al. 2007) | Title and outcome |
|  | (Li et al. 2023) | Title, outcome and study area |
|  | (Jin, Xu, et al. 2014) | Title, outcome and study area |
|  | (Eriksson and Lundin 1996) | Title, outcome and study area |
|  | (Kaplan, Vasterling, and Vedak 2010) | Title and outcome |
|  | (Schnyder, Moergeli, Klaghofer, et al. 2001) | Title and outcome |
|  | (Coughlin 2011) | Title and outcome |
|  | (Geng et al. 2013) | Title and outcome |
|  | (Schwarz and Perry 1994) | Title and outcome |
|  | (Herrera-Escobar et al. 2021) | Title, outcome and study area |
|  | (Neylan et al. 2021) | Title and outcome |
|  | (Ruini, Vescovelli, and Albieri 2013) | Title and outcome |
|  | (Kiser et al. 1991) | Title and outcome |
|  | (Lowenstein 2001) | Study area |
|  | (Xiong et al. 2010) | Title, outcome and study area |
|  | (Glaesmer et al. 2010) | Title and outcome |
|  | (Yang et al. 2022) | Title and outcome |
|  | (Brewin 1998) | Title and outcome |
|  | (McLay et al. 2011) | Title and outcome |
|  | (Figley 2014b) | Title and outcome |
|  | (Brooks et al. 2020) | Title and outcome |
|  | (Schnyder et al. 2008) | Title and outcome |
|  | (Foley, Hassett, and Williams 2022) | Title and outcome |
|  | (Garrison et al. 1993) | Title and outcome |
|  | (Igreja et al. 2004) | Title and outcome |
|  | (Sijbrandij et al. 2013) | Title and outcome |
|  | (Christiani and Yappo 2023) | Title and outcome |
|  | (Dansky, Byrne, and Brady 1999) | Title and outcome |
|  | (Acarturk et al. 2016) | Title and outcome |
|  | (Green and Berlin 1987) | Title and outcome |
|  | (Young and Blake 2020) | Title and outcome |
|  | (Blanchard and Veazey 2001) | Title and outcome |
|  | (Figley 1988) | Title and outcome |
|  | (Azoulay et al. 2005) | Title and outcome |
|  | (Kazantzis et al. 2012) | Study area |
|  | (Qiu et al. 2021) | Title and outcome |
|  | (Ursano et al. 1999) | Study area |
|  | (Kamkuimo, Girard, and Menelas 2021) | Title and outcome |
|  | (Zhang et al. 2014) | Title, outcome and study area |
|  | (Czarnocka and Slade 2000) | Title and outcome |
|  | (Stallard, Velleman, and Baldwin 1999) | Title and outcome |
|  | (Krysinska and Lester 2010) | Title and outcome |
|  | (Chung et al. 2004) | Title and outcome |
|  | (Cohen et al. 2006) | Title and outcome |
|  | (Fu et al. 2007) | Title and outcome |
|  | (PRAKASAM et al. 2020) | Title and outcome |
|  | (Krakow et al. 2000) | Title and outcome |
|  | (Bolton et al. 2000) | Title and outcome |
|  | (D'Souza 1995) | Title and outcome |
|  | (Norris 1990) | Title and outcome |
|  | (Symes 1995) | Title and outcome |
|  | (Abenhaim, Dab, and Salmi 1992) | Title and outcome |
|  | (Friedman, Schnurr, and McDonagh-Coyle 1994) | Title and outcome |
|  | (Davydow et al. 2008) | Title and outcome |
|  | (Meewisse et al. 2007) | Title and outcome |
|  | (Murphy et al. 2017) | Title and outcome |
|  | (Ahmed 2007) | Title and outcome |
|  | (Green 1993) | Title and outcome |
|  | (Solomon, Mikulincer, and Benbenishty 1989) | Title and outcome |
|  | (Tang et al. 2020) | Title and outcome |
|  | (Bryant and Harvey 1995c) | Title and outcome |
|  | (Berninger et al. 2010) | Title and outcome |
|  | (Schäfer et al. 2019) | Title and outcome |
|  | (Bromet 2012) | Title and outcome |
|  | (Ostrowski and Delahanty 2014) | Title and outcome |
|  | (Liberzon and Abelson 2016) | Title and outcome |
|  | (Du et al. 2022) | Title and outcome |
|  | (Buckley, Blanchard, and Hickling 1998) | Title and outcome |
|  | (Connor and Davidson 2001) | Title and outcome |
|  | (Chen et al. 2020) | Title and outcome |
|  | (Wu 2011) | Title and outcome |
|  | (Angel et al. 2014) | Title and outcome |
|  | (Fitzharris, Fildes, and Charlton 2006) | Study area |
|  | (Breslau et al. 2004) | Title and outcome |
|  | (Ehde et al. 2000) | Title and outcome |
|  | (Mayou and Bryant 2003) | Title and outcome |
|  | (De Bellis and Van Dillen 2005) | Title and outcome |
|  | (Wiederhold and Wiederhold 2010) | Title and outcome |
|  | (Chin et al. 2017) | Title and outcome |
|  | (Mcleer et al. 1988) | Title and outcome |
|  | (Yule and Williams 1990) | Title and outcome |
|  | (Suliman et al. 2013) | Title and outcome |
|  | (Perry 2007) | Title and outcome |
|  | (Moeller-Bertram, Keltner, and Strigo 2012) | Title and outcome |
|  | (Muldoon and Lowe 2012) | Title and outcome |
|  | (Seedat et al. 2004) | Title and outcome |
|  | (Van Praag et al. 2019) | Title and outcome |
|  | (Rose et al. 1999) | Title and outcome |
|  | (Beiser, Wiwa, and Adebajo 2010) | Title and outcome |
|  | (Mikkelsen et al. 2012) | Title and outcome |
|  | (Jonsson and Segesten 2004) | Title and outcome |
|  | (Bryant and Harvey 2003) | Title and outcome |
|  | (Shalev 2009) | Title and outcome |
|  | (Otis, Keane, and Kerns 2003) | Title and outcome |
|  | (Andualem et al. 2024) | Title and outcome |
|  | (Kramer et al. 1994) | Title, outcome and study area |
|  | (Begić and Jokić-Begić 2001) | Title and outcome |
|  | (Bae, Hyun, and Lee 2014) | Title and outcome |
|  | (McFarlane and Bryant 2007) | Title and outcome |
|  | (Bryant and Harvey 1996a) | Title and outcome |
|  | (Davidson et al. 1991) | Title and outcome |
|  | (Mayou 2021) | Title and outcome |
|  | (Alonzo 2000) | Title and outcome |
|  | (Johannesson et al. 2011) | Title and outcome |
|  | (Meiser-Stedman et al. 2009) | Title and outcome |
|  | (Curle and Williams 1996) | Title and outcome |
|  | (Kravets et al. 2023) | Study area |
|  | (Solomon, Dekel, and Mikulincer 2008) | Title and outcome |
|  | (Duan, Guo, and Gan 2015) | Title and outcome |
|  | (Yule et al. 2000) | Title and outcome |
|  | (Anderson 2007) | Title and outcome |
|  | (Gillespie et al. 2002) | Title and outcome |
|  | (Shen et al. 2021) | Title, outcome and study area |
|  | (Markowitz et al. 2009) | Title and outcome |
|  | (Chung and Breslau 2008) | Title and outcome |
|  | (Haagsma et al. 2015) | Title and outcome |
|  | (Figley 2013b) | Title and outcome |
|  | (Richardson, Frueh, and Acierno 2010) | Title and outcome |
|  | (Tareen, Garralda, and Hodes 2007) | Title and outcome |
|  | (Watson 1987) | Title, outcome and study area |
|  | (Ohry, Rattok, and Solomon 1996) | Title and outcome |
|  | (Wang et al. 2009) | Title and outcome |
|  | (Bryant and Harvey 2002) | Title and outcome |
|  | (Clohessy and Ehlers 1999) | Title and outcome |
|  | (Sun et al. 2013) | Title and outcome |
|  | (Barskova and Oesterreich 2009) | Title and outcome |
|  | (Herman 1992) | Title, outcome and study area |
|  | (Perrin, Smith, and Yule 2000) | Title and abstract |
|  | (Koch 2002) | Title and abstract |
|  | (Kilpatrick and Williams 1997) | Title and abstract |
|  | (Murray and Ehlers 2021) | Title and outcome |
|  | (Memarzia, Walker, and Meiser-Stedman 2021) | Title and outcome |
|  | (Gersons and Carlier 1992) | Title and outcome |
|  | (Jenewein et al. 2009) | Title and outcome |
|  | (Campanini et al. 2010) | Title and outcome |
|  | (Mayou, Ehlers, and Hobbs 2000) | Title and outcome |
|  | (Panagioti, Gooding, and Tarrier 2009) | Title and outcome |
|  | (Riggs, Rothbaum, and Foa 1995) | Title and outcome |
|  | (Stein et al. 2007) | Title and outcome |
|  | (Thompson et al. 1995) | Title and outcome |
|  | (Lisieski et al. 2018) | Title and outcome |
|  | (Nutt et al. 2009) | Study area |
|  | (Zhou, Wu, and Zhen 2018) | Title and outcome |
|  | (Liao et al. 2019) | Title and outcome |
|  | (Murray and Murray 1997) | Title and outcome |
|  | (Kang et al. 2024) | Title and outcome |
|  | (Weisæth 1989a) | Title and outcome |
|  | (Nicholl and Thompson 2004) | Title and outcome |
|  | (Fichtenberg et al. 2000) | Title and outcome |
|  | (Mrdjenovich 2018) | Title and outcome |
|  | (Rodríguez‐Muñoz et al. 2010) | Title and outcome |
|  | (Beck 2004) | Title and outcome |
|  | (Taal and Faber 1997) | Title and outcome |
|  | (Walker 1991) | Title and outcome |
|  | (Emilien 2000) | Title and outcome |
|  | (Famularo et al. 1996) | Title and outcome |
|  | (Kumar and Thakur) | Title and outcome |
|  | (Joseph et al. 1997) | Title and outcome |
|  | (Bryant and Harvey 1999b) | Title and outcome |
|  | (Zoladz and Diamond 2013) | Title and outcome |
|  | (Reynolds et al. 2005) | Title and outcome |
|  | (Wild et al. 2016) | Title and outcome |
|  | (Green 1994) | Title and outcome |
|  | (Schubert, Schmidt, and Rosner 2016) | Title and outcome |
|  | (Lewis et al. 2019) | Title, outcome and study area |
|  | (Alpak et al. 2015) | Title and outcome |
|  | (McFarlane et al. 1994) | Title and outcome |
|  | (Boris, Ou, and Singh 2005) | Title and outcome |
|  | (Admon, Milad, and Hendler 2013) | Title and outcome |
|  | (Marais and Stuart 2005) | Title and outcome |
|  | (Lewis et al. 2019) | Title, outcome and study area |
|  | (Holeva, Tarrier, and Wells 2001) | Study area |
|  | (McFarlane et al. 1994) | Title and outcome |
|  | (Boris, Ou, and Singh 2005) | Title and outcome |
|  | (Admon, Milad, and Hendler 2013) | Title and outcome |
|  | (Silove et al. 2002) | Title and outcome |
|  | (Nichols and Czirr 1986) | Title and outcome |
|  | (Kılıç, Magruder, and Koryürek 2016) | Title and outcome |
|  | (Serrano-Ibanez et al. 2023) | Title and outcome |
|  | (Wilson, Dzansi, and Ohene 2020) | Title and outcome |
|  | (Amick‐McMullan et al. 1989) | Title and outcome |
|  | (Kanani, Hadi, and Tayebi 2015) | Title and outcome |
|  | (Nakell 2007) | Title and outcome |
|  | (Menage 1993) | Title and outcome |
|  | (Kupchik et al. 2007) | Title and outcome |
|  | (Memarzia et al. 2024) | Title and outcome |
|  | (Spinhoven et al. 2015) | Title and outcome |
|  | (Kinchin 2007) | Title and outcome |
|  | (Figley 2013a) | Title and outcome |
|  | (Wiseman, Foster, and Curtis 2013) | Title and outcome |
|  | (Stark et al. 2015) | Title and outcome |
|  | (Maercker and Müller 2004) | Title and outcome |
|  | (Shakespeare-Finch et al. 2003) | Title and outcome |
|  | (Delahanty et al. 2003) | Title and outcome |
|  | (Hamanaka et al. 2006) | Title and outcome |
|  | (Bracken 2001) | Title and outcome |
|  | (De Boer et al. 2011) | Title and outcome |
|  | (Ecrepont et al. 2016) | Title and outcome |
|  | (Zen et al. 2012) | Title and outcome |
|  | (Messo 2013) | Title and outcome |
|  | (Norberg, Pöder, and von Essen 2011) | Title and outcome |
|  | (Steinglass and Gerrity 1990) | Title and outcome |
|  | (Kenardy and Dunne 2011) | Title and outcome |
|  | (Bryant and Harvey 1995b) | Title and outcome |
|  | (Bui et al. 2011) | Title and outcome |
|  | (Bordbar 2007) | Title and outcome |
|  | (Smyth, Hockemeyer, and Tulloch 2008) | Title and outcome |
|  | (Fecteau 2001) | Title and outcome |
|  | (Vaiva et al. 2004) | Title and outcome |
|  | (McFarlane 1989) | Title and outcome |
|  | (Lohr et al. 2015) | Title and outcome |
|  | (Davidson and Smith 1990) | Title and outcome |
|  | (Howlett and Stein 2015) | Title and outcome |
|  | (Deng et al. 2016) | Title and outcome |
|  | (Kılıç et al. 2006) | Title and outcome |
|  | (Cantor and Price 2007) | Title and outcome |
|  | (Neal et al. 1994) | Title and outcome |
|  | (Goenjian et al. 1994) | Title and outcome |
|  | (Fischer et al. 2021) | Title and outcome |
|  | (Chibnall and Duckro 1994) | Title and outcome |
|  | (McFarlane 1987) | Title and outcome |
|  | (Kim et al. 2025) | Title and outcome |
|  | (Michaels et al. 1999) | Title and outcome |
|  | (Foa and Riggs 1995) | Title and outcome |
|  | (Dell’Osso et al. 2014) | Title and outcome |
|  | (Zeidner and Ben-Zur 1994) | Title and outcome |
|  | (Uddo et al. 1993) | Title and outcome |
|  | (Reynolds et al. 2016) | Title and outcome |
|  | (Zhang and Ho 2011) | Title, outcome and study area |
|  | (Park et al. 2020) | Title, outcome and study area |
|  | (Alanazi et al. 2021) | Title and outcome |
|  | (Karunakara et al. 2004) | Title and outcome |
|  | (Breen et al. 2015) | Title and outcome |
|  | (Difede and Hoffman 2002) | Title and outcome |
|  | (Bryant 1996) | Title and outcome |
|  | (Menelas et al. 2018) | Title and outcome |
|  | (Wentworth et al. 2013) | Title and outcome |
|  | (Khamis 2005) | Title, outcome and study area |
|  | (Roussis and Wells 2006) | Title and outcome |
|  | (Forbes et al. 2007) | Title and outcome |
|  | (Yrondi et al. 2019) | Title and outcome |
|  | (Shalev et al. 1996) | Study area |
|  | (Crumlish and O'Rourke 2010) | Title and outcome |
|  | (Back et al. 2000) | Title and outcome |
|  | (Söderquist, Wijma, and Wijma 2006) | Title and outcome |
|  | (Sullivan et al. 2009) | Title and outcome |
|  | (Fung et al. 2022) | Title and outcome |
|  | (Almutairi and Altamimi 2019) | Title and outcome |
|  | (Högberg et al. 2007) | Title and outcome |
|  | (Goulston 2012) | Title and outcome |
|  | (Eid, Thayer, and Johnsen 1999) | Title and outcome |
|  | (Matthiesen and Einarsen 2004) | Title and outcome |
|  | (Petrowski et al. 2020) | Title and outcome |
|  | (Delahanty et al. 1997) | Title and outcome |
|  | (Sbordone 1999) | Title and outcome |
|  | (Tarrier et al. 2000) | Title and outcome |
|  | (Bromet et al. 2016) | Title and outcome |
|  | (Nishi, Usuki, and Matsuoka 2012) | Title and outcome |
|  | (Brandes et al. 2002) | Title and outcome |
|  | (Kim et al. 2024) | Study area |
|  | (Angel 2005) | Title and outcome |
|  | (Ravn et al. 2018) | Title and outcome |
|  | (Kaminer, Seedat, and Stein 2005) | Title and outcome |
|  | (Elder et al. 2012) | Title and outcome |
|  | (Weisæth 1989c) | Title and outcome |
|  | (Sonne et al. 2003) | Title and outcome |
|  | (Avrahami 2006) | Title and outcome |
|  | (Silverman 1986) | Title and outcome |
|  | (Figley 2014a) | Title and outcome |
|  | (Meiser‐Stedman et al. 2009) | Title and outcome |
|  | (Schöner et al. 2017) | Title and outcome |
|  | (Fernandez 2007) | Title and outcome |
|  | (Dinnen, Simiola, and Cook 2015) | Title and outcome |
|  | (Boscarino 1995) | Title and outcome |
|  | (Osenbach et al. 2014) | Title and outcome |
|  | (Almqvist and Brandell-Forsberg 1997) | Title and outcome |
|  | (Villain et al. 2018) | Title and outcome |
|  | (Dückers, Alisic, and Brewin 2016) | Title and outcome |
|  | (Husna and Kuswoyo 2022) | Title and outcome |
|  | (Williams, Cahill, and Foa 2010) | Title and outcome |
|  | (McDermott and Cvitanovich 2000) | Study area |
|  | (McFarlane 2010) | Title and outcome |
|  | (Jones, Harvey, and Brewin 2005) | Title and outcome |
|  | (Flatten, Wälte, and Perlitz 2008) | Title and outcome |
|  | (Regel 2007) | Title and outcome |
|  | (Colville and Pierce 2012) | Title and outcome |
|  | (Schuster and Dwyer 2020) | Title and outcome |
|  | (Spence Laschinger and Nosko 2015) | Title and outcome |
|  | (Wang et al. 2010) | Title and outcome |
|  | (Lewis, Roberts, Andrew, et al. 2020) | Title and outcome |
|  | (Beck and Coffey 2007) | Title and outcome |
|  | (Smith et al. 2014) | Title and outcome |
|  | (Wilcoxon, Meiser-Stedman, and Burgess 2021) | Title and outcome |
|  | (Gupta 2013) | Title and outcome |
|  | (Amos, Stein, and Ipser 2014) | Title and outcome |
|  | (Sumner et al. 2016) | Title and outcome |
|  | (Stankovic 2011) | Title and outcome |
|  | (Rogers et al. 2009) | Title and outcome |
|  | (Blaszczynski et al. 1998) | Title and outcome |
|  | (Berton and Stabb 1996) | Title and outcome |
|  | (McFetridge et al. 2017) | Title and outcome |
|  | (Andrikopoulos and Greiffenstein 2012) | Title and outcome |
|  | (Miller and Harrington 2011) | Title and outcome |
|  | (Ehlers, Mayou, and Bryant 2003) | Title and outcome |
|  | (Marina and Michael 2013) | Study area |
|  | (Husain, Miller, and Carwile 2001) | Title and outcome |
|  | (Egan, Hattaway, and Kane 2014) | Title and outcome |
|  | (Oquendo et al. 2003) | Title and outcome |
|  | (O'Donohue and Elliott 1992) | Title and outcome |
|  | (Van Emmerik et al. 2002) | Title and outcome |
|  | (van Der Kolk, Ford, and Spinazzola 2019) | Title and outcome |
|  | (Palm, Polusny, and Follette 2004) | Title and outcome |
|  | (Udomratn 2008) | Title and outcome |
|  | (Villamor and Sáez de Adana 2014) | Title and outcome |
|  | (Amir et al. 1997) | Title and outcome |
|  | (Rogers and Read 2007) | Title and outcome |
|  | (Brown et al. 2014) | Title and outcome |
|  | (Bryant et al. 2008) | Title and outcome |
|  | (Pierce et al. 2023) | Title and outcome |
|  | (Hartley et al. 2013) | Title and outcome |
|  | (Spitzer et al. 2001) | Title and outcome |
|  | (Lewis, Roberts, Gibson, et al. 2020) | Title and outcome |
|  | (Sheikhbardsiri et al. 2015) | Title and outcome |
|  | (Vincent et al. 2015) | Title and outcome |
|  | (Yehuda 1998) | Title and outcome |
|  | (De Bellis and Thomas 2003) | Title and outcome |
|  | (Brewin, Andrews, and Rose 2000) | Title and outcome |
|  | (Burges and McMillan 2001) | Title and outcome |
|  | (Crabbe et al. 2004) | Title and outcome |
|  | (Stevens and Jovanovic 2019) | Title and outcome |
|  | (Joseph, Williams, and Yule 1992) | Title and outcome |
|  | (Ryan et al. 2016) | Title and outcome |
|  | (Davidson 2000) | Title and outcome |
|  | (Holmes and North 1998) | Title and outcome |
|  | (Kilpatrick and Williams 1998) | Title and outcome |
|  | (Bolton, Hill, et al. 2004) | Title and outcome |
|  | (Jowett, Karatzias, and Albert 2020) | Title and outcome |
|  | (Realmuto et al. 1992) | Title and outcome |
|  | (Breslau and Schultz 2013) | Title and outcome |
|  | (Wilson, Smith, and Johnson 2013) | Title and outcome |
|  | (Keane et al. 2013) | Title and outcome |
|  | (Ma et al. 2017) | Title and outcome |
|  | (Priebe et al. 2009) | Title and outcome |
|  | (Gamper et al. 2004) | Title and outcome |
|  | (Machisa, Christofides, and Jewkes 2018) | Title and outcome |
|  | (Kar 2009) | Title and outcome |
|  | (Brijnath et al. 2016) | Title and outcome |
|  | (Holeva and Tarrier 2001) | Title and outcome |
|  | (Medina-Ortiz et al. 2023) | Title and outcome |
|  | (Mak et al. 2009) | Title and outcome |
|  | (Djelantik et al. 2017) | Title and outcome |
|  | (Joseph, Murphy, and Regel 2012) | Title and outcome |
|  | (Altindag, Ozen, and Sir 2005) | Title and outcome |
|  | (Parker et al. 2015) | Title and outcome |
|  | (Chen and Zhao 2019) | Title and outcome |
|  | (Lee, Faber, and Bowles 2022) | Title and outcome |
|  | (Mayou, Black, and Bryant 2000) | Title, outcome and study area |
|  | (Green et al. 1994) | Title and outcome |
|  | (Resick and Miller 2009) | Title and outcome |
|  | (Andreski, Chilcoat, and Breslau 1998) | Title and outcome |
|  | (Arnsten et al. 2015) | Title and outcome |
|  | (Dyregrov, Nordanger, and Dyregrov 2003) | Title and outcome |
|  | (Dekel and Monson 2010) | Title and outcome |
|  | (Mayou and Bryant 2001) | Title and outcome |
|  | (Olliac et al. 2014) | Title and outcome |
|  | (Gillies et al. 2013) | Title and outcome |
|  | (Roop 2006) | Title and outcome |
|  | (McFarlane, Williamson, and Barton 2009) | Title and outcome |
|  | (McElheran et al. 2012) | Title and abstract |
|  | (Bremner 2002) | Title and abstract |
|  | (Williams et al. 2011) | Title and outcome |
|  | (Koenen et al. 2017) | Title and outcome |
|  | (Thabet and Vostanis 2000) | Title and outcome |
|  | (Brunetti et al. 2010) | Title abstract |
|  | (Roth, Geisser, and Bates 2008) | Title and abstract |
|  | (Douglas 1999) | Title and outcome |
|  | (Sembi et al. 1998) | Title and outcome |
|  | (Engelhard, van den Hout, and Kindt 2003) | Title and abstract |
|  | (Pagel 2021) | Title and abstract |
|  | (Bryant, Moulds, and Nixon 2003) | Title and abstract |
|  | (Pan, Kaminga, et al. 2018) | Title and abstract |
|  | (Başoǧlu et al. 2004) | Title and abstract |
|  | (Burrai et al. 2021) | Title and abstract |
|  | (Baird and Kracen 2006) | Title and abstract |
|  | (Canterbury and Yule 2021) | Title and outcome |
|  | (Kazour et al. 2017) | Title and outcome |
|  | (Henson, Truchot, and Canevello 2021) | Title and outcome |
|  | (Jin, Qi, et al. 2014) | Title and outcome |
|  | (Pagel 2021) | Title and outcome |
|  | (Bryant, Moulds, and Nixon 2003) | Title and outcome |
|  | (Pan, Kaminga, et al. 2018) | Title and outcome |
|  | (Başoǧlu et al. 2004) | Title and outcome |
|  | (Burrai et al. 2021) | Title and outcome |
|  | (Baird and Kracen 2006) | Title and outcome |
|  | (Murdoch et al. 2006) | Title |
|  | (Canterbury and Yule 2021) | Title and outcome |
|  | (Cantor 2009) | Title and outcome |
|  | (Kazour et al. 2017) | Title |
|  | (Henson, Truchot, and Canevello 2021) | Title |
|  | (Yehuda, McFarlane, and Shalev 1998) | Title and outcome |
|  | (Brief et al. 2004) | Title and outcome |
|  | (Kira 2010) | Title and abstract |
|  | (Bremner 2006) | Title and abstract |
|  | (Boumpa et al. 2024) | Title and outcome |
|  | (Bryant and Harvey 1998) | Title and abstract |
|  | (Collicutt McGrath and Linley 2006) | Title |
|  | (Jansen et al. 2011) | Title and outcome |
|  | (El Khoury-Malhame et al. 2011) | Title |
|  | (Farley et al. 2007) | Title |
|  | (Bockhop et al. 2023) | Title |
|  | (Paunovic 1998) | Title and abstract |
|  | (Fani et al. 2012) | Title and abstract |
|  | (Alfheim et al. 2019) | Title and outcome |
|  | (El Khoury-Malhame et al. 2011) | Title and outcome |
|  | (Farley et al. 2007) | Title and outcome |
|  | (Bockhop et al. 2023) | Title and abstract |
|  | (Paunovic 1998) | Title and abstract |
|  | (Fani et al. 2012) | Title and abstract |
|  | (Langford et al. 2018) | Title and outcome |
|  | (Alfheim et al. 2019) | Title and outcome |
|  | (Pires and Maia 2013) | Study area |
|  | (Chen et al. 2009) | Title and abstract |
|  | (King et al. 2001) | Title |
|  | (Truszczyńska-Baszak et al. 2023) | Title |
|  | (Zatzick et al. 2002) | Title and outcome |
|  | (Pitman et al. 2012) | Title and outcome |
|  | (Lawrence and Fauerbach 2003) | Title and outcome |
|  | (McLaughlin et al. 2017) | Title and abstract |
|  | (Cipriano Jr 2001) | Title and abstract |
|  | (Wilson 1994) | Title and abstract |
|  | (Moor and Farchi 2011) | Title and outcome |
|  | (Wang et al. 2017) | Title |
|  | (Lindauer et al. 2005) | Title |
|  | (Zoladz et al. 2008) | Title and abstract |
|  | (Giannopoulou et al. 2006) | Title and abstract |
|  | (Berardi, Schelling, and Campolongo 2016) | Title and abstract |
|  | (Abolhadi et al. 2022) | Title and abstract |
|  | (Bryant and Harvey 1997) | Title and abstract |
|  | (Walshe et al. 2003) | Title and outcome |
|  | (Antolasic, Jaehne, and van den Buuse 2024) | Title and outcome |
|  | (Ścigała and Zdankiewicz-Ścigała 2019) | Title and outcome |
|  | (Nicholls and Ayers 2007) | Title and outcome |
|  | (Masuwa 2017) | Title and abstract |
|  | (Bryant and Harvey 1996b) | Title and abstract |
|  | (Matu 2022) | Title and outcome |
|  | (Varma et al. 2024) | Title and outcome |
|  | (Stewart et al. 2004) | Title and outcome |
|  | (Kim 2022) | Title, outcome and study area |
|  | (Gabriel et al. 2007) | Title and outcome |
|  | (Doustahadi et al. 2023) | Title and outcome |
|  | (Pan, Wang, et al. 2018) | Title and outcome |
|  | (Zhang et al. 2010) | Title and abstract |
|  | (Bisson 2007) | Title and abstract |
|  | (Ehring, Ehlers, and Glucksman 2008) | Title and outcome |
|  | (Ferree, Wheeler, and Cahill 2012) | Title |
|  | (Koenigs et al. 2008) | Title and outcome |
|  | (Eisenbruch 1991) | Title |
|  | (Freeman et al. 2013) | Title and abstract |
|  | (Whiting and Bryant 2007) | Title and outcome |
|  | (Lever et al. 2019) | Title and outcome |

**References**

Abbey, Gareth, Simon BN Thompson, Tamas Hickish, and David Heathcote. 2015. 'A meta‐analysis of prevalence rates and moderating factors for cancer‐related post‐traumatic stress disorder', *Psycho‐Oncology*, 24: 371-81.

Abenhaim, Lucien, William Dab, and L Rachid Salmi. 1992. 'Study of civilian victims of terrorist attacks (France 1982–1987)', *Journal of Clinical Epidemiology*, 45: 103-09.

Abolhadi, Elham, Parisa Divsalar, Mohammad Amin Mosleh-Shirazi, and Tania Dehesh. 2022. 'Latent classes of posttraumatic stress disorder among survivors of the Bam Earthquake after 17 years', *BMC psychiatry*, 22: 603.

Acarturk, Ceren, Emre Konuk, M Cetinkaya, Ibrahim Senay, Marit Sijbrandij, B Gulen, and Pim Cuijpers. 2016. 'The efficacy of eye movement desensitization and reprocessing for post-traumatic stress disorder and depression among Syrian refugees: Results of a randomized controlled trial', *Psychological medicine*, 46: 2583-93.

Ackerman, Peggy T, Joseph EO Newton, W Brian McPherson, Jerry G Jones, and Roscoe A Dykman. 1998. 'Prevalence of post traumatic stress disorder and other psychiatric diagnoses in three groups of abused children (sexual, physical, and both)', *Child abuse & neglect*, 22: 759-74.

Adhikari Baral, Ishwari, and Bhagawati KC. 2019. 'Post traumatic stress disorder and coping strategies among adult survivors of earthquake, Nepal', *BMC psychiatry*, 19: 1-8.

Admon, Roee, Mohammed R Milad, and Talma Hendler. 2013. 'A causal model of post-traumatic stress disorder: disentangling predisposed from acquired neural abnormalities', *Trends in cognitive sciences*, 17: 337-47.

Ahmed, Ayesha S. 2007. 'Post-traumatic stress disorder, resilience and vulnerability', *Advances in Psychiatric treatment*, 13: 369-75.

Alanazi, Sami Eid, Irfan Anjum, Ahmed Samer Almohna, Hamoud Mohammed Alfawzan, Sultan M Albugami, and Mohammad Nasser Almutairi. 2021. 'Prevalence of post-traumatic stress disorder after road traffic accident at King Abdulaziz Medical City, Riyadh', *Age (years)*, 36: 17.

Alfheim, Hanne Birgit, Kristin Hofsø, Milada Cvancarova Småstuen, Kirsti Tøien, Leiv Arne Rosseland, and Tone Rustøen. 2019. 'Post-traumatic stress symptoms in family caregivers of intensive care unit patients: A longitudinal study', *Intensive and Critical Care Nursing*, 50: 5-10.

Ali, Dawed, Telake Azale, Melese Wondie, and Jinenus Tadesse. 2022. 'About six in ten survivors of the november 2020 Maikadra massacre suffer from posttraumatic stress disorder, northwest Ethiopia', *Psychology research and behavior management*: 251-60.

Almqvist, Kjerstin, and Margareta Brandell-Forsberg. 1997. 'Refugee children in Sweden: Post-traumatic stress disorder in Iranian preschool children exposed to organized violence', *Child abuse & neglect*, 21: 351-66.

Almutairi, Noura Mesned, and Mohammed Abdulrahman Altamimi. 2019. 'The psychosocial consequences of road traffic accidents: a review article', *Int J Med Dev Ctries*, 3: 1104-9.

Alonzo, Angelo A. 2000. 'The experience of chronic illness and post-traumatic stress disorder: The consequences of cumulative adversity', *Social Science & Medicine*, 50: 1475-84.

Alpak, Gokay, Ahmet Unal, Feridun Bulbul, Eser Sagaltici, Yasin Bez, Abdurrahman Altindag, Alican Dalkilic, and Haluk A Savas. 2015. 'Post-traumatic stress disorder among Syrian refugees in Turkey: a cross-sectional study', *International journal of psychiatry in clinical practice*, 19: 45-50.

AlShardan, Ali Mesfer, Hassan Mudayni AlShuqaybi, Sajida Agha, Emad Masuadi, Ibrahim AlBabtain, Abdullah Sayer AlBugami, and Misaad Salem AlKhalidi. 2020. 'Assessment of sleep quality, post-traumatic stress disorder, and locus of control in motor vehicle accident survivors', *Australasian Medical Journal (Online)*, 13: 78-85.

Altaf, Rabail, Muhammad Rafique Shaikh, Asghar Ali Memon, Nargis Pirya, Farah Malik, and Muhammad Ayoub Jogi. 2021. 'Medico-legal cases (MLC) presented at a tertiary care center and acute symptoms of post-traumatic stress disorder among the survivors', *Pakistan Journal of Neurological Surgery*, 25: 263-70.

Altindag, Abdurrahman, Sakir Ozen, and Aytekin Sir. 2005. 'One-year follow-up study of posttraumatic stress disorder among earthquake survivors in Turkey', *Comprehensive psychiatry*, 46: 328-33.

Alway, Yvette, Kate Rachel Gould, Adam McKay, Lisa Johnston, and Jennie Ponsford. 2016. 'The evolution of post-traumatic stress disorder following moderate-to-severe traumatic brain injury', *Journal of neurotrauma*, 33: 825-31.

Amick‐McMullan, Angelynne, Dean G Kilpatrick, Lois J Veronen, and Susan Smith. 1989. 'Family survivors of homicide victims: Theoretical perspectives and an exploratory study', *Journal of Traumatic stress*, 2: 21-35.

Amir, M, Z Kaplan, R_ Efroni, Y Levine, J Benjamin, and M Kotler. 1997. 'Coping styles in post-traumatic stress disorder (PTSD) patients', *Personality and Individual Differences*, 23: 399-405.

Amir, Marianne, and Alona Ramati. 2002. 'Post-traumatic symptoms, emotional distress and quality of life in long-term survivors of breast cancer: a preliminary research', *Journal of anxiety disorders*, 16: 191-206.

Amos, Taryn, Dan J Stein, and Jonathan C Ipser. 2014. 'Pharmacological interventions for preventing post‐traumatic stress disorder (PTSD)', *Cochrane database of systematic reviews*.

Anderson, Jane M. 2007. 'Post-traumatic stress disorder recognized in victims of many traumas', *Journal of Controversial Medical Claims*, 14: 1-12.

Andreski, Patricia, Howard Chilcoat, and Naomi Breslau. 1998. 'Post-traumatic stress disorder and somatization symptoms: a prospective study', *Psychiatry research*, 79: 131-38.

Andrikopoulos, Jim, and Manfred F Greiffenstein. 2012. 'Something to talk about? The status of post-traumatic stress disorder in clinical neuropsychology'.

Andualem, Fantahun, Mamaru Melkam, Girmaw Medfu Takelle, Girum Nakie, Techilo Tinsae, Setegn Fentahun, Gidey Rtbey, Tesfaye Derbie Begashaw, Jemal Seid, and Lidiya Fasil Tegegn. 2024. 'Prevalence of posttraumatic stress disorder and associated factors among displaced people in Africa: a systematic review and meta-analysis', *Frontiers in psychiatry*, 15: 1336665.

Angel, Caroline M. 2005. *Crime victims meet their offenders: Testing the impact of restorative justice conferences on victims' post-traumatic stress symptoms* (University of Pennsylvania).

Angel, Caroline M, Lawrence W Sherman, Heather Strang, Barak Ariel, Sarah Bennett, Nova Inkpen, Anne Keane, and Therese S Richmond. 2014. 'Short-term effects of restorative justice conferences on post-traumatic stress symptoms among robbery and burglary victims: a randomized controlled trial', *Journal of Experimental Criminology*, 10: 291-307.

Antolasic, Emily J, Emily J Jaehne, and Maarten van den Buuse. 2024. 'Interaction of Brain-derived Neurotrophic Factor, Exercise, and Fear Extinction: Implications for Post-traumatic Stress Disorder', *Current neuropharmacology*, 22: 543-56.

Armenian, Haroutune K, Masahiro Morikawa, Arthur K Melkonian, Ashot P Hovanesian, Nune Haroutunian, Philip A Saigh, Knarig Akiskal, and Hagop S Akiskal. 2000. 'Loss as a determinant of PTSD in a cohort of adult survivors of the 1988 earthquake in Armenia: implications for policy', *Acta Psychiatrica Scandinavica*, 102: 58-64.

Armstrong, Deanne, Jane Shakespeare‐Finch, and Ian Shochet. 2014. 'Predicting post‐traumatic growth and post‐traumatic stress in firefighters', *Australian Journal of Psychology*, 66: 38-46.

Arnsten, Amy FT, Murray A Raskind, Fletcher B Taylor, and Daniel F Connor. 2015. 'The effects of stress exposure on prefrontal cortex: Translating basic research into successful treatments for post-traumatic stress disorder', *Neurobiology of stress*, 1: 89-99.

Arora, Deeksha, C Xavier Belsiyal, and Vikram Singh Rawat. 2021. 'Prevalence and determinants of posttraumatic stress disorder and depression among survivors of motor vehicle accidents from a hilly Indian state', *Indian journal of psychiatry*, 63: 250-57.

Asmundson, Gordon JG, Kristi D Wright, Donald R McCreary, and David Pedlar. 2003. 'Post-traumatic stress disorder symptoms in United Nations peacekeepers: An examination of factor structure in peacekeepers with and without chronic pain', *Cognitive Behaviour Therapy*, 32: 26-37.

Avrahami, Dalia. 2006. 'Visual art therapy's unique contribution in the treatment of post-traumatic stress disorders', *Journal of Trauma & Dissociation*, 6: 5-38.

Azoulay, Elie, Frédéric Pochard, Nancy Kentish-Barnes, Sylvie Chevret, Jérôme Aboab, Christophe Adrie, Djilali Annane, Gérard Bleichner, Pierre Edouard Bollaert, and Michael Darmon. 2005. 'Risk of post-traumatic stress symptoms in family members of intensive care unit patients', *American journal of respiratory and critical care medicine*, 171: 987-94.

Back, Sudie, Bonnie S Dansky, Scott F Coffey, Michael E Saladin, Susan Sonne, and Kathleen T Brady. 2000. 'Cocaine dependence with and without post-traumatic stress disorder: a comparison of substance use, trauma history and psychiatric comorbidity', *American Journal on Addictions*, 9: 51-62.

Bae, Sung-Man, Myoung-Ho Hyun, and Seung-Hwan Lee. 2014. 'Comparison of memory function and MMPI-2 profile between post-traumatic stress disorder and adjustment disorder after a traffic accident', *Clinical psychopharmacology and neuroscience*, 12: 41.

Baird, Katie, and Amanda C Kracen. 2006. 'Vicarious traumatization and secondary traumatic stress: A research synthesis', *Counselling Psychology Quarterly*, 19: 181-88.

Bal, Aydın. 2008. 'Post‐traumatic stress disorder in Turkish child and adolescent survivors three years after the Marmara earthquake', *Child and Adolescent Mental Health*, 13: 134-39.

Bal, Aydın, and Bryant Jensen. 2007. 'Post-traumatic stress disorder symptom clusters in Turkish child and adolescent trauma survivors', *European child & adolescent psychiatry*, 16: 449-57.

Barawi, Kali S, Catrin Lewis, Natalie Simon, and Jonathan I Bisson. 2020. 'A systematic review of factors associated with outcome of psychological treatments for post-traumatic stress disorder', *European Journal of Psychotraumatology*, 11: 1774240.

Barskova, Tatjana, and Rainer Oesterreich. 2009. 'Post-traumatic growth in people living with a serious medical condition and its relations to physical and mental health: A systematic review', *Disability and rehabilitation*, 31: 1709-33.

Barton, Kristine A, Edward B Blanchard, and Edward J Hickling. 1996. 'Antecedents and consequences of acute stress disorder among motor vehicle accident victims', *Behaviour research and therapy*, 34: 805-13.

Başoǧlu, Metin, Cengiz Kiliç, Ebru Şalcioǧlu, and Maria Livanou. 2004. 'Prevalence of posttraumatic stress disorder and comorbid depression in earthquake survivors in Turkey: an epidemiological study', *Journal of Traumatic Stress: Official Publication of The International Society for Traumatic Stress Studies*, 17: 133-41.

Başoğlu, Metin, S Mineka, Murat Paker, T Aker, Maria Livanou, and Ş Gök. 1997. 'Psychological preparedness for trauma as a protective factor in survivors of torture', *Psychological medicine*, 27: 1421-33.

Beck, Cheryl Tatano. 2004. 'Post-traumatic stress disorder due to childbirth: the aftermath', *Nursing research*, 53: 216-24.

Beck, J Gayle, and Scott F Coffey. 2007. 'Assessment and treatment of posttraumatic stress disorder after a motor vehicle collision: Empirical findings and clinical observations', *Professional Psychology: Research and Practice*, 38: 629.

Beck, J Gayle, DeMond M Grant, Jennifer P Read, Joshua D Clapp, Scott F Coffey, Luana M Miller, and Sarah A Palyo. 2008. 'The impact of event scale-revised: psychometric properties in a sample of motor vehicle accident survivors', *Journal of anxiety disorders*, 22: 187-98.

Bedard-Gilligan, Michele, and Lori A Zoellner. 2012. 'Dissociation and memory fragmentation in post-traumatic stress disorder: An evaluation of the dissociative encoding hypothesis', *Memory*, 20: 277-99.

Begić, Dražen, and Nataša Jokić-Begić. 2001. 'Aggressive behavior in combat veterans with post-traumatic stress disorder', *Military medicine*, 166: 671-76.

Beiser, Morton, Owens Wiwa, and Sylvia Adebajo. 2010. 'Human-initiated disaster, social disorganization and post-traumatic stress disorder above Nigeria’s oil basins', *Social Science & Medicine*, 71: 221-27.

Bennett, Paul, Roger L Owen, Stavroula Koutsakis, and Jonathon Bisson. 2002. 'Personality, social context and cognitive predictors of post-traumatic stress disorder in myocardial infarction patients', *Psychology and Health*, 17: 489-500.

Bennice, Jennifer A, Patricia A Resick, Mindy Mechanic, and Millie Astin. 2003. 'The relative effects of intimate partner physical and sexual violence on post-traumatic stress disorder symptomatology', *Violence and victims*, 18: 87-94.

Berardi, Andrea, Gustav Schelling, and Patrizia Campolongo. 2016. 'The endocannabinoid system and Post Traumatic Stress Disorder (PTSD): From preclinical findings to innovative therapeutic approaches in clinical settings', *Pharmacological research*, 111: 668-78.

Berninger, Amy, Mayris P Webber, Justin K Niles, Jackson Gustave, Roy Lee, Hillel W Cohen, Kerry Kelly, Malachy Corrigan, and David J Prezant. 2010. 'Longitudinal study of probable post‐traumatic stress disorder in firefighters exposed to the World Trade Center disaster', *American journal of industrial medicine*, 53: 1177-85.

Berton, Margaret Wright, and Sally D Stabb. 1996. 'Exposure to violence and post-traumatic stress disorder in urban adolescents', *Adolescence*, 31: 489.

Bezabh, Yodit Habtamu, Solomon Mekonnen Abebe, Tolesa Fanta, Agitu Tadese, and Mikiyas Tulu. 2018. 'Prevalence and associated factors of post-traumatic stress disorder among emergency responders of Addis Ababa Fire and Emergency Control and Prevention Service Authority, Ethiopia: institution-based, cross-sectional study', *BMJ open*, 8: e020705.

Bienvenu, O Joseph, Jonathan Gellar, Benjamin M Althouse, Elizabeth Colantuoni, Thiti Sricharoenchai, Pedro A Mendez-Tellez, Carl Shanholtz, Cheryl R Dennison, Peter J Pronovost, and Dale M Needham. 2013. 'Post-traumatic stress disorder symptoms after acute lung injury: a 2-year prospective longitudinal study', *Psychological medicine*, 43: 2657-71.

Bisson, Jonathan I. 2007. 'Post-traumatic stress disorder', *Bmj*, 334: 789-93.

Bisson, Jonathan I, and Martin P Deahl. 1994. 'Psychological debriefing and prevention of post-traumatic stress: More research is needed', *The British Journal of Psychiatry*, 165: 717-20.

Blanc, Judite, Eric Bui, Yoram Mouchenik, Daniel Derivois, and Philippe Birmes. 2015. 'Prevalence of post-traumatic stress disorder and depression in two groups of children one year after the January 2010 earthquake in Haiti', *Journal of affective disorders*, 172: 121-26.

Blanchard, Edward B, Edward J Hickling, Kristine A Barton, Ann E Taylor, Warren R Loos, and Jacqueline Jones-Alexander. 1996. 'One-year prospective follow-up of motor vehicle accident victims', *Behaviour research and therapy*, 34: 775-86.

Blanchard, Edward B, Edward J Hickling, Neil Mitnick, Ann E Taylor, Warren R Loos, and Todd C Buckley. 1995. 'The impact of severity of physical injury and perception of life threat in the development of post-traumatic stress disorder in motor vehicle accident victims', *Behaviour research and therapy*, 33: 529-34.

Blanchard, Edward B, Edward J Hickling, Ann E Taylor, Warren R Loos, Catherine A Forneris, and James Jaccard. 1996. 'Who develops PTSD from motor vehicle accidents?', *Behaviour research and therapy*, 34: 1-10.

Blanchard, Edward B, and Connie H Veazey. 2001. 'Mental disorders resulting from road traffic accidents', *Current Opinion in Psychiatry*, 14: 143-47.

Blaszczynski, Alex, Kylie Gordon, Derrick Silove, David Sloane, Ken Hillman, and Paula Panasetis. 1998. 'Psychiatric morbidity following motor vehicle accidents: a review of methodological issues', *Comprehensive psychiatry*, 39: 111-21.

Bockhop, Fabian, Katrin Cunitz, Marina Zeldovich, Anna Buchheim, Tim Beissbarth, York Hagmayer, and Nicole von Steinbuechel. 2023. 'Influence of sociodemographic, premorbid, and injury-related factors on post-traumatic stress, anxiety, and depression after traumatic brain injury', *Journal of clinical medicine*, 12: 3873.

Boeschen, Laura E, Mary P Koss, Aurelio Jose Figueredo, and James A Coan. 2001. 'Experiential avoidance and post-traumatic stress disorder: A cognitive mediational model of rape recovery', *Journal of Aggression, Maltreatment & Trauma*, 4: 211-45.

Bolton, Derek, Jonathan Hill, Dominic O'Ryan, Orlee Udwin, Stephanie Boyle, and William Yule. 2004. 'Long‐term effects of psychological trauma on psychosocial functioning', *Journal of child psychology and psychiatry*, 45: 1007-14.

Bolton, Derek, Dominic O'Ryan, Orlee Udwin, Stephanie Boyle, and William Yule. 2000. 'The long-term psychological effects of a disaster experienced in adolescence: II: General psychopathology', *The Journal of Child Psychology and Psychiatry and Allied Disciplines*, 41: 513-23.

Bolton, Elisa, Dana R Holohan, Lynda A King, and Daniel W King. 2004. 'Acute and post-traumatic stress disorder.' in, *Psychopathology in the workplace* (Routledge).

Bordbar, Mohammad Reza Fayyazi. 2007. 'POST TRAUMATIC STRESS DISORDER AND RELATED FACTORS FOLLOWING ORTHOPEDIC TRAUMAS', *Journal of Pakistan Psychiatric Society*, 4: 37-43.

Boris, Neil W, Alan C Ou, and Rohini Singh. 2005. 'Preventing post-traumatic stress disorder after mass exposure to violence', *Biosecurity and Bioterrorism: Biodefense Strategy, Practice, and Science*, 3: 154-63.

Boscarino, Joseph A. 1995. 'Post-traumatic stress and associated disorders among Vietnam veterans: The significance of combat exposure and social support', *Journal of Traumatic stress*, 8: 317-36.

Boumpa, Vasiliki, Aikaterini Papatoukaki, Anastasia Kourti, Sofia Mintzia, Eleni Panagouli, Flora Bacopoulou, Theodora Psaltopoulou, Chara Spiliopoulou, Maria Tsolia, and Theodoros N Sergentanis. 2024. 'Sexual abuse and post-traumatic stress disorder in childhood, adolescence and young adulthood: a systematic review and meta-analysis', *European child & adolescent psychiatry*, 33: 1653-73.

Bown, Dominic, Antonio Belli, Kasim Qureshi, David Davies, Emma Toman, and Rachel Upthegrove. 2019. 'Post-traumatic stress disorder and self-reported outcomes after traumatic brain injury in victims of assault', *PLoS One*, 14: e0211684.

Bracken, Patrick J. 2001. 'Post-modernity and post-traumatic stress disorder', *Social Science & Medicine*, 53: 733-43.

Brandes, Dalia, Gershon Ben-Schachar, Assaf Gilboa, Omer Bonne, Sara Freedman, and Arieh Y Shalev. 2002. 'PTSD symptoms and cognitive performance in recent trauma survivors', *Psychiatry research*, 110: 231-38.

Breen, Michael S, Adam X Maihofer, Stephen J Glatt, Daniel S Tylee, Sharon D Chandler, Ming T Tsuang, Victoria B Risbrough, Dewleen G Baker, Daniel T O'Connor, and Caroline M Nievergelt. 2015. 'Gene networks specific for innate immunity define post-traumatic stress disorder', *Molecular psychiatry*, 20: 1538-45.

Bremner, J Douglas. 2002. 'Neuroimaging studies in post-traumatic stress disorder', *Current psychiatry reports*, 4: 254-63.

. 2006. 'Traumatic stress: effects on the brain', *Dialogues in clinical neuroscience*, 8: 445-61.

Breslau, N, and L Schultz. 2013. 'Neuroticism and post-traumatic stress disorder: a prospective investigation', *Psychological medicine*, 43: 1697-702.

Breslau, Naomi, EL Peterson, LM Poisson, LR Schultz, and VC Lucia. 2004. 'Estimating post-traumatic stress disorder in the community: lifetime perspective and the impact of typical traumatic events', *Psychological medicine*, 34: 889-98.

Brewin, Chris R. 1998. 'Intrusive autobiographical memories in depression and post‐traumatic stress disorder', *Applied Cognitive Psychology: The Official Journal of the Society for Applied Research in Memory and Cognition*, 12: 359-70.

Brewin, Chris R, Bernice Andrews, and Suzanna Rose. 2000. 'Fear, helplessness, and horror in posttraumatic stress disorder: Investigating DSM‐IV Criterion A2 in victims of violent crime', *Journal of Traumatic Stress: Official Publication of The International Society for Traumatic Stress Studies*, 13: 499-509.

Brief, Debroad J, AR Bollinger, MJ Vielhauer, JA Berger-Greenstein, EE Morgan, SM Brady, L Mm Buondonno, TM Keane, Health Outcomes For The Hiv/aids Treatment Adherence, and Cost Study Group. 2004. 'Understanding the interface of HIV, trauma, post-traumatic stress disorder, and substance use and its implications for health outcomes', *AIDS care*, 16: 97-120.

Brijnath, Bianca, Samantha Bunzli, Ting Xia, Nabita Singh, Peter Schattner, Alex Collie, Michele Sterling, and Danielle Mazza. 2016. 'General practitioners knowledge and management of whiplash associated disorders and post-traumatic stress disorder: implications for patient care', *BMC family practice*, 17: 1-11.

Brom, Daniel, Rolf J Kleber, and Marijke C Hofman. 1993. 'Victims of traffic accidents: Incidence and prevention of post‐traumatic stress disorder', *Journal of clinical psychology*, 49: 131-40.

Bromet, Evelyn J. 2012. 'Mental health consequences of the Chernobyl disaster', *Journal of radiological protection*, 32: N71.

Bromet, Evelyn J, Lukoye Atwoli, Norito Kawakami, Fernando Navarro-Mateu, P Piotrowski, AJ King, Sergio Aguilar-Gaxiola, Jordi Alonso, Brentan Bunting, and Koen Demyttenaere. 2017. 'Post-traumatic stress disorder associated with natural and human-made disasters in the World Mental Health Surveys', *Psychological medicine*, 47: 227-41.

Bromet, Evelyn J, Michael J Hobbs, Sean AP Clouston, Adam Gonzalez, Roman Kotov, and Benjamin J Luft. 2016. 'DSM-IV post-traumatic stress disorder among World Trade Center responders 11–13 years after the disaster of 11 September 2001 (9/11)', *Psychological medicine*, 46: 771-83.

Brooks, Samantha, Richard Amlot, Gideon James Rubin, and Neil Greenberg. 2020. 'Psychological resilience and post-traumatic growth in disaster-exposed organisations: overview of the literature', *BMJ Mil Health*, 166: 52-56.

Brousse, Georges, Benjamin Arnaud, Jordane Durand Roger, Julie Geneste, Delphine Bourguet, Frederic Zaplana, Olivier Blanc, Jeannot Schmidt, and Louis Jehel. 2011. 'Management of traumatic events: Influence of emotion-centered coping strategies on the occurrence of dissociation and post-traumatic stress disorder', *Neuropsychiatric disease and treatment*: 127-33.

Brown, Adam D, Donna Rose Addis, Tracy A Romano, Charles R Marmar, Richard A Bryant, William Hirst, and Daniel L Schacter. 2014. 'Episodic and semantic components of autobiographical memories and imagined future events in post-traumatic stress disorder', *Memory*, 22: 595-604.

Brunetti, Marcella, Gianna Sepede, Gianluca Mingoia, Claudia Catani, Antonio Ferretti, Arcangelo Merla, Cosimo Del Gratta, Gian Luca Romani, and Claudio Babiloni. 2010. 'Elevated response of human amygdala to neutral stimuli in mild post traumatic stress disorder: neural correlates of generalized emotional response', *Neuroscience*, 168: 670-79.

Bryant, B, R Mayou, L Wiggs, Anke Ehlers, and G Stores. 2004. 'Psychological consequences of road traffic accidents for children and their mothers', *Psychological medicine*, 34: 335-46.

Bryant, RA. 1996. 'Predictors of post-traumatic stress disorder following burns injury', *Burns*, 22: 89-92.

Bryant, Richard. 2011. 'Post-traumatic stress disorder vs traumatic brain injury', *Dialogues in clinical neuroscience*, 13: 251-62.

Bryant, Richard A. 2019. 'Post‐traumatic stress disorder: a state‐of‐the‐art review of evidence and challenges', *World psychiatry*, 18: 259-69.

Bryant, Richard A, Mark Creamer, Meaghan O’donnell, Derrick Silove, C Richard Clark, and Alexander C McFarlane. 2009. 'Post-traumatic amnesia and the nature of post-traumatic stress disorder after mild traumatic brain injury', *Journal of the International Neuropsychological Society*, 15: 862-67.

Bryant, Richard A, Kim Felmingham, Andrew Kemp, Pritha Das, Gerard Hughes, A Peduto, and Leanne Williams. 2008. 'Amygdala and ventral anterior cingulate activation predicts treatment response to cognitive behaviour therapy for post-traumatic stress disorder', *Psychological medicine*, 38: 555-61.

Bryant, Richard A, and Allison G Harvey. 1995a. 'Avoidant coping style and post-traumatic stress following motor vehicle accidents', *Behaviour research and therapy*, 33: 631-35.

. 1995b. 'Processing threatening information in posttraumatic stress disorder', *Journal of abnormal psychology*, 104: 537.

Bryant, Richard A, Michelle L Moulds, and Reginald VD Nixon. 2003. 'Cognitive behaviour therapy of acute stress disorder: a four-year follow-up', *Behaviour research and therapy*, 41: 489-94.

Buckley, Todd C, Edward B Blanchard, and Edward J Hickling. 1998. 'A confirmatory factor analysis of posttraumatic stress symptoms', *Behaviour research and therapy*, 36: 1091-99.

Bui, E, A Brunet, B Olliac, E Very, C Allenou, J-P Raynaud, I Claudet, S Bourdet-Loubère, H Grandjean, and L Schmitt. 2011. 'Validation of the Peritraumatic Dissociative Experiences Questionnaire and Peritraumatic Distress Inventory in school-aged victims of road traffic accidents', *European Psychiatry*, 26: 108-11.

Burges, C, and TM McMillan. 2001. 'The ability of naive participants to report symptoms of post‐traumatic stress disorder', *British Journal of Clinical Psychology*, 40: 209-14.

Burrai, Jessica, Alessandro Quaglieri, Giulia Lausi, Angelo Fraschetti, Alessandro Couyoumdjian, Umberto Guidoni, Sandro Vedovi, Stefano Ferracuti, AM Giannini, and Emanuela Mari. 2021. 'A model of early psychological intervention for direct and indirect road victims', *Mediterranean Journal of Clinical Psychology*, 9.

Burstein, Allan. 1986. 'Treatment noncompliance in patients with post-traumatic stress disorder', *Psychosomatics*, 27: 37-40.

Busuttil, Walter. 2004. 'Presentations and management of post traumatic stress disorder and the elderly: a need for investigation', *International Journal of Geriatric Psychiatry*, 19: 429-39.

Butler, Dennis J, H Steven Moffic, and NICK W TURKAL. 1999. 'Post-traumatic stress reactions following motor vehicle accidents', *American Family Physician*, 60: 524-30.

Campanini, Rosaly FB, Aline F Schoedl, Mariana C Pupo, Ana Clara H Costa, Janice L Krupnick, and Marcelo F Mello. 2010. 'Efficacy of interpersonal therapy‐group format adapted to post‐traumatic stress disorder: an open‐label add‐on trial', *Depression and Anxiety*, 27: 72-77.

Candel, Ingrid, and Harald Merckelbach. 2004. 'Peritraumatic dissociation as a predictor of post-traumatic stress disorder: A critical review', *Comprehensive psychiatry*, 45: 44-50.

Canterbury, Rachel, and William Yule. 2021. 'The effects on children of road accidents.' in, *The Aftermath of Road Accidents* (Routledge).

Cantón-Cortés, David, and José Cantón. 2010. 'Coping with child sexual abuse among college students and post-traumatic stress disorder: The role of continuity of abuse and relationship with the perpetrator', *Child abuse & neglect*, 34: 496-506.

Cantor, Chris. 2009. 'Post-traumatic stress disorder: evolutionary perspectives', *Australian & New Zealand Journal of Psychiatry*, 43: 1038-48.

Cantor, Chris, and John Price. 2007. 'Traumatic entrapment, appeasement and complex post-traumatic stress disorder: Evolutionary perspectives of hostage reactions, domestic abuse and the Stockholm syndrome', *Australian & New Zealand Journal of Psychiatry*, 41: 377-84.

Carlier, Ingrid VE, Regina D Lamberts, Annephine J Van Uchelen, and Berthold PR Gersons. 1998. 'Disaster‐related post‐traumatic stress in police officers: A field study of the impact of debriefing', *Stress medicine*, 14: 143-48.

Carlier, Ingrid VE, AE Voerman, and Berthold PR Gersons. 2000. 'The influence of occupational debriefing on post‐traumatic stress symptomatology in traumatized police officers', *British Journal of Medical Psychology*, 73: 87-98.

Carmassi, CLAUDIA, Hagop S Akiskal, SS Yong, PAOLO Stratta, E Calderani, ENRICO Massimetti, Kareen K Akiskal, A Rossi, and LILIANA Dell'Osso. 2013. 'Post-traumatic stress disorder in DSM-5: estimates of prevalence and criteria comparison versus DSM-IV-TR in a non-clinical sample of earthquake survivors', *Journal of affective disorders*, 151: 843-48.

Carmassi, Claudia, Valerio Dell’Oste, Claudia Foghi, Carlo Antonio Bertelloni, Eugenia Conti, Sara Calderoni, Roberta Battini, and Liliana Dell’Osso. 2021. 'Post-traumatic stress reactions in caregivers of children and adolescents/young adults with severe diseases: A systematic review of risk and protective factors', *International journal of environmental research and public health*, 18: 189.

Carr, Alan. 2004. 'Interventions for post-traumatic stress disorder in children and adolescents', *Pediatric Rehabilitation*, 7: 231-44.

Cha, Hye Ji, and Sul Yeong Bang. 2019. 'Factors influencing post-traumatic growth in traffic accident patient', *Journal of the Korea Academia-Industrial Cooperation Society*, 20: 254-64.

Chen, Chwen-Cheng, Tzung-Lieh Yeh, Yen Kuang Yang, Shin-Jaw Chen, IH Lee, LS Fu, CY Yeh, HC Hsu, WL Tsai, and SH Cheng. 2001. 'Psychiatric morbidity and post-traumatic symptoms among survivors in the early stage following the 1999 earthquake in Taiwan', *Psychiatry research*, 105: 13-22.

Chen, Long, Hongzhuan Tan, Reuben Cofie, Shimin Hu, Yan Li, Jia Zhou, Tubao Yang, Xuemin Tang, Guanghui Cui, and Aizhong Liu. 2015. 'Prevalence and determinants of chronic post-traumatic stress disorder after floods', *Disaster medicine and public health preparedness*, 9: 504-08.

Chen, Shulin, Lingjiang Li, Baihua Xu, and Jun Liu. 2009. 'Insular cortex involvement in declarative memory deficits in patients with post-traumatic stress disorder', *BMC psychiatry*, 9: 1-9.

Chen, Yanfang, Xiaoxian Yang, Chentao Guo, Yan Liao, Lixing Guo, Wenjun Chen, Innie Chen, Daniel Krewski, Shi Wu Wen, and Ri-hua Xie. 2020. 'Prevalence of post-traumatic stress disorder following caesarean section: a systematic review and meta-analysis', *Journal of Women's Health*, 29: 200-09.

Chen, Ye-Fei, and Hu Zhao. 2019. 'Post-traumatic stress disorder: relationship to traumatic brain injury and approach to forensic psychiatry evaluation', *Journal of Forensic Science and Medicine*, 5: 33-39.

Chibnall, John T, and Paul N Duckro. 1994. 'Post‐traumatic stress disorder in chronic post‐traumatic headache patients', *Headache: The Journal of Head and Face Pain*, 34: 357-61.

Chin, Wei-Shan, Judith Shu-Chu Shiao, Shih-Cheng Liao, Chun-Ya Kuo, Chih-Chieh Chen, and Yue Leon Guo. 2017. 'Depressive, anxiety and post-traumatic stress disorders at six years after occupational injuries', *European archives of psychiatry and clinical neuroscience*, 267: 507-16.

Chossegros, Laetitia, Martine Hours, Pierrette Charnay, Marlène Bernard, Emmanuel Fort, Dominique Boisson, Pierre-Olivier Sancho, Sai Nan Yao, and Bernard Laumon. 2011. 'Predictive factors of chronic post-traumatic stress disorder 6 months after a road traffic accident', *Accident Analysis & Prevention*, 43: 471-77.

CHOU, FRANK HUANG‐CHIH, HUNG‐CHI WU, Pesus Chou, CHAO‐YUEH SU, KUAN‐YI TSAI, SHIN‐SHIN CHAO, MING‐CHAO CHEN, TOM TUNG‐PING SU, WEN‐JUNG SUN, and WEN‐CHEN OU‐YANG. 2007. 'Epidemiologic psychiatric studies on post‐disaster impact among Chi‐Chi earthquake survivors in Yu‐Chi, Taiwan', *Psychiatry and clinical neurosciences*, 61: 370-78.

Christiani, Ribka Wahyu, and Yohanes Yappo. 2023. 'Post-Traumatic Validative Incidentals Amongst Tertiary Students', *EDUJAVARE: International Journal of Educational Research*, 1: 93-107.

Chung, H, and Naomi Breslau. 2008. 'The latent structure of post-traumatic stress disorder: Tests of invariance by gender and trauma type', *Psychological medicine*, 38: 563-73.

Chung, Man Cheung, Julie Werrett, Yvette Easthope, and Steven Farmer. 2004. 'Coping with post‐traumatic stress: young, middle‐aged and elderly comparisons', *International Journal of Geriatric Psychiatry*, 19: 333-43.

Cipriano Jr, Robert Joseph. 2001. *Treatment of post traumatic stress disorder in Hurricane survivors: A program design* (Carlos Albizu University).

Clohessy, Sue, and Anke Ehlers. 1999. 'PTSD symptoms, response to intrusive memories and coping in ambulance service workers', *British Journal of Clinical Psychology*, 38: 251-65.

Cohen, Hagit, Alan Jotkowitz, Dan Buskila, Sharon Pelles-Avraham, Zeev Kaplan, Lily Neumann, and Ami D Sperber. 2006. 'Post-traumatic stress disorder and other co-morbidities in a sample population of patients with irritable bowel syndrome', *European journal of internal medicine*, 17: 567-71.

Cohen, Hagit, Lily Neumann, Yehoshua Haiman, Michael A Matar, Joseph Press, and Dan Buskila. 2002. "Prevalence of post-traumatic stress disorder in fibromyalgia patients: overlapping syndromes or post-traumatic fibromyalgia syndrome?" In *Seminars in arthritis and rheumatism*, 38-50. Elsevier.

Cohen, Judith A, and Michael S Scheeringa. 2009. 'Post-traumatic stress disorder diagnosis in children: challenges and promises', *Dialogues in clinical neuroscience*, 11: 91-99.

Collicutt McGrath, Joanna, and P Alex Linley. 2006. 'Post-traumatic growth in acquired brain injury: A preliminary small scale study', *Brain Injury*, 20: 767-73.

Colville, Gillian, and Christine Pierce. 2012. 'Patterns of post-traumatic stress symptoms in families after paediatric intensive care', *Intensive care medicine*, 38: 1523-31.

Connor, Kathryn M, and Jonathan RT Davidson. 2001. 'SPRINT: A brief global assessment of post-traumatic stress disorder', *International clinical psychopharmacology*, 16: 279-84.

Cordova, Matthew J, Michelle B Riba, and David Spiegel. 2017. 'Post-traumatic stress disorder and cancer', *The Lancet Psychiatry*, 4: 330-38.

Coronas, Ramón, Gemma García‐Parés, Carme Viladrich, Josep Manel Santos, and Josep Manel Menchón. 2008. 'Clinical and sociodemographic variables associated with the onset of posttraumatic stress disorder in road traffic accidents', *Depression and Anxiety*, 25: E16-E23.

Coughlin, Steven S. 2011. 'Post-traumatic stress disorder and cardiovascular disease', *The open cardiovascular medicine journal*, 5: 164.

Crabbe, JM, Douglas MG Bowley, KD Boffard, David Alan Alexander, and Susan Klein. 2004. 'Are health professionals getting caught in the crossfire? The personal implications of caring for trauma victims', *Emergency medicine journal*, 21: 568-72.

Creamer, Mark, P Burgess, and AC McFarlane. 2001. 'Post-traumatic stress disorder: findings from the Australian National Survey of Mental Health and Well-being', *Psychological medicine*, 31: 1237-47.

Creamer, Mark, and Meaghan O'Donnell. 2002. 'Post-traumatic stress disorder', *Current Opinion in Psychiatry*, 15: 163-68.

Crumlish, Niall, and Killian O'Rourke. 2010. 'A systematic review of treatments for post-traumatic stress disorder among refugees and asylum-seekers', *The Journal of Nervous and Mental Disease*, 198: 237-51.

Curle, CE, and C Williams. 1996. 'Post‐traumatic stress reactions in children: Gender differences in the incidence of trauma reactions at two years and examination of factors influencing adjustment', *British Journal of Clinical Psychology*, 35: 297-309.

Cuthbertson, Brian H, Alastair Hull, Mary Strachan, and Judith Scott. 2004. 'Post-traumatic stress disorder after critical illness requiring general intensive care', *Intensive care medicine*, 30: 450-55.

Cyniak-Cieciura, Maria, and Bogdan Zawadzki. 2021. 'The relationship between temperament traits and post-traumatic stress disorder symptoms and its moderators: Meta-analysis and meta-regression', *Trauma, Violence, & Abuse*, 22: 702-16.

Czarnocka, Jo, and Pauline Slade. 2000. 'Prevalence and predictors of post‐traumatic stress symptoms following childbirth', *British Journal of Clinical Psychology*, 39: 35-51.

D'Souza, D. 1995. 'POST‐TRAUMATIC STRESS DISORDER—A SCAR FOR LIFE', *International Journal of Clinical Practice*, 49: 309-13.

Dai, Wenjie, Long Chen, Zhiwei Lai, Yan Li, Jieru Wang, and Aizhong Liu. 2016. 'The incidence of post-traumatic stress disorder among survivors after earthquakes: a systematic review and meta-analysis', *BMC psychiatry*, 16: 1-11.

Dai, Wenjie, Long Chen, Hongzhuan Tan, Jieru Wang, Zhiwei Lai, Atipatsa C Kaminga, Yan Li, and Aizhong Liu. 2016. 'Association between social support and recovery from post-traumatic stress disorder after flood: a 13–14 year follow-up study in Hunan, China', *BMC public health*, 16: 1-9.

Dai, Wenjie, Aizhong Liu, Atipatsa C Kaminga, Jing Deng, Zhiwei Lai, Jianzhou Yang, and Shi Wu Wen. 2018. 'Prevalence of acute stress disorder among road traffic accident survivors: a meta-analysis', *BMC psychiatry*, 18: 1-11.

Danielsson, FB, M Schultz Larsen, B Nørgaard, and JM Lauritsen. 2018. 'Quality of life and level of post-traumatic stress disorder among trauma patients: A comparative study between a regional and a university hospital', *Scandinavian journal of trauma, resuscitation and emergency medicine*, 26: 1-9.

Dansky, Bonnie S, Christina A Byrne, and Kathleen T Brady. 1999. 'Intimate violence and post-traumatic stress disorder among individuals with cocaine dependence', *The American Journal of Drug and Alcohol Abuse*, 25: 257-68.

Daskalakis, Nikolaos P, Amy Lehrner, and Rachel Yehuda. 2013. 'Endocrine aspects of post-traumatic stress disorder and implications for diagnosis and treatment', *Endocrinology and Metabolism Clinics*, 42: 503-13.

Daud, Atia, Erling Skoglund, and Per‐Anders Rydelius. 2005. 'Children in families of torture victims: Transgenerational transmission of parents’ traumatic experiences to their children', *International Journal of Social Welfare*, 14: 23-32.

Davidson, Jonathan RT, SW Book, JT Colket, LA Tupler, S Roth, D David, M Hertzberg, T Mellman, JC Beckham, and RD Smith. 1997. 'Assessment of a new self-rating scale for post-traumatic stress disorder', *Psychological medicine*, 27: 153-60.

Davidson, Jonathan RT, Dana Hughes, Dana G Blazer, and Linda K George. 1991. 'Post-traumatic stress disorder in the community: an epidemiological study', *Psychological medicine*, 21: 713-21.

Davidson, Jonathan, and Rebecca Smith. 1990. 'Traumatic experiences in psychiatric outpatients', *Journal of Traumatic stress*, 3: 459-75.

Davidson, JRT. 2000. 'Trauma: the impact of post-traumatic stress disorder', *Journal of Psychopharmacology*, 14: S5-S12.

Davis, Glenn Craig, and Naomi Breslau. 1994. 'Post-traumatic stress disorder in victims of civilian trauma and criminal violence', *Psychiatric Clinics*, 17: 289-99.

Davydow, Dimitry S, Jeneen M Gifford, Sanjay V Desai, Dale M Needham, and O Joseph Bienvenu. 2008. 'Posttraumatic stress disorder in general intensive care unit survivors: a systematic review', *General hospital psychiatry*, 30: 421-34.

De Bellis, Michael D, and Lisa A Thomas. 2003. 'Biologic findings of post-traumatic stress disorder and child maltreatment', *Current psychiatry reports*, 5: 108-17.

De Bellis, Michael D, and Thomas Van Dillen. 2005. 'Childhood post-traumatic stress disorder: An overview', *Child and Adolescent Psychiatric Clinics*, 14: 745-72.

De Boer, Jacoba Coby, Anja Lok, Ellen van’t Verlaat, Hugo J Duivenvoorden, Arnold B Bakker, and Bert J Smit. 2011. 'Work-related critical incidents in hospital-based health care providers and the risk of post-traumatic stress symptoms, anxiety, and depression: a meta-analysis', *Social Science & Medicine*, 73: 316-26.

Dehghan-Nayeri, Nahid, Hooman Shahsavari, Shahrokh Yousefzadeh-Chabok, and Hamid Haghani. 2017. 'Psycho-social and mental variables and post-traumatic stress disorder in traffic accident survivors in northern Iran', *Bulletin of Emergency & Trauma*, 5: 197.

Deja, Maria, Claudia Denke, Steffen Weber-Carstens, Jürgen Schröder, Christian E Pille, Frank Hokema, Konrad J Falke, and Udo Kaisers. 2006. 'Social support during intensive care unit stay might improve mental impairment and consequently health-related quality of life in survivors of severe acute respiratory distress syndrome', *Critical care*, 10: 1-12.

Dekel, Rachel, and Candice M Monson. 2010. 'Military-related post-traumatic stress disorder and family relations: Current knowledge and future directions', *Aggression and violent behavior*, 15: 303-09.

Delahanty, Douglas L, Holly B Herberman, Karrie J Craig, Michele C Hayward, Carol S Fullerton, Robert J Ursano, and Andrew Baum. 1997. 'Acute and chronic distress and posstraumatic stress disorder as a function of responsibility for serious motor vehicle accidents', *Journal of Consulting and Clinical Psychology*, 65: 560.

Delahanty, Douglas L, A Jay Raimonde, Eileen Spoonster, and Michael Cullado. 2003. 'Injury severity, prior trauma history, urinary cortisol levels, and acute PTSD in motor vehicle accident victims', *Journal of anxiety disorders*, 17: 149-64.

Dell’Osso, Liliana, Paolo Stratta, Ciro Conversano, Enrico Massimetti, Kareen K Akiskal, Hagop S Akiskal, Alessandro Rossi, and Claudia Carmassi. 2014. 'Lifetime mania is related to post-traumatic stress symptoms in high school students exposed to the 2009 L’Aquila earthquake', *Comprehensive psychiatry*, 55: 357-62.

Deng, Lisa X, Abigail May Khan, David Drajpuch, Stephanie Fuller, Jonathan Ludmir, Christopher E Mascio, Sara L Partington, Ayesha Qadeer, Lynda Tobin, and Adrienne H Kovacs. 2016. 'Prevalence and correlates of post-traumatic stress disorder in adults with congenital heart disease', *The American journal of cardiology*, 117: 853-57.

Derivois, Daniel, Jude Mary Cénat, Nephtalie Eva Joseph, Amira Karray, and Khadija Chahraoui. 2017. 'Prevalence and determinants of post-traumatic stress disorder, anxiety and depression symptoms in street children survivors of the 2010 earthquake in Haiti, four years after', *Child abuse & neglect*, 67: 174-81.

Descilo, Teresa, A Vedamurtachar, PL Gerbarg, D Nagaraja, BN Gangadhar, B Damodaran, B Adelson, LH Braslow, S Marcus, and RP Brown. 2010. 'Effects of a yoga breath intervention alone and in combination with an exposure therapy for post‐traumatic stress disorder and depression in survivors of the 2004 South‐East Asia tsunami', *Acta Psychiatrica Scandinavica*, 121: 289-300.

Dewar, Michelle, Alison Paradis, and Christophe A Fortin. 2020. 'Identifying trajectories and predictors of response to psychotherapy for post-traumatic stress disorder in adults: A systematic review of literature', *The Canadian Journal of Psychiatry*, 65: 71-86.

Difede, Joann, and Hunter G Hoffman. 2002. 'Virtual reality exposure therapy for World Trade Center post-traumatic stress disorder: A case report', *Cyberpsychology & behavior*, 5: 529-35.

Dinnen, Stephanie, Vanessa Simiola, and Joan M Cook. 2015. 'Post-traumatic stress disorder in older adults: A systematic review of the psychotherapy treatment literature', *Aging & mental health*, 19: 144-50.

Djelantik, AAA Manik J, Geert E Smid, Rolf J Kleber, and Paul A Boelen. 2017. 'Symptoms of prolonged grief, post-traumatic stress, and depression after loss in a Dutch community sample: A latent class analysis', *Psychiatry research*, 247: 276-81.

Dobie, Dorcas J, Daniel R Kivlahan, Charles Maynard, Kristen R Bush, Miles McFall, Amee J Epler, and Katharine A Bradley. 2002. 'Screening for post-traumatic stress disorder in female Veteran’s Affairs patients: validation of the PTSD checklist', *General hospital psychiatry*, 24: 367-74.

Dörfel, Denise, Sirko Rabe, and Anke Karl*. 2008. 'Coping strategies in daily life as protective and risk factors for post traumatic stress in motor vehicle accident survivors', *Journal of Loss and trauma*, 13: 422-40.

Douglas, J. 1999. "Alterations in brain structure and function associated with post-traumatic stress disorder." In *Seminars in clinical neuropsychiatry*, 249-55.

Doustahadi, Amin, Amir Mohammad Beigee, Akbar Zare-Kaseb, and Mohammad Javad Ghazanfari. 2023. 'Suicidality after burn injuries: a significant overlooked challenge in burns survivors', *Journal of nursing reports in clinical practice*, 1: 104-05.

Du, Jun, Huapeng Diao, Xiaojuan Zhou, Chunkui Zhang, Yifei Chen, Yan Gao, and Yizheng Wang. 2022. 'Post-traumatic stress disorder: a psychiatric disorder requiring urgent attention', *Medical review*, 2: 219-43.

Duan, Wenjie, Pengfei Guo, and Pei Gan. 2015. 'Relationships among trait resilience, virtues, post-traumatic stress disorder, and post-traumatic growth', *PLoS One*, 10: e0125707.

Dückers, Michel LA, Eva Alisic, and Chris R Brewin. 2016. 'A vulnerability paradox in the cross-national prevalence of post-traumatic stress disorder', *The British Journal of Psychiatry*, 209: 300-05.

Duffy, Michael, Kate Gillespie, and David M Clark. 2007. 'Post-traumatic stress disorder in the context of terrorism and other civil conflict in Northern Ireland: randomised controlled trial', *Bmj*, 334: 1147.

Dunmore, Emma, David M Clark, and Anke Ehlers. 1997. 'Cognitive factors in persistent versus recovered post-traumatic stress disorder after physical or sexual assault: A pilot study', *Behavioural and Cognitive Psychotherapy*, 25: 147-59.

Dyb, Grete, Tine K Jensen, Egil Nygaard, Øivind Ekeberg, Trond H Diseths, Tore Wentzel-Larsen, and Siri Thoresen. 2014. 'Post-traumatic stress reactions in survivors of the 2011 massacre on Utøya Island, Norway', *The British Journal of Psychiatry*, 204: 361-67.

Dyregrov, Kari, DAG Nordanger, and Atle Dyregrov. 2003. 'Predictors of psychosocial distress after suicide, SIDS and accidents', *Death studies*, 27: 143-65.

Ecrepont, Adrien, Corentin Haidon, Benoit Girard, and Bob-Antoine J Menelas. 2016. "A fully customizable truck-driving simulator for the care of people suffering from Post-Traumatic Stress Disorder." In *2016 IEEE International Conference on Serious Games and Applications for Health (SeGAH)*, 1-8. IEEE.

Edmondson, Donald, and Roland von Känel. 2017. 'Post-traumatic stress disorder and cardiovascular disease', *The Lancet Psychiatry*, 4: 320-29.

Egan, Sarah J, Mary Hattaway, and Robert T Kane. 2014. 'The relationship between perfectionism and rumination in post traumatic stress disorder', *Behavioural and Cognitive Psychotherapy*, 42: 211-23.

Ehde, Dawn M, David R Patterson, Shelley A Wiechman, and Lawrence G Wilson. 2000. 'Post-traumatic stress symptoms and distress 1 year after burn injury', *The Journal of Burn Care & Rehabilitation*, 21: 105-11.

Ehlers, Anke, Richard A Mayou, and Bridget Bryant. 2003. 'Cognitive predictors of posttraumatic stress disorder in children: Results of a prospective longitudinal study', *Behaviour research and therapy*, 41: 1-10.

Ehring, Thomas, Anke Ehlers, and Edward Glucksman. 2006. 'Contribution of cognitive factors to the prediction of post-traumatic stress disorder, phobia and depression after motor vehicle accidents', *Behaviour research and therapy*, 44: 1699-716.

. 2008. 'Do cognitive models help in predicting the severity of posttraumatic stress disorder, phobia, and depression after motor vehicle accidents? A prospective longitudinal study', *Journal of Consulting and Clinical Psychology*, 76: 219.

Eid, Jarle, Julian F Thayer, and BJØRN H Johnsen. 1999. 'Measuring post‐traumatic stress: A psychometric evaluation of symptom‐and coping questionnaires based on a Norwegian sample', *Scandinavian journal of psychology*, 40: 101-08.

Eisenbruch, Maurice. 1991. 'From post-traumatic stress disorder to cultural bereavement: Diagnosis of Southeast Asian refugees', *Social Science & Medicine*, 33: 673-80.

El Khoury-Malhame, Myriam, Laura Lanteaume, Eva Maria Beetz, Jacques Roques, Emmanuelle Reynaud, Jean-Claude Samuelian, Olivier Blin, René Garcia, and Stephanie Khalfa. 2011. 'Attentional bias in post-traumatic stress disorder diminishes after symptom amelioration', *Behaviour research and therapy*, 49: 796-801.

Elder, Gregory A, Nathan P Dorr, Rita De Gasperi, Miguel A Gama Sosa, Michael C Shaughness, Eric Maudlin-Jeronimo, Aaron A Hall, Richard M McCarron, and Stephen T Ahlers. 2012. 'Blast exposure induces post-traumatic stress disorder-related traits in a rat model of mild traumatic brain injury', *Journal of neurotrauma*, 29: 2564-75.

Elklit, Ask, and Ole Brink. 2004. 'Acute stress disorder as a predictor of post-traumatic stress disorder in physical assault victims', *Journal of Interpersonal Violence*, 19: 709-26.

Elsesser, Karin, and Gudrun Sartory. 2007. 'Memory performance and dysfunctional cognitions in recent trauma victims and patients with post‐traumatic stress disorder', *Clinical Psychology & Psychotherapy: An International Journal of Theory & Practice*, 14: 464-74.

Emilien, C. Penasse, G. Charles, D. Martin, L. Lasseaux, A. Waltregny, G. 2000. 'Post-traumatic stress disorder: Hypotheses from clinical neuropsychology and psychopharmacology research', *International journal of psychiatry in clinical practice*, 4: 3-18.

Engelhard, Iris M, Marcel A van den Hout, and Merel Kindt. 2003. 'The relationship between neuroticism, pre-traumatic stress, and post-traumatic stress: A prospective study', *Personality and Individual Differences*, 35: 381-88.

Eriksson, Nils-Gustaf, and Tom Lundin. 1996. 'Early traumatic stress reactions among Swedish survivors of the m/s Estonia disaster', *The British Journal of Psychiatry*, 169: 713-16.

Famularo, Richard, Terence Fenton, Robert Kinscherff, and Marilyn Augustyn. 1996. 'Psychiatric comorbidity in childhood post traumatic stress disorder', *Child abuse & neglect*, 20: 953-61.

Fan, F, K Long, Y Zhou, Y Zheng, and Xijuan Liu. 2015. 'Longitudinal trajectories of post-traumatic stress disorder symptoms among adolescents after the Wenchuan earthquake in China', *Psychological medicine*, 45: 2885-96.

Fani, Negar, Tricia Z King, Tanja Jovanovic, Ebony M Glover, Bekh Bradley, KiSueng Choi, Timothy Ely, David A Gutman, and Kerry J Ressler. 2012. 'White matter integrity in highly traumatized adults with and without post-traumatic stress disorder', *Neuropsychopharmacology*, 37: 2740-46.

Farley, Lisa M, David R DeMaso, Eugene D’Angelo, Carolyn Kinnamon, Heather Bastardi, Clara E Hill, Elizabeth D Blume, and Deirdre E Logan. 2007. 'Parenting stress and parental post-traumatic stress disorder in families after pediatric heart transplantation', *The Journal of heart and lung transplantation*, 26: 120-26.

Farooqui, Mudassir, Syed A Quadri, Sajid S Suriya, Muhammad Adnan Khan, Muhammad Ovais, Zohaib Sohail, Samra Shoaib, Hassaan Tohid, and Muhammad Hassan. 2017. 'Posttraumatic stress disorder: a serious post-earthquake complication', *Trends in psychiatry and psychotherapy*, 39: 135-43.

Fecteau, Gary, and Richard Nicki. 1999. 'Cognitive behavioural treatment of post traumatic stress disorder after motor vehicle accident', *Behavioural and Cognitive Psychotherapy*, 27: 201-14.

Fecteau, Gary William. 2001. *Treatment of post-traumatic stress reactions to traffic accidents* (National Library of Canada= Bibliothèque nationale du Canada, Ottawa).

Feinstein, Anthony, and Ray Dolan. 1991. 'Predictors of post-traumatic stress disorder following physical trauma: an examination of the stressor criterion', *Psychological medicine*, 21: 85-91.

Feki, Rim, Lobna Zouari, Yosra Majdoub, Sana Omri, Imen Gassara, Najeh Smaoui, Manel Maalej Bouali, Ben Thabet, Nada Charfi, and Mohamed Maalej. 2024. 'Prevalence and predictors of post-traumatic stress disorder in road traffic accidents', *The Pan African Medical Journal*, 47: 89-89.

Feldner, Matthew T, Kimberly A Babson, and Michael J Zvolensky. 2007. 'Smoking, traumatic event exposure, and post-traumatic stress: A critical review of the empirical literature', *Clinical psychology review*, 27: 14-45.

Fernandez, Isabel. 2007. 'EMDR as treatment of post-traumatic reactions: A field study on child victims of an earthquake', *Educational and Child Psychology*, 24: 65.

Ferree, Nikole K, Malinda Wheeler, and Larry Cahill. 2012. 'The influence of emergency contraception on post-traumatic stress symptoms following sexual assault', *Journal of forensic nursing*, 8: 122-30.

Fichera, Giuseppe P, A Fattori, L Neri, Marina Musti, Maurizio Coggiola, and Guiliana Costa. 2015. 'Post-traumatic stress disorder among bank employee victims of robbery', *Occupational medicine*, 65: 283-89.

Fichtenberg, Norman L, Scott R Millis, Nancy R Mann, Ross D Zafonte, and Anna E Millard. 2000. 'Factors associated with insomnia among post-acute traumatic brain injury survivors', *Brain Injury*, 14: 659-67.

Figley, Charles R. 1988. 'A five-phase treatment of post-traumatic stress disorder in families', *Journal of Traumatic stress*, 1: 127-41.

Fischer, Susanne, Tabea Schumacher, Christine Knaevelsrud, Ulrike Ehlert, and Sarah Schumacher. 2021. 'Genes and hormones of the hypothalamic–pituitary–adrenal axis in post-traumatic stress disorder. What is their role in symptom expression and treatment response?', *Journal of Neural Transmission*, 128: 1279-86.

Fishbain, David A, Aditya Pulikal, John E Lewis, and Jinrun Gao. 2017. 'Chronic pain types differ in their reported prevalence of post-traumatic stress disorder (PTSD) and there is consistent evidence that chronic pain is associated with PTSD: an evidence-based structured systematic review', *Pain medicine*, 18: 711-35.

Fitzharris, Michael, Brian Fildes, and Judith Charlton. 2006. "Anxiety, acute-and post-traumatic stress symptoms following involvement in traffic crashes." In *Annual Proceedings/Association for the Advancement of Automotive Medicine*, 297. Association for the Advancement of Automotive Medicine.

Flatten, Guido, Dieter Wälte, and Volker Perlitz. 2008. 'Self-efficacy in acutely traumatized patients and the risk of developing a posttraumatic stress syndrome', *GMS Psycho-Social Medicine*, 5.

Flores, Elaine C, Andres M Carnero, and Angela M Bayer. 2014. 'Social capital and chronic post-traumatic stress disorder among survivors of the 2007 earthquake in Pisco, Peru', *Social Science & Medicine*, 101: 9-17.

Foa, Edna B, and David S Riggs. 1995. 'Posttraumatic stress disorder following assault: Theoretical considerations and empirical findings', *Current directions in psychological science*, 4: 61-65.

Foa, Edna B, Richard Zinbarg, and Barbara O Rothbaum. 1992. 'Uncontrollability and unpredictability in post-traumatic stress disorder: an animal model', *Psychological bulletin*, 112: 218.

Foley, Jim, Alex Hassett, and Emma Williams. 2022. '‘Getting on with the job’: A systematised literature review of secondary trauma and post-traumatic stress disorder (PTSD) in policing within the United Kingdom (UK)', *The Police Journal*, 95: 224-52.

Forbes, David, Mark Creamer, Andrea Phelps, Richard Bryant, Alexander McFarlane, Grant J Devilly, Lynda Matthews, Beverley Raphael, Chris Doran, and Tracy Merlin. 2007. 'Australian guidelines for the treatment of adults with acute stress disorder and post-traumatic stress disorder', *Australian & New Zealand Journal of Psychiatry*, 41: 637-48.

Foreman, Elaine Iljon, and Alexandra Hough. 1995. 'Post-traumatic stress disorder', *Physiotherapy in Mental Health*: 341-66.

Forneris, Catherine A, Gerald Gartlehner, Kimberly A Brownley, Bradley N Gaynes, Jeffrey Sonis, Emmanuel Coker-Schwimmer, Daniel E Jonas, Amy Greenblatt, Tania M Wilkins, and Carol L Woodell. 2013. 'Interventions to prevent post-traumatic stress disorder: a systematic review', *American journal of preventive medicine*, 44: 635-50.

Forte, Giuseppe, Francesca Favieri, Renata Tambelli, and Maria Casagrande. 2020. 'COVID-19 pandemic in the Italian population: validation of a post-traumatic stress disorder questionnaire and prevalence of PTSD symptomatology', *International journal of environmental research and public health*, 17: 4151.

Freedman, Sara A, Dalia Brandes, Tuvia Peri, and Arieh Y Shalev. 1999. 'Predictors of chronic post-traumatic stress disorder: A prospective study', *The British Journal of Psychiatry*, 174: 353-59.

Freeman, Daniel, C Thompson, Natasha Vorontsova, G Dunn, L-A Carter, P Garety, E Kuipers, Mel Slater, Angus Antley, and Ed Glucksman. 2013. 'Paranoia and post-traumatic stress disorder in the months after a physical assault: a longitudinal study examining shared and differential predictors', *Psychological medicine*, 43: 2673-84.

Friedman, Matthew J, Paula P Schnurr, and Annmarie McDonagh-Coyle. 1994. 'Post-traumatic stress disorder in the military veteran', *Psychiatric Clinics*, 17: 265-77.

Fu, Steven S, Miles McFall, Andrew J Saxon, Jean C Beckham, Timothy P Carmody, Dewleen G Baker, and Anne M Joseph. 2007. 'Post-traumatic stress disorder and smoking: a systematic review', *Nicotine & tobacco research*, 9: 1071-84.

Fuglsang, Annette Kjaer, Hanspeter Moergeli, and Ulrich Schnyder. 2004. 'Does acute stress disorder predict post-traumatic stress disorder in traffic accident victims? Analysis of a self-report inventory', *Nordic journal of psychiatry*, 58: 223-29.

Fung, Hong Wang, Wai Tong Chien, Henry Wai-Hang Ling, Colin A Ross, and Stanley Kam Ki Lam. 2022. 'The mediating role of post-traumatic stress disorder symptoms in the relationship between childhood adversities and depressive symptoms in two samples', *Child abuse & neglect*, 131: 105707.

Gabriel, Rafael, Laura Ferrando, Enrique Sainz Cortón, Carlos Mingote, Eduardo García-Camba, Alberto Fernández Liria, and Sandro Galea. 2007. 'Psychopathological consequences after a terrorist attack: An epidemiological study among victims, the general population, and police officers☆', *European Psychiatry*, 22: 339-46.

Galea, Sandro, Arijit Nandi, and David Vlahov. 2005. 'The epidemiology of post-traumatic stress disorder after disasters', *Epidemiologic reviews*, 27: 78-91.

Galea, Sandro, David Vlahov, Heidi Resnick, Jennifer Ahern, Ezra Susser, Joel Gold, Michael Bucuvalas, and Dean Kilpatrick. 2003. 'Trends of probable post-traumatic stress disorder in New York City after the September 11 terrorist attacks', *American journal of epidemiology*, 158: 514-24.

Gallo, Alain Dl, Joanne Barton, and William Li Parry-Jones. 1997. 'Road traffic accidents: early psychological consequences in children and adolescents', *The British Journal of Psychiatry*, 170: 358-62.

Gamper, Gunnar, Matthaeus Willeit, Fritz Sterz, Harald Herkner, Alexander Zoufaly, Kurt Hornik, Christof Havel, and AN Laggner. 2004. 'Life after death: posttraumatic stress disorder in survivors of cardiac arrest—prevalence, associated factors, and the influence of sedation and analgesia', *Critical care medicine*, 32: 378-83.

Gander, Marie-Louise, and Roland von Känel. 2006. 'Myocardial infarction and post-traumatic stress disorder: frequency, outcome, and atherosclerotic mechanisms', *European Journal of Preventive Cardiology*, 13: 165-72.

Garrison, Carol Z, Martin W Weinrich, Sally B Hardin, Sally Weinrich, and Lixia Wang. 1993. 'Post-traumatic stress disorder in adolescents after a hurricane', *American journal of epidemiology*, 138: 522-30.

Gavranidou, Maria, and Rita Rosner. 2003. 'The weaker sex? Gender and post‐traumatic stress disorder', *Depression and Anxiety*, 17: 130-39.

Geng, Fulei, Fang Fan, Lei Mo, Ian Simandl, and Xianchen Liu. 2013. 'Sleep problems among adolescent survivors following the 2008 Wenchuan earthquake in China: a cohort study', *The Journal of clinical psychiatry*, 74: 19721.

Gersons, Berthold PR, and Ingrid VE Carlier. 1992. 'Post-traumatic stress disorder: The history of a recent concept', *The British Journal of Psychiatry*, 161: 742-48.

Ghisi, Marta, Caterina Novara, Giulia Buodo, Matthew O Kimble, Simona Scozzari, Arianna Di Natale, Ezio Sanavio, and Daniela Palomba. 2013. 'Psychological distress and post-traumatic symptoms following occupational accidents', *Behavioral sciences*, 3: 587-600.

Giannopoulou, Ioanna, Marios Strouthos, Patrick Smith, Anastasia Dikaiakou, Vasiliki Galanopoulou, and William Yule. 2006. 'Post-traumatic stress reactions of children and adolescents exposed to the Athens 1999 earthquake', *European Psychiatry*, 21: 160-66.

Gillespie, Kate, Michael Duffy, Ann Hackmann, and David M Clark. 2002. 'Community based cognitive therapy in the treatment of post-traumatic stress disorder following the Omagh bomb', *Behaviour research and therapy*, 40: 345-57.

Gillies, Donna, Fiona Taylor, Carl Gray, Louise O'Brien, and Natalie d'Abrew. 2013. 'Psychological therapies for the treatment of post‐traumatic stress disorder in children and adolescents', *Evidence‐based child health: a Cochrane review journal*, 8: 1004-116.

Gilpin, NW, and JL Weiner. 2017. 'Neurobiology of comorbid post‐traumatic stress disorder and alcohol‐use disorder', *Genes, Brain and Behavior*, 16: 15-43.

Ginzburg, Karni, Cheryl Koopman, Lisa D Butler, Oxana Palesh, Helena C Kraemer, Catherine C Classen, and David Spiegel. 2006. 'Evidence for a dissociative subtype of post-traumatic stress disorder among help-seeking childhood sexual abuse survivors', *Journal of Trauma & Dissociation*, 7: 7-27.

Girard, Timothy D, Ayumi K Shintani, James C Jackson, Sharon M Gordon, Brenda T Pun, Melinda S Henderson, Robert S Dittus, Gordon R Bernard, and EWesley Ely. 2007. 'Risk factors for post-traumatic stress disorder symptoms following critical illness requiring mechanical ventilation: a prospective cohort study', *Critical care*, 11: 1-8.

Glaesmer, Heide, Thomas Gunzelmann, Elmar Braehler, Simon Forstmeier, and Andreas Maercker. 2010. 'Traumatic experiences and post-traumatic stress disorder among elderly Germans: results of a representative population-based survey', *International Psychogeriatrics*, 22: 661-70.

Glynn, Shirley M, Joan R Asarnow, Robert Asarnow, Vivek Shetty, Karin Elliot-Brown, Edward Black, and Thomas R Belin. 2003. 'The development of acute post-traumatic stress disorder after orofacial injury: a prospective study in a large urban hospital', *Journal of Oral and Maxillofacial Surgery*, 61: 785-92.

Goenjian, Armen K, Louis M Najarian, Robert S Pynoos, Alan M Steinberg, Gagik Manoukian, Anahid Tavosian, and Lynn A Fairbanks. 1994. 'Posttraumatic stress disorder in elderly and younger adults after the 1988 earthquake in Armenia', *American Journal of Psychiatry*, 151: 895-901.

Goldfinger, Judith Z, Donald Edmondson, Ian M Kronish, Kezhen Fei, Revathi Balakrishnan, Stanley Tuhrim, and Carol R Horowitz. 2014. 'Correlates of post-traumatic stress disorder in stroke survivors', *Journal of Stroke and Cerebrovascular Diseases*, 23: 1099-105.

Golier, Julia, and Rachel Yehuda. 2002. 'Neuropsychological processes in post-traumatic stress disorder', *Psychiatric Clinics*, 25: 295-315.

Gorst-Unsworth, Caroline, and Eva Goldenberg. 1998. 'Psychological sequelae of torture and organised violence suffered by refugees from Iraq: Trauma-related factors compared with social factors in exile', *The British Journal of Psychiatry*, 172: 90-94.

Goulston, Mark. 2012. *Post-traumatic stress disorder for dummies* (John Wiley & Sons).

Green, Bonnie L. 1993. 'Identifying survivors at risk: Trauma and stressors across events', *International handbook of traumatic stress syndromes*: 135-44.

. 1994. 'Psychosocial research in traumatic stress: An update', *Journal of Traumatic stress*, 7: 341-62.

Green, Bonnie L, Mary C Grace, Marshall G Vary, Teresa L Kramer, Goldine C Gleser, and Anthony C Leonard. 1994. 'Children of disaster in the second decade: A 17-year follow-up of Buffalo Creek survivors', *Journal of the American Academy of Child & Adolescent Psychiatry*, 33: 71-79.

Green, Bonnie L, and Jacob D Lindy. 1994. 'Post-traumatic stress disorder in victims of disasters', *Psychiatric Clinics*, 17: 301-09.

Green, Bonnie L, John P Wilson, and Jacob D Lindy. 2013. 'Conceptualizing post-traumatic stress disorder: A psychosocial framework.' in, *Trauma and its wake* (Routledge).

Green, Margaret M, Alexander C McFarlane, Christine E Hunter, and William M Griggs. 1993. 'Undiagnosed post‐traumatic stress disorder following motor vehicle accidents', *Medical journal of Australia*, 159: 529-34.

Green, Monica A, and Meryl A Berlin. 1987. 'Five psychosocial variables related to the existence of post‐traumatic stress disorder symptoms', *Journal of clinical psychology*, 43: 643-49.

Greenberg, Neil, Samantha Brooks, and Rebecca Dunn. 2015. 'Latest developments in post-traumatic stress disorder: diagnosis and treatment', *British Medical Bulletin*, 114: 147-55.

Greenspan, Arlene I, Anthony Y Stringer, VL Phillips, Flora M Hammond, and Felicia C Goldstein. 2006. 'Symptoms of post-traumatic stress: intrusion and avoidance 6 and 12 months after TBI', *Brain Injury*, 20: 733-42.

Griffiths, John, Gillian Fortune, Vicki Barber, and J Duncan Young. 2007. 'The prevalence of post traumatic stress disorder in survivors of ICU treatment: a systematic review', *Intensive care medicine*, 33: 1506-18.

Grinage, Bradley D. 2003. 'Diagnosis and management of post-traumatic stress disorder', *American Family Physician*, 68: 2401-09.

Groome, David, and Anastasia Soureti. 2004. 'Post‐traumatic stress disorder and anxiety symptoms in children exposed to the 1999 Greek earthquake', *British Journal of Psychology*, 95: 387-97.

Grunert, Brad K, Jo M Weis, Mervin R Smucker, and Heidi F Christianson. 2007. 'Imagery rescripting and reprocessing therapy after failed prolonged exposure for post-traumatic stress disorder following industrial injury', *Journal of behavior therapy and experimental psychiatry*, 38: 317-28.

Guédon-Moreau, Laurence, François Ducrocq, Sylvie Molenda, Stéphane Duhem, Julia Salleron, Isabelle Chaudieu, Dina Bert, Christian Libersa, and Guillaume Vaiva. 2012. 'Temporal analysis of heart rate variability as a predictor of post traumatic stress disorder in road traffic accidents survivors', *Journal of psychiatric research*, 46: 790-96.

Guest, Rebecca, Yvonne Tran, Bamini Gopinath, Ian D Cameron, and Ashley Craig. 2018. 'Prevalence and psychometric screening for the detection of major depressive disorder and post-traumatic stress disorder in adults injured in a motor vehicle crash who are engaged in compensation', *BMC psychology*, 6: 1-12.

Gupta, Madhulika A. 2013. 'Review of somatic symptoms in post-traumatic stress disorder', *International Review of Psychiatry*, 25: 86-99.

Haagsma, Juanita A, Suzanne Polinder, Hidde Toet, Martien Panneman, Arie H Havelaar, Gouke J Bonsel, and Ed F van Beeck. 2011. 'Beyond the neglect of psychological consequences: post-traumatic stress disorder increases the non-fatal burden of injury by more than 50%', *Injury Prevention*, 17: 21-26.

Haagsma, Juanita A, Annemieke C Scholten, Teuntje MJC Andriessen, Pieter E Vos, Ed F Van Beeck, and Suzanne Polinder. 2015. 'Impact of depression and post-traumatic stress disorder on functional outcome and health-related quality of life of patients with mild traumatic brain injury', *Journal of neurotrauma*, 32: 853-62.

Hageman, I, HS Andersen, and MB Jørgensen. 2001. 'Post‐traumatic stress disorder: a review of psychobiology and pharmacotherapy', *Acta Psychiatrica Scandinavica*, 104: 411-22.

Haisch, Deanne C, and Lawrence S Meyers. 2004. 'MMPI‐2 assessed post‐traumatic stress disorder related to job stress, coping, and personality in police agencies', *Stress and Health: Journal of the International Society for the Investigation of Stress*, 20: 223-29.

Hall, Katherine S, Katherine D Hoerster, and William S Yancy Jr. 2015. 'Post-traumatic stress disorder, physical activity, and eating behaviors', *Epidemiologic reviews*, 37: 103-15.

Hamanaka, Satoko, Nozomu Asukai, Yoshito Kamijo, Kotaro Hatta, Junji Kishimoto, and Hitoshi Miyaoka. 2006. 'Acute stress disorder and posttraumatic stress disorder symptoms among patients severely injured in motor vehicle accidents in Japan', *General hospital psychiatry*, 28: 234-41.

Hapke, Ulfert, Anja Schumann, Hans-Juerger Rumpf, Ulrich John, and Christian Meyer. 2006. 'Post-traumatic stress disorder: the role of trauma, pre-existing psychiatric disorders, and gender', *European archives of psychiatry and clinical neuroscience*, 256: 299-306.

Haque, Shayla, and Nihar Sultana. 2023. 'Assessment of Post Traumatic Stress Disorder and the Associated Socio Demographic Parameters of Road Traffic Accident Victims', *Journal of Bangladesh College of Physicians and Surgeons*, 41: 22-26.

Haque, Shayla, Nihar Sultana, Nelufar Banu, and Tania Tahsin. 2022. 'Post traumatic stress disorder and the associated factors among the survivors of road traffic accident attending in a specialized hospital in Dhaka', *Update Dental College Journal*, 12: 14-18.

Hartley, Tara A, Khachatur Sarkisian, John M Violanti, Michael E Andrew, and Cecil M Burchfiel. 2013. 'PTSD symptoms among police officers: associations with frequency, recency, and types of traumatic events', *International journal of emergency mental health*, 15: 241.

Hatch, Robert, Duncan Young, Vicki Barber, John Griffiths, David A Harrison, and Peter Watkinson. 2018. 'Anxiety, depression and post traumatic stress disorder after critical illness: a UK-wide prospective cohort study', *Critical care*, 22: 1-13.

Hébert, Martine, Francine Lavoie, and Martin Blais. 2014. 'Post Traumatic Stress Disorder/PTSD in adolescent victims of sexual abuse: resilience and social support as protection factors', *Ciencia & saude coletiva*, 19: 685-94.

Helzer, John E, Lee N Robins, and Larry McEvoy. 1987. 'Post-traumatic stress disorder in the general population', *New England journal of medicine*, 317: 1630-34.

Henson, Charlotte, Didier Truchot, and Amy Canevello. 2021. 'What promotes post traumatic growth? A systematic review', *European Journal of Trauma & Dissociation*, 5: 100195.

Hepp, Urs, Hanspeter Moergeli, Stefan Buchi, Helke Bruchhaus-Steinert, Bernd Kraemer, Tom Sensky, and Ulrich Schnyder. 2008. 'Post-traumatic stress disorder in serious accidental injury: 3-year follow-up study', *The British Journal of Psychiatry*, 192: 376-83.

Herman, Judith Lewis. 1992. 'Complex PTSD: A syndrome in survivors of prolonged and repeated trauma', *Journal of Traumatic stress*, 5: 377-91.

Heron-Delaney, Michelle, Justin Kenardy, Erin Charlton, and Yutaka Matsuoka. 2013. 'A systematic review of predictors of posttraumatic stress disorder (PTSD) for adult road traffic crash survivors', *Injury*, 44: 1413-22.

Herrera-Escobar, Juan Pablo, Elzerie De Jager, Justin Conrad McCarty, Stuart Lipsitz, Adil H Haider, Ali Salim, and Deepika Nehra. 2021. 'Patient-reported outcomes at 6 to 12 months among survivors of firearm injury in the United States', *Annals of surgery*, 274: e1247-e51.

Hickling, Edward J, and Edward B Blanchard. 1992. 'Post-traumatic stress disorder and motor vehicle accidents', *Journal of anxiety disorders*, 6: 285-91.

Högberg, Göran, Marco Pagani, Örjan Sundin, Joaquim Soares, Anna Åberg-Wistedt, Berit Tärnell, and Tore Hällström. 2007. 'On treatment with eye movement desensitization and reprocessing of chronic post-traumatic stress disorder in public transportation workers–a randomized controlled trial', *Nordic journal of psychiatry*, 61: 54-61.

. 2008. 'Treatment of post-traumatic stress disorder with eye movement desensitization and reprocessing: Outcome is stable in 35-month follow-up', *Psychiatry research*, 159: 101-08.

Holbrook, Troy Lisa, Michael R Galarneau, Judy L Dye, Kimberly Quinn, and Amber L Dougherty. 2010. 'Morphine use after combat injury in Iraq and post-traumatic stress disorder', *New England journal of medicine*, 362: 110-17.

Holeva, Vassiliki, and Nicholas Tarrier. 2001. 'Personality and peritraumatic dissociation in the prediction of PTSD in victims of road traffic accidents', *Journal of psychosomatic research*, 51: 687-92.

Holeva, Vassiliki, Nicholas Tarrier, and Adrian Wells. 2001. 'Prevalence and predictors of acute stress disorder and PTSD following road traffic accidents: Thought control strategies and social support', *Behavior therapy*, 32: 65-83.

Holmes, Sal, and NT North. 1998. 'Previously undetected symptoms of post traumatic stress disorder in a population of patients attending a chronic pain clinic', *Journal of Orthopaedic Medicine*, 20: 10-13.

Hong, Chunlan, and Thomas Efferth. 2016. 'Systematic review on post-traumatic stress disorder among survivors of the Wenchuan earthquake', *Trauma, Violence, & Abuse*, 17: 542-61.

Housen, Tambri, Annick Lenglet, Cono Ariti, Showkat Shah, Helal Shah, Shabnum Ara, Kerri Viney, Simon Janes, and Giovanni Pintaldi. 2017. 'Prevalence of anxiety, depression and post-traumatic stress disorder in the Kashmir Valley', *BMJ global health*, 2: e000419.

Howlett, Jonathon R, and Murray B Stein. 2015. 'Post-traumatic stress disorder: relationship to traumatic brain injury and approach to treatment'.

Hu, Hao, Yawen Sun, Shanshan Su, Yao Wang, Yongming Qiu, Xi Yang, Yan Zhou, Zeping Xiao, and Zhen Wang. 2018. 'Cortical surface area reduction in identification of subjects at high risk for post-traumatic stress disorder: A pilot study', *Australian & New Zealand Journal of Psychiatry*, 52: 1084-91.

Hu, Hao, Yan Zhou, Qian Wang, Shanshan Su, Yongming Qiu, Jianwei Ge, Zhen Wang, and Zeping Xiao. 2016. 'Association of abnormal white matter integrity in the acute phase of motor vehicle accidents with post-traumatic stress disorder', *Journal of affective disorders*, 190: 714-22.

Hughes, Katherine C, and Lisa M Shin. 2011. 'Functional neuroimaging studies of post-traumatic stress disorder', *Expert review of neurotherapeutics*, 11: 275-85.

Hull, Alastair M, David A Alexander, and Susan Klein. 2002. 'Survivors of the Piper Alpha oil platform disaster: long-term follow-up study', *The British Journal of Psychiatry*, 181: 433-38.

Husain, Aatif M, Patricia P Miller, and Sandra T Carwile. 2001. 'REM sleep behavior disorder: potential relationship to post-traumatic stress disorder', *Journal of Clinical Neurophysiology*, 18: 148-57.

Husna, Fadhilah Salma, and Heri Kuswoyo. 2022. 'The Portrayal of Post Traumatic Stress Disorder as Seen in The Main Character in the Woman in the Window Novel', *Linguistics and Literature Journal*, 3: 122-30.

Hyman, Irwin A, Wendy Zelikoff, and Jacqueline Clarke. 1988. 'Psychological and physical abuse in the schools: A paradigm for understanding post-traumatic stress disorder in children and youth', *Journal of Traumatic stress*, 1: 243-67.

Igreja, Victor, Wim C Kleijn, Bas JN Schreuder, Janie A Van Dijk, and Margot Verschuur. 2004. 'Testimony method to ameliorate post-traumatic stress symptoms: Community-based intervention study with Mozambican civil war survivors', *The British Journal of Psychiatry*, 184: 251-57.

Iversen, Amy C, Nicola T Fear, Anke Ehlers, J Hacker Hughes, Lisa Hull, Mark Earnshaw, Neil Greenberg, Roberto Rona, Simon Wessely, and Matthew Hotopf. 2008. 'Risk factors for post-traumatic stress disorder among UK Armed Forces personnel', *Psychological medicine*, 38: 511-22.

Iz, Mehmet, Veysi Ceri, Mehmet Emin Layik, and Fatma Betül Ay. 2019. 'Prevalence of post-traumatic stress disorder following unintentional injuries in children', *Eastern Journal of Medicine*, 24: 182-89.

Jaapar, Sharifah Zubaidiah Syed, Zarina Zainan Abidin, and Zahiruddin Othman. 2014. 'Post traumatic stress disorder and its associated risk factors among trauma patients attending the orthopaedic wards and clinics in Kota Bharu, Kelantan', *Int Med J*, 21: 1-3.

Jackson, James C, Robert P Hart, Sharon M Gordon, Ramona O Hopkins, Timothy D Girard, and EWesley Ely. 2007. 'Post-traumatic stress disorder and post-traumatic stress symptoms following critical illness in medical intensive care unit patients: assessing the magnitude of the problem', *Critical care*, 11: 1-11.

Jackson, James C, Pratik P Pandharipande, Timothy D Girard, Nathan E Brummel, Jennifer L Thompson, Christopher G Hughes, Brenda T Pun, Eduard E Vasilevskis, Alessandro Morandi, and Ayumi K Shintani. 2014. 'Depression, post-traumatic stress disorder, and functional disability in survivors of critical illness in the BRAIN-ICU study: a longitudinal cohort study', *The lancet Respiratory medicine*, 2: 369-79.

Jansen, Lina, Michael Hoffmeister, Jenny Chang-Claude, H Brenner, and Volker Arndt. 2011. 'Benefit finding and post-traumatic growth in long-term colorectal cancer survivors: prevalence, determinants, and associations with quality of life', *British journal of cancer*, 105: 1158-65.

Jaspers, Jan PC. 1998. 'Whiplash and post-traumatic stress disorder', *Disability and rehabilitation*, 20: 397-404.

Javidi, Hojjatollah, and M Yadollahie. 2012. 'Post-traumatic stress disorder'.

Jenewein, Josef, Lutz Wittmann, Hanspeter Moergeli, J Creutzig, and U Schnyder. 2009. 'Mutual influence of posttraumatic stress disorder symptoms and chronic pain among injured accident survivors: a longitudinal study', *Journal of Traumatic Stress: Official Publication of The International Society for Traumatic Stress Studies*, 22: 540-48.

Jin, C, R Qi, Y Yin, X Hu, L Duan, Q Xu, Z Zhang, Y Zhong, B Feng, and H Xiang. 2014. 'Abnormalities in whole-brain functional connectivity observed in treatment-naive post-traumatic stress disorder patients following an earthquake', *Psychological medicine*, 44: 1927-36.

Jin, Yuchang, Jiuping Xu, and Dongyue Liu. 2014. 'The relationship between post traumatic stress disorder and post traumatic growth: gender differences in PTG and PTSD subgroups', *Social psychiatry and psychiatric epidemiology*, 49: 1903-10.

Jin, Yuchang, Jiuping Xu, Hai Liu, and Dongyue Liu. 2014. 'Posttraumatic stress disorder and posttraumatic growth among adult survivors of Wenchuan earthquake after 1 year: Prevalence and correlates', *Archives of Psychiatric Nursing*, 28: 67-73.

Jobson, Laura, and Richard O'Kearney. 2008. 'Cultural differences in personal identity in post‐traumatic stress disorder', *British Journal of Clinical Psychology*, 47: 95-109.

Johannesson, Kerstin Bergh, Tom Lundin, Thomas Fröjd, Christina M Hultman, and Per-Olof Michel. 2011. 'Tsunami-exposed tourist survivors: signs of recovery in a 3-year perspective', *The Journal of Nervous and Mental Disease*, 199: 162-69.

Johansen, Venke A, Astrid K Wahl, Dag Erik Eilertsen, and Lars Weisaeth. 2007. 'Prevalence and predictors of post-traumatic stress disorder (PTSD) in physically injured victims of non-domestic violence: A longitudinal study', *Social psychiatry and psychiatric epidemiology*, 42: 583-93.

Johansen, Venke A, Astrid K Wahl, Dag Erik Eilertsen, Lars Weisaeth, and Berit R Hanestad. 2007. 'The predictive value of post-traumatic stress disorder symptoms for quality of life: a longitudinal study of physically injured victims of non-domestic violence', *Health and quality of life outcomes*, 5: 1-11.

John, Prashantham Baddam, Sushila Russell, and Paul Swamidhas Sudhakar Russell. 2007. 'The prevalence of posttraumatic stress disorder among children and adolescents affected by tsunami disaster in Tamil Nadu', *Disaster management & response*, 5: 3-7.

Johnsen, Bjørn Helge, Jarlc Eid, Jon Christian Laberg, and Julian’F Thayer. 2002. 'The effect of sensitization and coping style on post-traumatic stress symptoms and quality of life: two longitudinal studies', *Scandinavian journal of psychology*, 43.

Johnson, Howard, and Andrew Thompson. 2008. 'The development and maintenance of post-traumatic stress disorder (PTSD) in civilian adult survivors of war trauma and torture: A review', *Clinical psychology review*, 28: 36-47.

Jones, Charlie, Allison G Harvey, and Chris R Brewin. 2005. 'Traumatic brain injury, dissociation, and posttraumatic stress disorder in road traffic accident survivors', *Journal of Traumatic Stress: Official Publication of The International Society for Traumatic Stress Studies*, 18: 181-91.

. 2007. 'The organisation and content of trauma memories in survivors of road traffic accidents', *Behaviour research and therapy*, 45: 151-62.

Jones, Loring, Margaret Hughes, and Ulrike Unterstaller. 2001. 'Post-traumatic stress disorder (PTSD) in victims of domestic violence: A review of the research', *Trauma, Violence, & Abuse*, 2: 99-119.

Jonsson, Anders, and Kerstin Segesten. 2004. 'Guilt, shame and need for a container: a study of post-traumatic stress among ambulance personnel', *Accident and emergency nursing*, 12: 215-23.

Jonsson, Anders, Kerstin Segesten, and B Mattsson. 2003. 'Post-traumatic stress among Swedish ambulance personnel', *Emergency medicine journal*, 20: 79-84.

Joseph, Stephen, Tim Dalgleish, Ruth Williams, William Yule, Sian Thrasher, and Peter Hodgkinson. 1997. 'Attitudes towards emotional expression and post‐traumatic stress in survivors of the Herald of Free Enterprise disaster', *British Journal of Clinical Psychology*, 36: 133-38.

Joseph, Stephen, David Murphy, and Stephen Regel. 2012. 'An affective–cognitive processing model of post‐traumatic growth', *Clinical psychology & psychotherapy*, 19: 316-25.

Joseph, Stephen, Ruth Williams, and William Yule. 1995. 'Psychosocial perspectives on post-traumatic stress', *Clinical psychology review*, 15: 515-44.

. 1997. 'Understanding Post-traumatic Stress: A Psychosocial'.

Joseph, Stephen, William Yule, and Ruth Williams. 1993. 'Post-traumatic stress: Attributional aspects', *Journal of Traumatic stress*, 6: 501-13.

Joseph, Steve, Ruth Williams, and William Yule. 1992. 'Crisis support, attributional style, coping style, and post-traumatic symptoms', *Personality and Individual Differences*, 13: 1249-51.

Jowett, Sally, Thanos Karatzias, and Idit Albert. 2020. 'Multiple and interpersonal trauma are risk factors for both post‐traumatic stress disorder and borderline personality disorder: A systematic review on the traumatic backgrounds and clinical characteristics of comorbid post‐traumatic stress disorder/borderline personality disorder groups versus single‐disorder groups', *Psychology and Psychotherapy: Theory, Research and Practice*, 93: 621-38.

Jubran, Amal, Gerald Lawm, Lisa A Duffner, Eileen G Collins, Dorothy M Lanuza, Leslie A Hoffman, and Martin J Tobin. 2010. 'Post-traumatic stress disorder after weaning from prolonged mechanical ventilation', *Intensive care medicine*, 36: 2030-37.

Kabunga, Amir, Ponsiano Okalo, Viola Nalwoga, and Brenda Apili. 2022. 'Landslide disasters in eastern Uganda: post-traumatic stress disorder and its correlates among survivors in Bududa district', *BMC psychology*, 10: 287.

Kaminer, Debra, Soraya Seedat, and Dan J Stein. 2005. 'Post-traumatic stress disorder in children', *World psychiatry*, 4: 121.

Kamkuimo, Sorelle Audrey, Benoît Girard, and Bob-Antoine J Menelas. 2021. 'A narrative review of virtual reality applications for the treatment of post-traumatic stress disorder', *Applied Sciences*, 11: 6683.

Kanani, K, S Hadi, and NP Tayebi. 2015. 'The effectiveness of acceptance and commitment therapy on treating the adolescents with post-traumatic stress disorder after road accidents in the province of Isfahan', *Iranian Journal of Rehabilitation Research*, 1: 22-32.

Kang, Sung-Gil, Ju-Wan Kim, Hee-Ju Kang, Hyunseok Jang, Jung-Chul Kim, Ju-Yeon Lee, Sung-Wan Kim, Il-Seon Shin, and Jae-Min Kim. 2024. 'Differential predictors of early-and delayed-onset post-traumatic stress disorder following physical injury: a two-year longitudinal study', *Frontiers in psychiatry*, 15: 1367661.

Kaplan, Gary B, Jennifer J Vasterling, and Priyanka C Vedak. 2010. 'Brain-derived neurotrophic factor in traumatic brain injury, post-traumatic stress disorder, and their comorbid conditions: role in pathogenesis and treatment', *Behavioural pharmacology*, 21: 427-37.

Kar, Nilamadhab. 2009. 'Psychological impact of disasters on children: review of assessment and interventions', *World journal of pediatrics*, 5: 5-11.

. 2011. 'Cognitive behavioral therapy for the treatment of post-traumatic stress disorder: a review', *Neuropsychiatric disease and treatment*: 167-81.

Kar, Nilamadhab, and Binaya Kumar Bastia. 2006. 'Post-traumatic stress disorder, depression and generalised anxiety disorder in adolescents after a natural disaster: a study of comorbidity', *Clinical Practice and Epidemiology in Mental Health*, 2: 1-7.

Kar, Nilamadhab, Prasanta K Mohapatra, Kailash C Nayak, Pratiti Pattanaik, Sarada P Swain, and Harish C Kar. 2007. 'Post-traumatic stress disorder in children and adolescents one year after a super-cyclone in Orissa, India: exploring cross-cultural validity and vulnerability factors', *BMC psychiatry*, 7: 1-9.

Karanci, Nuray A, and Acarturk. 2005. 'Post-traumatic growth among Marmara earthquake survivors involved in disaster preparedness as volunteers', *Traumatology*, 11: 307-23.

Karunakara, Unni Krishnan, Frank Neuner, Margarete Schauer, Kavita Singh, Kenneth Hill, Thomas Elbert, and Gilbert Burnham. 2004. 'Traumatic events and symptoms of post-traumatic stress disorder amongst Sudanese nationals, refugees and Ugandans in the West Nile', *African health sciences*, 4: 83-93.

Kaseda, Erin T, and Andrew J Levine. 2020. 'Post-traumatic stress disorder: A differential diagnostic consideration for COVID-19 survivors', *The Clinical Neuropsychologist*, 34: 1498-514.

Kashdan, Todd B, Nexhmedin Morina, and Stefan Priebe. 2009. 'Post-traumatic stress disorder, social anxiety disorder, and depression in survivors of the Kosovo War: Experiential avoidance as a contributor to distress and quality of life', *Journal of anxiety disorders*, 23: 185-96.

Kassa, Mulat Awoke, Sefineh Fenta, Tamrat Anbesaw, Natnael Amare Tesfa, Alemu Birara Zemariam, Genanew Mulugeta Kassaw, Biruk Beletew Abate, and Elsabet Gezmu Semagn. 2024. 'Post-traumatic stress disorder and associated factors among high school students who experienced war in Woldia town', *Frontiers in psychiatry*, 15: 1359370.

Kazantzis, Nikolaos, James Kennedy-Moffat, Ross A Flett, Alexandra M Petrik, Nigel R Long, and Bronwyn Castell. 2012. 'Predictors of chronic trauma-related symptoms in a community sample of New Zealand motor vehicle accident survivors', *Culture, medicine, and psychiatry*, 36: 442-64.

Kaźmierczak, Izabela, Jan Strelau, and Bogdan Zawadzki. 2016. 'Post-traumatic cognitions as a mediator of the relationship between sense of coherence and post-traumatic stress disorder after motor vehicle accidents', *Transportation research part F: traffic psychology and behaviour*, 41: 38-44.

Kazour, Francois, Nada R Zahreddine, Michel G Maragel, Mustafa A Almustafa, Michel Soufia, Ramzi Haddad, and Sami Richa. 2017. 'Post-traumatic stress disorder in a sample of Syrian refugees in Lebanon', *Comprehensive psychiatry*, 72: 41-47.

Keane, Terence M, John A Fairbank, Juesta M Caddell, Rose T Zimering, and Mary E Bender. 2013. 'A behavioral approach to assessing and treating post-traumatic stress disorder in Vietnam veterans.' in, *Trauma and its wake* (Routledge).

Kemp, Anita, Edna I Rawlings, and Bonnie L Green. 1991. 'Post‐traumatic stress disorder (PTSD) in battered women: A shelter sample', *Journal of Traumatic stress*, 4: 137-48.

Kemp, Michael, Peter Drummond, and Brett McDermott. 2010. 'A wait-list controlled pilot study of eye movement desensitization and reprocessing (EMDR) for children with post-traumatic stress disorder (PTSD) symptoms from motor vehicle accidents', *Clinical child psychology and psychiatry*, 15: 5-25.

Kenardy, Justin, and Rachael Dunne. 2011. 'Traumatic injury and traumatic stress', *Spine*, 36: S233-S37.

Kennedy, Paul, and Jane Duff. 2001. 'Post traumatic stress disorder and spinal cord injuries', *Spinal Cord*, 39: 1-10.

Keppel‐Benson, Jane M, Thomas H Ollendick, and Mark J Benson. 2002. 'Post‐traumatic stress in children following motor vehicle accidents', *Journal of child psychology and psychiatry*, 43: 203-12.

Khamis, Vivian. 2005. 'Post-traumatic stress disorder among school age Palestinian children', *Child abuse & neglect*, 29: 81-95.

. 2008. 'Post-traumatic stress and psychiatric disorders in Palestinian adolescents following intifada-related injuries', *Social Science & Medicine*, 67: 1199-207.

Khodadadi-Hassankiadeh, Naema, Nahid Dehghan Nayeri, Hooman Shahsavari, Shahrokh Yousefzadeh-Chabok, and Hamid Haghani. 2017. 'Predictors of post-traumatic stress disorder among victims of serious motor vehicle accidents', *International journal of community based nursing and midwifery*, 5: 355.

Kienzler, Hanna. 2008. 'Debating war-trauma and post-traumatic stress disorder (PTSD) in an interdisciplinary arena', *Social Science & Medicine*, 67: 218-27.

Kılıç, C, KM Magruder, and MM Koryürek. 2016. 'Does trauma type relate to posttraumatic growth after war? A pilot study of young Iraqi war survivors living in Turkey', *Transcultural psychiatry*, 53: 110-23.

Kılıç, Cengiz, İsmail Aydın, Nilgün Taşkıntuna, Gamze Özçürümez, Gökben Kurt, Emine Eren, Tevfik Lale, S Özel, and Leyla Zileli. 2006. 'Predictors of psychological distress in survivors of the 1999 earthquakes in Turkey: effects of relocation after the disaster', *Acta Psychiatrica Scandinavica*, 114: 194-202.

Kilpatrick, Dean G, Benjamin E Saunders, Angelynne Amick-McMullan, Connie L Best, Lois J Veronen, and Heidi S Resnick. 1989. 'Victim and crime factors associated with the development of crime-related post-traumatic stress disorder', *Behavior therapy*, 20: 199-214.

Kilpatrick, Kym L, and Leanne M Williams. 1997. 'Post-traumatic stress disorder in child witnesses to domestic violence', *American journal of orthopsychiatry*, 67: 639.

. 1998. 'Potential mediators of post-traumatic stress disorder in child witnesses to domestic violence', *Child abuse & neglect*, 22: 319-30.

Kim, Jae-Min, Hee-Ju Kang, Ju-Wan Kim, Ju-Yeon Lee, Hyunseok Jang, Jung-Chul Kim, Sung-Wan Kim, and Il-Seon Shin. 2025. 'Longitudinal associations between early post-injury serum BDNF levels and the development of post-traumatic stress disorder over two years', *Journal of affective disorders*, 369: 483-88.

Kim, Jae-Min, Ju-Wan Kim, Hee-Ju Kang, Ye-Jin Kim, Hyunseok Jang, Jung-Chul Kim, Sung-Wan Kim, Il-Seon Shin, and Robert Stewart. 2024. 'Predictors of 2‐Year Trajectory of Post‐Traumatic Stress Disorder Following Physical Injury', *Depression and Anxiety*, 2024: 5570405.

Kim, Yoonjung. 2022. "Factors Associated With Post-Traumatic Growth in Korean Survivors of Childhood Cancer." In *Oncology Nursing Forum*.

Kinchin, David. 2007. *A guide to psychological debriefing: Managing emotional decompression and post-traumatic stress disorder* (Jessica Kingsley Publishers).

King, Jean A, Deborah Mandansky, Susie King, Kennethe E Fletcher, and Judith Brewer. 2001. 'Early sexual abuse and low cortisol', *Psychiatry and clinical neurosciences*, 55: 71-74.

King, Lynda A, Daniel W King, John A Fairbank, Terence M Keane, and Gary A Adams. 1998. 'Resilience–recovery factors in post-traumatic stress disorder among female and male Vietnam veterans: Hardiness, postwar social support, and additional stressful life events', *Journal of personality and social psychology*, 74: 420.

Kira, Ibrahim A. 2010. 'Etiology and treatment of post-cumulative traumatic stress disorders in different cultures', *Traumatology*, 16: 128.

Kiser, Laurel J, Jerry Heston, Pamela A Millsap, and David B Pruitt. 1991. 'Physical and sexual abuse in childhood: Relationship with post-traumatic stress disorder', *Journal of the American Academy of Child & Adolescent Psychiatry*, 30: 776-83.

Kleim, B, T Ehring, and A Ehlers. 2012. 'Perceptual processing advantages for trauma-related visual cues in post-traumatic stress disorder', *Psychological medicine*, 42: 173-81.

Kleim, Birgit, Anke Ehlers, and Edward Glucksman. 2007. 'Early predictors of chronic post-traumatic stress disorder in assault survivors', *Psychological medicine*, 37: 1457-67.

Klein, Ehud, Danny Koren, Isaac Arnon, and Peretz Lavie. 2003. 'Sleep complaints are not corroborated by objective sleep measures in post‐traumatic stress disorder: a 1‐year prospective study in survivors of motor vehicle crashes', *Journal of sleep research*, 12: 35-41.

Koch, William J. 2002. 'Post-traumatic stress disorder and pain following motor vehicle collisions', *British Columbia Medical Journal*, 44: 298-302.

Koenen, Karestan C, Jennifer A Sumner, Paola Gilsanz, M Maria Glymour, Andrew Ratanatharathorn, Eric B Rimm, Andrea L Roberts, Ashley Winning, and Laura D Kubzansky. 2017. 'Post-traumatic stress disorder and cardiometabolic disease: improving causal inference to inform practice', *Psychological medicine*, 47: 209-25.

Koenigs, Michael, Edward D Huey, Vanessa Raymont, Bobby Cheon, Jeffrey Solomon, Eric M Wassermann, and Jordan Grafman. 2008. 'Focal brain damage protects against post-traumatic stress disorder in combat veterans', *Nature neuroscience*, 11: 232-37.

Kokai, Masahiro, Senta Fujii, Naotaka Shinfuku, and Glen Edwards. 2004. 'Natural disaster and mental health in Asia', *Psychiatry and clinical neurosciences*, 58: 110-16.

Kolkow, Tonya T, James L Spira, Jennifer S Morse, and Thomas A Grieger. 2007. 'Post-traumatic stress disorder and depression in health care providers returning from deployment to Iraq and Afghanistan', *Military medicine*, 172: 451-55.

Koopman, Cheryl, Catherine Classen, Etzel Cardeña, and David Spiegel. 1995. 'When disaster strikes, acute stress disorder may follow', *Journal of Traumatic stress*, 8: 29-46.

Koren, Danny, Isaac Arnon, and Ehud Klein. 1999. 'Acute stress response and posttraumatic stress disorder in traffic accident victims: a one-year prospective, follow-up study', *American Journal of Psychiatry*, 156: 367-73.

Kovacevic, Jelena, Maja Miskulin, Dunja Degmecic, Aleksandar Vcev, Dinko Leovic, Vladimir Sisljagic, Ivana Simic, Hrvoje Palenkic, Ivan Vcev, and Ivan Miskulin. 2020. 'Predictors of mental health outcomes in road traffic accident survivors', *Journal of clinical medicine*, 9: 309.

Krakow, Barry, Carmen Lowry, Anne Germain, Lane Gaddy, Michael Hollifield, Mary Koss, Dan Tandberg, Lisa Johnston, and Dominic Melendrez. 2000. 'A retrospective study on improvements in nightmares and post-traumatic stress disorder following treatment for co-morbid sleep-disordered breathing', *Journal of psychosomatic research*, 49: 291-98.

Kramer, Teresa L, Jacob D Lindy, Bonnie L Green, Mary C Grace, and Anthony C Leonard. 1994. 'The comorbidity of post‐traumatic stress disorder and suicidality in Vietnam veterans', *Suicide and Life‐Threatening Behavior*, 24: 58-67.

Krauss, Joachim K, Rolf Trankle, and Karl-Heinz Kopp. 1996. 'Post-traumatic movement disorders in survivors of severe head injury', *Neurology*, 47: 1488-92.

Kravets, Victoria, Michael McDonald, Joel DeRosa, Roberto Hernandez-Irizarry, Ruth Parker, Dorian A Lamis, Abigail Powers, and Mara L Schenker. 2023. 'Early identification of post-traumatic stress disorder in trauma patients: development of a multivariable risk prediction model', *The American Surgeon™*, 89: 4542-51.

Krysinska, Karolina, and David Lester. 2010. 'Post-traumatic stress disorder and suicide risk: a systematic review', *Archives of suicide research*, 14: 1-23.

Kukihara, Hiroko, Niwako Yamawaki, Kumi Uchiyama, Shoichi Arai, and Etsuo Horikawa. 2014. 'Trauma, depression, and resilience of earthquake/tsunami/nuclear disaster survivors of H irono, F ukushima, J apan', *Psychiatry and clinical neurosciences*, 68: 524-33.

Kumar, Rajeet, and Anita Thakur. 'Assessment of incidence of post-traumatic stress disorder among patients with history of road traffic accident', *Anxiety*, 6: 28.57.

Kun, P, X Chen, S Han, X Gong, Mei Chen, W Zhang, and L Yao. 2009. 'Prevalence of post-traumatic stress disorder in Sichuan Province, China after the 2008 Wenchuan earthquake', *Public health*, 123: 703-07.

Kupchik, Marina, Rael D Strous, Rina Erez, Noach Gonen, Abraham Weizman, and Baruch Spivak. 2007. 'Demographic and clinical characteristics of motor vehicle accident victims in the community general health outpatient clinic: a comparison of PTSD and non‐PTSD subjects', *Depression and Anxiety*, 24: 244-50.

Kwak, Minyoung, Brad J Zebrack, Kathleen A Meeske, Leanne Embry, Christine Aguilar, Rebecca Block, Brandon Hayes‐Lattin, Yun Li, Melissa Butler, and Steven Cole. 2013. 'Prevalence and predictors of post‐traumatic stress symptoms in adolescent and young adult cancer survivors: a 1‐year follow‐up study', *Psycho‐Oncology*, 22: 1798-806.

Laffaye, Charlene, Craig S Rosen, Paula P Schnurr, and Matthew J Friedman. 2007. 'Does compensation status influence treatment participation and course of recovery from post-traumatic stress disorder?', *Military medicine*, 172: 1039-45.

Landolt, Markus A, U Boehler, C Schwager, U Schallberger, and R Nuessli. 1998. 'Post‐traumatic stress disorder in paediatric patients and their parents: an exploratory study', *Journal of paediatrics and child health*, 34: 539-43.

Landolt, Markus A, Margarete E Vollrath, Hanspeter E Gnehm, and Felix H Sennhauser. 2009. 'Post-traumatic stress impacts on quality of life in children after road traffic accidents: Prospective study', *Australian & New Zealand Journal of Psychiatry*, 43: 746-53.

Langford, Dale J, Brian R Theodore, Danica Balsiger, Christine Tran, Ardith Z Doorenbos, David J Tauben, and Mark D Sullivan. 2018. 'Number and type of post-traumatic stress disorder symptom domains are associated with patient-reported outcomes in patients with chronic pain', *The Journal of Pain*, 19: 506-14.

Lawrence, John W, and James A Fauerbach. 2003. 'Personality, coping, chronic stress, social support and PTSD symptoms among adult burn survivors: a path analysis', *The Journal of Burn Care & Rehabilitation*, 24: 63-72.

Lee, Alyssa, Mohan Isaac, and Aleksandar Janca. 2002. 'Post-traumatic stress disorder and terrorism', *Current Opinion in Psychiatry*, 15: 633-37.

Lee, Deborah, and Kerry Young. 2001. 'Post-traumatic stress disorder: Diagnostic issues and epidemiology in adult survivors of traumatic events', *International Review of Psychiatry*, 13: 150-58.

Lee, Eunjung, Jessie Faber, and Kathryn Bowles. 2022. 'A review of trauma specific treatments (tsts) for post-traumatic stress disorder (PTSD)', *Clinical Social Work Journal*, 50: 147-59.

Lee, Kang Soo, So Young Joo, Cheong Hoon Seo, Joo-Eon Park, and Boung Chul Lee. 2019. 'Work-related burn injuries and claims for post-traumatic stress disorder in Korea', *Burns*, 45: 461-65.

Lee, So Hee, Hyoung-Shik Shin, Hye Yoon Park, Jeong Lan Kim, Jung Jae Lee, Haewoo Lee, Sung-Doo Won, and Woori Han. 2019. 'Depression as a mediator of chronic fatigue and post-traumatic stress symptoms in Middle East respiratory syndrome survivors', *Psychiatry investigation*, 16: 59.

Lee, Ya-Ling, Bih-Shya Gau, Wen-Ming Hsu, and Hsiu-Hao Chang. 2009. "A model linking uncertainty, post-traumatic stress, and health behaviors in childhood cancer survivors." In *Oncology Nursing Forum*.

Lever, Hazel, Deborah Ottenheimer, Jimmitti Teysir, Elizabeth Singer, and Holly G Atkinson. 2019. 'Depression, anxiety, post-traumatic stress disorder and a history of pervasive gender-based violence among women asylum seekers who have undergone female genital mutilation/cutting: a retrospective case review', *Journal of immigrant and minority health*, 21: 483-89.

Lewis, Catrin, Neil P Roberts, Martin Andrew, Elise Starling, and Jonathan I Bisson. 2020. 'Psychological therapies for post-traumatic stress disorder in adults: Systematic review and meta-analysis', *European Journal of Psychotraumatology*, 11: 1729633.

Lewis, Catrin, Neil P Roberts, Samuel Gibson, and Jonathan I Bisson. 2020. 'Dropout from psychological therapies for post-traumatic stress disorder (PTSD) in adults: Systematic review and meta-analysis', *European Journal of Psychotraumatology*, 11: 1709709.

Lewis, Stephanie J, Louise Arseneault, Avshalom Caspi, Helen L Fisher, Timothy Matthews, Terrie E Moffitt, Candice L Odgers, Daniel Stahl, Jia Ying Teng, and Andrea Danese. 2019. 'The epidemiology of trauma and post-traumatic stress disorder in a representative cohort of young people in England and Wales', *The Lancet Psychiatry*, 6: 247-56.

Leymann, Heinz, and Annelie Gustafsson. 1996. 'Mobbing at work and the development of post-traumatic stress disorders', *European journal of work and organizational psychology*, 5: 251-75.

Li, Qi, Wei Liu, Jie-Yu Wang, Xiao-Guang Wang, Bo Hao, Yu-Bo Hu, Xi Deng, Lu Liu, Hu Zhao, and Yan-Wei Shi. 2023. 'Prevalence and risk factors of post-traumatic stress disorder symptoms among Chinese health care workers following the COVID-19 pandemic', *Heliyon*, 9.

Liao, Jingping, Xiaofang Ma, Bin Gao, Mingfeng Zhang, Yuanfang Zhang, Maoqun Liu, and Xiaolin Li. 2019. 'Psychological status of nursing survivors in China and its associated factors: 6 years after the 2008 Sichuan earthquake', *Neuropsychiatric disease and treatment*: 2301-11.

Liberzon, Israel, and James L Abelson. 2016. 'Context processing and the neurobiology of post-traumatic stress disorder', *Neuron*, 92: 14-30.

Liedl, A, M O'donnell, M Creamer, D Silove, A McFarlane, C Knaevelsrud, and RA Bryant. 2010. 'Support for the mutual maintenance of pain and post-traumatic stress disorder symptoms', *Psychological medicine*, 40: 1215-23.

Lin, Wanli, Lina Gong, Miaojuan Xia, and Wenjie Dai. 2018. 'Prevalence of posttraumatic stress disorder among road traffic accident survivors: A PRISMA-compliant meta-analysis', *Medicine*, 97: e9693.

Lindauer, Ramon JL, Erik-Jan Vlieger, Margje Jalink, Miranda Olff, Ingrid VE Carlier, Charles BLM Majoie, Gerard J Den Heeten, and Berthold PR Gersons. 2005. 'Effects of psychotherapy on hippocampal volume in out-patients with post-traumatic stress disorder: a MRI investigation', *Psychological medicine*, 35: 1421-31.

Liriano, Felix, Candace Hatten, and Thomas L Schwartz. 2019. 'Ketamine as treatment for post-traumatic stress disorder: a review', *Drugs in context*, 8.

Lisieski, Michael J, Andrew L Eagle, Alana C Conti, Israel Liberzon, and Shane A Perrine. 2018. 'Single-prolonged stress: a review of two decades of progress in a rodent model of post-traumatic stress disorder', *Frontiers in psychiatry*, 9: 196.

Livanou, Maria, Yiannis Kasvikis, Metin Başoğlu, Pashalia Mytskidou, Vivi Sotiropoulou, Efrosyni Spanea, Titika Mitsopoulou, and Nikoletta Voutsa. 2005. 'Earthquake-related psychological distress and associated factors 4 years after the Parnitha earthquake in Greece', *European Psychiatry*, 20: 137-44.

Logue, Mark W, Clinton Baldwin, Guia Guffanti, Efi Melista, Erika J Wolf, Annemarie F Reardon, Monica Uddin, Derek Wildman, Sandro Galea, and Karestan C Koenen. 2013. 'A genome-wide association study of post-traumatic stress disorder identifies the retinoid-related orphan receptor alpha (RORA) gene as a significant risk locus', *Molecular psychiatry*, 18: 937-42.

Lohr, James B, Barton W Palmer, Carolyn A Eidt, Smitha Aailaboyina, Brent T Mausbach, Owen M Wolkowitz, Steven R Thorp, and Dilip V Jeste. 2015. 'Is post-traumatic stress disorder associated with premature senescence? A review of the literature', *The American Journal of Geriatric Psychiatry*, 23: 709-25.

Long, Zhiliang, XuJun Duan, Bing Xie, Handan Du, Rong Li, Qiang Xu, Luqing Wei, Shao-xiang Zhang, Yi Wu, and Qing Gao. 2013. 'Altered brain structural connectivity in post-traumatic stress disorder: a diffusion tensor imaging tractography study', *Journal of affective disorders*, 150: 798-806.

LoSavio, Stefanie T, Kirsten H Dillon, and Patricia A Resick. 2017. 'Cognitive factors in the development, maintenance, and treatment of post-traumatic stress disorder', *Current opinion in psychology*, 14: 18-22.

Lowenstein, LF. 2001. 'Post-traumatic stress reactions in victims of motor accidents', *The Police Journal*, 74: 9-25.

Ma, Sisi, Isaac R Galatzer-Levy, Xuya Wang, David Fenyö, and Arieh Y Shalev. 2017. "A first step towards a clinical decision support system for post-traumatic stress disorders." In *AMIA Annual Symposium Proceedings*, 837.

Ma, Xiaohong, Xiang Liu, Xun Hu, Changjian Qiu, Yingcheng Wang, Yi Huang, Qiang Wang, Wei Zhang, and Tao Li. 2011. 'Risk indicators for post-traumatic stress disorder in adolescents exposed to the 5.12 Wenchuan earthquake in China', *Psychiatry research*, 189: 385-91.

Machisa, Mercilene T, Nicola Christofides, and Rachel Jewkes. 2018. 'Social support factors associated with psychological resilience among women survivors of intimate partner violence in Gauteng, South Africa', *Global health action*, 11: 1491114.

Macleod, Alistair D. 1994. 'The reactivation of post-traumatic stress disorder in later life', *Australian and New Zealand Journal of Psychiatry*, 28: 625-34.

Maercker, Andreas, and Mareike Augsburger. 2022. 'Post-traumatic Stress Disorder.' in, *Trauma Sequelae* (Springer).

Maercker, Andreas, and Julia Müller. 2004. 'Social acknowledgment as a victim or survivor: A scale to measure a recovery factor of PTSD', *Journal of Traumatic stress*, 17: 345-51.

Maes, M, A Lin, S Bonacccorso, F Van Hunsel, A Van Gastel, L Delmeire, M Biondi, E Bosmans, G Kenis, and S Scharpe. 1998. 'Increased 24‐hour urinary cortisol excretion in patients with post‐traumatic stress disorder and patients with major depression, but not h patients with fibromyalgia', *Acta Psychiatrica Scandinavica*, 98: 328-35.

Maes, Michael, Laure Delmeire, Jacques Mylle, and Carlo Altamura. 2001. 'Risk and preventive factors of post-traumatic stress disorder (PTSD): alcohol consumption and intoxication prior to a traumatic event diminishes the relative risk to develop PTSD in response to that trauma', *Journal of affective disorders*, 63: 113-21.

Maes, Michael, Laure Delmeire, Chris Schotte, Aleksandar Janca, Thomas Creten, Jacques Mylle, Anja Struyf, Greet Pison, and Peter J Rousseeuw. 1998. 'Epidemiologic and phenomenological aspects of post-traumatic stress disorder: DSM-III-R diagnosis and diagnostic criteria not validated', *Psychiatry research*, 81: 179-93.

Maes, Michael, Jacques Mylle, Laure Delmeire, and Carlo Altamura. 2000. 'Psychiatric morbidity and comorbidity following accidental man-made traumatic events: incidence and risk factors', *European archives of psychiatry and clinical neuroscience*, 250: 156-62.

Maes, Michael, Jacques Mylle, Laure Delmeire, and Alexander Janca. 2001. 'Pre-and post-disaster negative life events in relation to the incidence and severity of post-traumatic stress disorder', *Psychiatry research*, 105: 1-12.

Maher, Michael J, Simon A Rego, and Gregory M Asnis. 2006. 'Sleep disturbances in patients with post-traumatic stress disorder: epidemiology, impact and approaches to management', *CNS drugs*, 20.

Mak, Ivan Wing Chit, Chung Ming Chu, Pey Chyou Pan, Michael Gar Chung Yiu, and Veronica Lee Chan. 2009. 'Long-term psychiatric morbidities among SARS survivors', *General hospital psychiatry*, 31: 318-26.

Mak, Ivan Wing Chit, Chung Ming Chu, Pey Chyou Pan, Michael Gar Chung Yiu, Suzanne C Ho, and Veronica Lee Chan. 2010. 'Risk factors for chronic post-traumatic stress disorder (PTSD) in SARS survivors', *General hospital psychiatry*, 32: 590-98.

Manne, Sharon, Katherine Duhamel, and William H Redd. 2000. 'Association of psychological vulnerability factors to post‐traumatic stress symptomatology in mothers of pediatric cancer survivors', *Psycho‐Oncology: Journal of the Psychological, Social and Behavioral Dimensions of Cancer*, 9: 372-84.

Marais, Andrea, and AD Stuart. 2005. 'The role of temperament in the development of post-traumatic stress disorder amongst journalists', *South African Journal of Psychology*, 35: 89-105.

Marina, Yuabova, and Parrinello Michael. 2013. 'Posttraumatic stress disorder among motor vehicle accident victims', *Russian Open Medical Journal*, 2: 0206.

Markowitz, John C, Barbara Milrod, Kathryn Bleiberg, and Randall D Marshall. 2009. 'Interpersonal factors in understanding and treating posttraumatic stress disorder', *Journal of Psychiatric Practice®*, 15: 133-40.

Mason, Suzanne, and Alison Rowlands. 1997. 'Post-traumatic stress disorder', *Journal of accident & emergency medicine*, 14: 387.

Masuwa, Kaluso C. 2017. 'Prior camp experiences and stress related to post traumatic stress disorder (PTSD) among refugees at Maheba refugee settlement in Zambia', The University of Zambia.

Matsumura, Kenta, Hiroko Noguchi, Daisuke Nishi, Kei Hamazaki, Tomohito Hamazaki, and Yutaka J Matsuoka. 2017. 'Effects of omega-3 polyunsaturated fatty acids on psychophysiological symptoms of post-traumatic stress disorder in accident survivors: A randomized, double-blind, placebo-controlled trial', *Journal of affective disorders*, 224: 27-31.

Matthews, Lynda R. 2005. 'Work potential of road accident survivors with post-traumatic stress disorder', *Behaviour research and therapy*, 43: 475-83.

Matthiesen, Stig Berge, and Ståle Einarsen. 2004. 'Psychiatric distress and symptoms of PTSD among victims of bullying at work', *British journal of guidance & counselling*, 32: 335-56.

Matu, Kenneth. 2022. 'Relationship Between Injury Severity Score & Post-traumatic Stress Disorder Among Orthopaedic Trauma Surgical Patients at the Kenyatta National Hospital', University of Nairobi.

Mayou, RA, A Ehlers, and M Hobbs. 2000. 'Psychological debriefing for road traffic accident victims: Three-year follow-up of a randomised controlled trial', *The British Journal of Psychiatry*, 176: 589-93.

Mayou, Richard. 2021. 'The psychiatry of road traffic accidents', *The Aftermath of Road Accidents*: 33-48.

Mayou, Richard A, John Black, and Bridget Bryant. 2000. 'Unconsciousness, amnesia and psychiatric symptoms following road traffic accident injury', *The British Journal of Psychiatry*, 177: 540-45.

Mayou, Richard, and Bridget Bryant. 2001. 'Outcome in consecutive emergency department attenders following a road traffic accident', *The British Journal of Psychiatry*, 179: 528-34.

Mayou, Richard, Bridget Bryant, and Robert Duthie. 1993. 'Psychiatric consequences of road traffic accidents', *British Medical Journal*, 307: 647-51.

McDermott, Brett M, and Anita Cvitanovich. 2000. 'Posttraumatic stress disorder and emotional problems in children following motor vehicle accidents: an extended case series', *Australian & New Zealand Journal of Psychiatry*, 34: 446-52.

McElheran, Megan, Allison Briscoe-Smith, Anna Khaylis, Darrah Westrup, Chris Hayward, and Cheryl Gore-Felton. 2012. 'A conceptual model of post-traumatic growth among children and adolescents in the aftermath of sexual abuse', *Counselling Psychology Quarterly*, 25: 73-82.

McFarlane, Alexander C. 1987. 'Family functioning and overprotection following a natural disaster: The longitudinal effects of post-traumatic morbidity', *Australian and New Zealand Journal of Psychiatry*, 21: 210-18.

McFarlane, Alexander C, Michelle Atchison, E Rafalowicz, and P Papay. 1994. 'Physical symptoms in post-traumatic stress disorder', *Journal of psychosomatic research*, 38: 715-26.

McFarlane, Alexander C, and Richard A Bryant. 2007. 'Post-traumatic stress disorder in occupational settings: anticipating and managing the risk', *Occupational medicine*, 57: 404-10.

McFarlane, Alexander C, Penny Williamson, and Christopher A Barton. 2009. 'The impact of traumatic stressors in civilian occupational settings', *Journal of public health policy*, 30: 311-27.

McFarlane, Alexander Cowell. 1988. 'The aetiology of post-traumatic stress disorders following a natural disaster', *The British Journal of Psychiatry*, 152: 116-21.

McFetridge, Mark, Alison Hauenstein Swan, Sarah Heke, Thanos Karatzias, Neil Greenberg, Neil Kitchiner, and Rachel Morley. 2017. 'Guideline for the treatment and planning of services for complex post-traumatic stress disorder in adults'.

McLaughlin, Katie A, Karestan C Koenen, Evelyn J Bromet, Elie G Karam, Howard Liu, Maria Petukhova, Ayelet Meron Ruscio, Nancy A Sampson, Dan J Stein, and Sergio Aguilar-Gaxiola. 2017. 'Childhood adversities and post-traumatic stress disorder: evidence for stress sensitisation in the World Mental Health Surveys', *The British Journal of Psychiatry*, 211: 280-88.

McLay, Robert N, Dennis P Wood, Jennifer A Webb-Murphy, James L Spira, Mark D Wiederhold, Jeffrey M Pyne, and Brenda K Wiederhold. 2011. 'A randomized, controlled trial of virtual reality-graded exposure therapy for post-traumatic stress disorder in active duty service members with combat-related post-traumatic stress disorder', *Cyberpsychology, behavior, and social networking*, 14: 223-29.

McLean, Carmen P, and Edna B Foa. 2011. 'Prolonged exposure therapy for post-traumatic stress disorder: A review of evidence and dissemination', *Expert review of neurotherapeutics*, 11: 1151-63.

Mcleer, Susan V, Esther Deblinger, Marc S Atkins, Edna B Foa, and Diana L Ralphe. 1988. 'Post-traumatic stress disorder in sexually abused children', *Journal of the American Academy of Child & Adolescent Psychiatry*, 27: 650-54.

McMillan, TM. 1996. 'Post-traumatic stress disorder following minor and severe closed head injury: 10 single cases', *Brain Injury*, 10: 749-58.

McNally, Richard J. 2006. 'Cognitive abnormalities in post-traumatic stress disorder', *Trends in cognitive sciences*, 10: 271-77.

Medina-Ortiz, Oscar, Alejandro Oses-Gil, Vivian Vanessa Arenas-Villamizar, Angel Ortega, Milagros Rojas, Mervin Chávez-Castillo, and Franlet Araque-Castellanos. 2023. 'Prevalence and risk factors of posttraumatic stress disorder in COVID-19'.

Meewisse, Marie-Louise, Johannes B Reitsma, Giel-Jan De Vries, Berthold PR Gersons, and Miranda Olff. 2007. 'Cortisol and post-traumatic stress disorder in adults: systematic review and meta-analysis', *The British Journal of Psychiatry*, 191: 387-92.

Mehta, Suneela, and Shanthi N Ameratunga. 2012. 'Prevalence of post‐traumatic stress disorder among children and adolescents who survive road traffic crashes: a systematic review of the international literature', *Journal of paediatrics and child health*, 48: 876-85.

Meiser-Stedman, Richard, Tim Dalgleish, Ed Glucksman, William Yule, and Patrick Smith. 2009. 'Maladaptive cognitive appraisals mediate the evolution of posttraumatic stress reactions: A 6-month follow-up of child and adolescent assault and motor vehicle accident survivors', *Journal of abnormal psychology*, 118: 778.

Meiser-Stedman, Richard, Patrick Smith, Edward Glucksman, William Yule, and Tim Dalgleish. 2007. 'Parent and child agreement for acute stress disorder, post-traumatic stress disorder and other psychopathology in a prospective study of children and adolescents exposed to single-event trauma', *Journal of abnormal child psychology*, 35: 191-201.

Meiser‐Stedman, Richard, Patrick Smith, Richard Bryant, Karen Salmon, William Yule, Tim Dalgleish, and Reginald DV Nixon. 2009. 'Development and validation of the child post‐traumatic cognitions inventory (CPTCI)', *Journal of child psychology and psychiatry*, 50: 432-40.

Mekonnen, Nibretie, Bereket Duko, Melkamu Worku Kercho, and Asres Bedaso. 2022. 'PTSD among road traffic accident survivors in africa: A systematic review and meta-analysis', *Heliyon*, 8.

Melkam, Mamaru, Techilo Tinsae, Fantahun Andualem, and Girum Nakie. 2023. 'Post-traumatic stress disorder and associated factors among adults exposed to stress in Ethiopia: A meta-analysis and systematic review', *SAGE Open Medicine*, 11: 20503121231160884.

Mellman, Thomas A, Daniella David, Victoria Bustamante, Ana I Fins, and Karin Esposito. 2001. 'Predictors of post‐traumatic stress disorder following severe injury', *Depression and Anxiety*, 14: 226-31.

Memarzia, Jessica, Katie Lofthouse, Tim Dalgleish, Adrian Boyle, Anna McKinnon, Clare Dixon, Patrick Smith, and Richard Meiser-Stedman. 2024. 'Predictive models of post-traumatic stress disorder, complex post-traumatic stress disorder, depression, and anxiety in children and adolescents following a single-event trauma', *Psychological medicine*, 54: 3407-16.

Memarzia, Jessica, Jack Walker, and Richard Meiser-Stedman. 2021. 'Psychological peritraumatic risk factors for post-traumatic stress disorder in children and adolescents: A meta-analytic review', *Journal of affective disorders*, 282: 1036-47.

Menage, Janet. 1993. 'Post-traumatic stress disorder in women who have undergone obstetric and/or gynaecological procedures: a consecutive series of 30 cases of PTSD', *Journal of Reproductive and Infant psychology*, 11: 221-28.

Menelas, Bob-Antoine J, Corentin Haidon, Adrien Ecrepont, and Benoit Girard. 2018. 'Use of virtual reality technologies as an Action-Cue Exposure Therapy for truck drivers suffering from Post-Traumatic Stress Disorder', *Entertainment computing*, 24: 1-9.

Merecz, Dorota, Malgorzata Waszkowska, and Agata Wezyk. 2012. 'Psychological consequences of trauma in MVA perpetrators–Relationship between post-traumatic growth, PTSD symptoms and individual characteristics', *Transportation research part F: traffic psychology and behaviour*, 15: 565-74.

Messo, Innocent Nasson. 2013. 'Prevalence of post-traumatic stress disorder in children: the case of the Mbagala bomb blasts in Tanzania', *Journal of health psychology*, 18: 627-37.

Michaels, Andrew J, Claire E Michaels, Christina H Moon, Joshua S Smith, Marc A Zimmerman, Paul A Taheri, and Christopher Peterson. 1999. 'Posttraumatic stress disorder after injury: impact on general health outcome and early risk assessment', *Journal of Trauma and Acute Care Surgery*, 47: 460-67.

Mikkelsen, Eva Gemz⊘ e, and Stale Einarsen. 2002. 'Basic assumptions and symptoms of post-traumatic stress among victims of bullying at work', *European journal of work and organizational psychology*, 11: 87-111.

Mikkelsen, Mark E, Jason D Christie, Paul N Lanken, Rosette C Biester, B Taylor Thompson, Scarlett L Bellamy, A Russell Localio, Ejigayehu Demissie, Ramona O Hopkins, and Derek C Angus. 2012. 'The adult respiratory distress syndrome cognitive outcomes study: long-term neuropsychological function in survivors of acute lung injury', *American journal of respiratory and critical care medicine*, 185: 1307-15.

Miller, Mark W, and Kelly M Harrington. 2011. 'Personality factors in resilience to traumatic stress', *Resilience and mental health: Challenges across the lifespan*: 56-75.

Miniati, Mario, Laura Palagini, Danila Caruso, Mauro Mauri, Donatella Marazziti, and Liliana Dell’Osso. 2021. 'Post-traumatic stress disorder in train crash survivors in Italy: The role of mood spectrum dysregulations and intrusiveness', *CNS spectrums*, 26: 71-76.

Miragoli, Sarah, Elena Camisasca, and Paola Di Blasio. 2017. 'Narrative fragmentation in child sexual abuse: The role of age and post-traumatic stress disorder', *Child abuse & neglect*, 73: 106-14.

Mirza, KAH, BR Bhadrinath, Ian M Goodyer, and Carol Gilmour. 1998. 'Post-traumatic stress disorder in children and adolescents following road traffic accidents', *The British Journal of Psychiatry*, 172: 443-47.

Mitani, Satoko, Masatoshi Fujita, Keiji Nakata, and Taro Shirakawa. 2006. 'Impact of post-traumatic stress disorder and job-related stress on burnout: A study of fire service workers', *The Journal of emergency medicine*, 31: 7-11.

Mitchell, Jeffrey T, and George S Everly Jr. 1995. 'Critical incident stress debriefing (CISD) and the prevention of work-related traumatic stress among high risk occupational groups.' in, *Psychotraumatology: Key papers and core concepts in post-traumatic stress* (Springer).

Moeller-Bertram, Tobias, John Keltner, and Irina A Strigo. 2012. 'Pain and post traumatic stress disorder–review of clinical and experimental evidence', *Neuropharmacology*, 62: 586-97.

Mojtabavi, Helia, Amene Saghazadeh, Leigh van den Heuvel, Joana Bucker, and Nima Rezaei. 2020. 'Peripheral blood levels of brain-derived neurotrophic factor in patients with post-traumatic stress disorder (PTSD): A systematic review and meta-analysis', *PLoS One*, 15: e0241928.

Moor, Avigail, and Moshe Farchi. 2011. 'Is rape-related self blame distinct from other post traumatic attributions of blame? A comparison of severity and implications for treatment', *Women & Therapy*, 34: 447-60.

Morina, Nexhmedin, Hendryk F Böhme, Dean Ajdukovic, Marija Bogic, Tanja Franciskovic, Gian M Galeazzi, Abdulah Kucukalic, Dusica Lecic-Tosevski, Mihajlo Popovski, and Matthias Schützwohl. 2010. 'The structure of post-traumatic stress symptoms in survivors of war: Confirmatory factor analyses of the Impact of Event Scale—Revised', *Journal of anxiety disorders*, 24: 606-11.

Morina, Nexhmedin, Jelte M Wicherts, Jakob Lobbrecht, and Stefan Priebe. 2014. 'Remission from post-traumatic stress disorder in adults: a systematic review and meta-analysis of long term outcome studies', *Clinical psychology review*, 34: 249-55.

Moulaert, Véronique RMP, Esther M Wachelder, Jeanine Verbunt, Derick T Wade, and Caroline M van Heugten. 2010. 'Determinants of quality of life in survivors of cardiac arrest', *Journal of rehabilitation medicine*, 42: 553-58.

Mrdjenovich, Adam J. 2018. 'Post-traumatic stress disorder and traffic accidents: Differential diagnosis and comorbid conditions', *International Journal of Child & Adolescent Health*, 11.

Muldoon, Orla T, and Robert D Lowe. 2012. 'Social identity, groups, and post‐traumatic stress disorder', *Political Psychology*, 33: 259-73.

Murdoch, Maureen, Melissa A Polusny, James Hodges, and Diane Cowper. 2006. 'The association between in-service sexual harassment and post-traumatic stress disorder among Department of Veterans Affairs disability applicants', *Military medicine*, 171: 166-73.

Murphy, Dominic, E Palmer, R Lock, and W Busuttil. 2017. 'Post-traumatic growth among the UK veterans following treatment for post-traumatic stress disorder', *BMJ Military Health*, 163: 140-45.

Murray, Hannah, and Anke Ehlers. 2021. 'Cognitive therapy for moral injury in post-traumatic stress disorder', *The Cognitive Behaviour Therapist*, 14: e8.

Murray, James, and James Murray. 1997. 'The role of dissociation in the development and maintenance of post-traumatic stress disorder', University of Oxford.

Naim, R, I Wald, A Lior, DS Pine, Nathan A Fox, G Sheppes, P Halpern, and Y Bar-Haim. 2014. 'Perturbed threat monitoring following a traumatic event predicts risk for post-traumatic stress disorder', *Psychological medicine*, 44: 2077-84.

Nakajima, Satomi, Ito Masaya, Shirai Akemi, and Konishi Takako. 2012. 'Complicated grief in those bereaved by violent death: the effects of post-traumatic stress disorder on complicated grief', *Dialogues in clinical neuroscience*, 14: 210-14.

Nakell, Linda. 2007. 'Adult post-traumatic stress disorder: screening and treating in primary care', *Primary Care: Clinics in Office Practice*, 34: 593-610.

Neal, Leigh Anthony, Walter Busuttil, John Rollins, Ronald Herepath, Paul Strike, and Gordon Turnbull. 1994. 'Convergent validity of measures of post-traumatic stress disorder in a mixed military and civilian population', *Journal of Traumatic stress*, 7: 447-55.

Neria, Yuval, Arijit Nandi, and Sandro Galea. 2008. 'Post-traumatic stress disorder following disasters: a systematic review', *Psychological medicine*, 38: 467-80.

Neugebauer, Richard, Prudence W Fisher, J Blake Turner, Saori Yamabe, Julia A Sarsfield, and Tasha Stehling-Ariza. 2009. 'Post-traumatic stress reactions among Rwandan children and adolescents in the early aftermath of genocide', *International journal of epidemiology*, 38: 1033-45.

Newman, Elana, Roger Simpson, and David Handschuh. 2003. 'Trauma exposure and post‐traumatic stress disorder among photojournalists', *Visual Communication Quarterly*, 10: 4-13.

Neylan, Thomas C, Ronald C Kessler, Kerry J Ressler, Gari Clifford, Francesca L Beaudoin, Xinming An, Jennifer S Stevens, Donglin Zeng, Sarah D Linnstaedt, and Laura T Germine. 2021. 'Prior sleep problems and adverse post-traumatic neuropsychiatric sequelae of motor vehicle collision in the AURORA study', *Sleep*, 44: zsaa200.

Nicholl, Catherine, and Andrew Thompson. 2004. 'The psychological treatment of Post Traumatic Stress Disorder (PTSD) in adult refugees: A review of the current state of psychological therapies', *Journal of Mental Health*, 13: 351-62.

Nicholls, Karen, and Susan Ayers. 2007. 'Childbirth‐related post‐traumatic stress disorder in couples: A qualitative study', *British Journal of Health Psychology*, 12: 491-509.

Nichols, Beverly L, and Ruth Czirr. 1986. '24/Post-Traumatic Stress Disorder: Hidden Syndrome in Elders', *Clinical Gerontologist*, 5: 417-33.

Nickerson, A, M Creamer, D Forbes, AC McFarlane, ML O'donnell, D Silove, Z Steel, K Felmingham, D Hadzi-Pavlovic, and RA Bryant. 2017. 'The longitudinal relationship between post-traumatic stress disorder and perceived social support in survivors of traumatic injury', *Psychological medicine*, 47: 115-26.

Nielsen, Morten Birkeland, Tone Tangen, Thormod Idsoe, Stig Berge Matthiesen, and Nils Magerøy. 2015. 'Post-traumatic stress disorder as a consequence of bullying at work and at school. A literature review and meta-analysis', *Aggression and violent behavior*, 21: 17-24.

Nishi, Daisuke, Masato Usuki, and Yutaka Matsuoka. 2012. 'Peritraumatic Distress in Accident Survivors: An Indicator for Posttraumatic Stress, Depressive and Anxiety Symptoms, and Posttraumatic Growth', *Post Traumatic Stress Disorders in a Global Context: InTech*: 97-112.

Norberg, Annika Lindahl, Ulrika Pöder, and Louise von Essen. 2011. 'Early avoidance of disease-and treatment-related distress predicts post-traumatic stress in parents of children with cancer', *European Journal of Oncology Nursing*, 15: 80-84.

Norman, Sonya B, Murray B Stein, JE Dimsdale, and David B Hoyt. 2008. 'Pain in the aftermath of trauma is a risk factor for post-traumatic stress disorder', *Psychological medicine*, 38: 533-42.

Norris, Fran H. 1990. 'Screening for traumatic stress: a scale for use in the general population 1', *Journal of Applied Social Psychology*, 20: 1704-15.

Nutt, David J, JRT Davidson, Joseph Zohar, and J Zohar. 2009. *Post Traumatic Stress Disorders* (Informa Healthcare).

O'Donohue, William, and Ann Elliott. 1992. 'The current status of post‐traumatic stress disorder as a diagnostic category: Problems and proposals', *Journal of Traumatic stress*, 5: 421-39.

O'Shea, Brian. 2001. 'Post-traumatic stress disorder: A review for the general psychiatrist', *International journal of psychiatry in clinical practice*, 5: 11-18.

Ochberg, Frank M. 2013. 'Post-traumatic therapy and victims of violence.' in, *Post-traumatic therapy and victims of violence* (Routledge).

Oflaz, Fahriye, Sevgi Hatipoğlu, and Hamdullah Aydin. 2008. 'Effectiveness of psychoeducation intervention on post‐traumatic stress disorder and coping styles of earthquake survivors', *Journal of Clinical Nursing*, 17: 677-87.

Ohry, A, J Rattok, and Z Solomon. 1996. 'Post-traumatic stress disorder in brain injury patients', *Brain Injury*, 10: 687-96.

Olliac, Bertrand, Philippe Birmes, Eric Bui, Charlotte Allenou, Alain Brunet, Isabelle Claudet, Jérôme Sales de Gauzy, Hélène Grandjean, and Jean-Philippe Raynaud. 2014. 'Validation of the French version of the child post-traumatic stress reaction index: psychometric properties in French speaking school-aged children', *PLoS One*, 9: e112603.

Onyencho, VC, B Omeiza, and MA Wakil. 2014. 'Post-traumatic stress disorder and psychological well-being among University of Maiduguri students', *IFE PsychologIA: An International Journal*, 22: 195-201.

Oquendo, MA, G Echavarria, HC Galfalvy, MF Grunebaum, A Burke, A Barrera, TB Cooper, KM Malone, and J John Mann. 2003. 'Lower cortisol levels in depressed patients with comorbid post-traumatic stress disorder', *Neuropsychopharmacology*, 28: 591-98.

Orr, Scott P, Linda J Metzger, and Roger K Pitman. 2002. 'Psychophysiology of post-traumatic stress disorder', *Psychiatric Clinics*, 25: 271-93.

Osenbach, Janyce E, Charles Lewis, Barry Rosenfeld, Joan Russo, Leah M Ingraham, Roselyn Peterson, Jin Wang, and Douglas F Zatzick. 2014. 'Exploring the longitudinal trajectories of posttraumatic stress disorder in injured trauma survivors', *Psychiatry: Interpersonal and Biological Processes*, 77: 386-97.

Ostrowski, Sarah A, and Douglas L Delahanty. 2014. 'Prospects for the pharmacological prevention of post-traumatic stress in vulnerable individuals', *CNS drugs*, 28: 195-203.

Otis, John D, Terence M Keane, and Robert D Kerns. 2003. 'An examination of the relationship between chronic pain and post-traumatic stress disorder', *Journal of Rehabilitation Research & Development*, 40.

Pagel, JF. 2021. *Post-Traumatic Stress Disorder* (Springer).

Palm, Kathleen M, Melissa A Polusny, and Victoria M Follette. 2004. 'Vicarious traumatization: Potential hazards and interventions for disaster and trauma workers', *Prehospital and disaster medicine*, 19: 73-78.

Palyo, Sarah A, and J Gayle Beck. 2005. 'Post-traumatic stress disorder symptoms, pain, and perceived life control: Associations with psychosocial and physical functioning', *Pain*, 117: 121-27.

Pan, Xiongfeng, Atipatsa C Kaminga, Shi Wu Wen, and Aizhong Liu. 2018. 'Catecholamines in post-traumatic stress disorder: a systematic review and meta-analysis', *Frontiers in molecular neuroscience*, 11: 450.

Pan, Xiongfeng, Zhipeng Wang, Xiaoli Wu, Shi Wu Wen, and Aizhong Liu. 2018. 'Salivary cortisol in post-traumatic stress disorder: a systematic review and meta-analysis', *BMC psychiatry*, 18: 1-10.

Panagioti, Maria, Patricia Gooding, and Nicholas Tarrier. 2009. 'Post-traumatic stress disorder and suicidal behavior: A narrative review', *Clinical psychology review*, 29: 471-82.

Papadakaki, Maria, Ottavia Eleonora Ferraro, Chiara Orsi, Dietmar Otte, Georgia Tzamalouka, Marco Von-der-Geest, Timo Lajunen, Türker Özkan, Anna Morandi, and Markos Sarris. 2017. 'Psychological distress and physical disability in patients sustaining severe injuries in road traffic crashes: Results from a one-year cohort study from three European countries', *Injury*, 48: 297-306.

Paparrigopoulos, Thomas, Antigone Melissaki, Elias Tzavellas, Dimitris Karaiskos, Ioannis Ilias, and Nikolaos Kokras. 2014. 'Increased co-morbidity of depression and post-traumatic stress disorder symptoms and common risk factors in intensive care unit survivors: a two-year follow-up study', *International journal of psychiatry in clinical practice*, 18: 25-31.

Parikh, Darshit, Paolo De Ieso, Gail Garvey, Thanuja Thachil, Ramya Ramamoorthi, Michael Penniment, and Rama Jayaraj. 2015. 'Post-traumatic stress disorder and post-traumatic growth in breast cancer patients-a systematic review', *Asian Pacific Journal of Cancer Prevention*, 16: 641-46.

Park, Hye Yoon, Wan Beom Park, So Hee Lee, Jeong Lan Kim, Jung Jae Lee, Haewoo Lee, and Hyoung-Shik Shin. 2020. 'Posttraumatic stress disorder and depression of survivors 12 months after the outbreak of Middle East respiratory syndrome in South Korea', *BMC public health*, 20: 1-9.

Parker, Ann M, Thiti Sricharoenchai, Sandeep Raparla, Kyle W Schneck, O Joseph Bienvenu, and Dale M Needham. 2015. 'Posttraumatic stress disorder in critical illness survivors: a metaanalysis', *Critical care medicine*, 43: 1121-29.

Parvaresh, Nooshin, and Ali Bahramnezhad. 2009. 'Post-traumatic stress disorder in bam-survived students who immigrated to Kerman, four months after the earthquake'.

Patel, Mayur B, James C Jackson, Alessandro Morandi, Timothy D Girard, Christopher G Hughes, Jennifer L Thompson, Amy L Kiehl, Mark R Elstad, Mitzi L Wasserstein, and Richard B Goodman. 2016. 'Incidence and risk factors for intensive care unit–related post-traumatic stress disorder in veterans and civilians', *American journal of respiratory and critical care medicine*, 193: 1373-81.

Patterson, David R, Lynn Carrigan, Kent A Questad, and Ross Robinson. 1990. 'Post-traumatic stress disorder in hospitalized patients with burn injuries', *The Journal of Burn Care & Rehabilitation*, 11: 181-84.

Paunovic, Nenad. 1998. 'Cognitive factors in the maintenance of PTSD', *Behaviour Therapy*, 27: 167-78.

Paxson, Christina, Elizabeth Fussell, Jean Rhodes, and Mary Waters. 2012. 'Five years later: Recovery from post traumatic stress and psychological distress among low-income mothers affected by Hurricane Katrina', *Social Science & Medicine*, 74: 150-57.

Pelcovitz, David, Sandra Kaplan, Barbara Goldenberg, Fran Mandel, Julie Lehane, and James Guarrera. 1994. 'Post-traumatic stress disorder in physically abused adolescents', *Journal of the American Academy of Child & Adolescent Psychiatry*, 33: 305-12.

Pelissier, Carole, Emmanuel Fort, Luc Fontana, Barbara Charbotel, and Martine Hours. 2017. 'Factors associated with non-return to work in the severely injured victims 3 years after a road accident: A prospective study', *Accident Analysis & Prevention*, 106: 411-19.

Perrin, Marc, Caroline L Vandeleur, Enrique Castelao, Stéphane Rothen, Jennifer Glaus, Peter Vollenweider, and Martin Preisig. 2014. 'Determinants of the development of post-traumatic stress disorder, in the general population', *Social psychiatry and psychiatric epidemiology*, 49: 447-57.

Perrin, Sean, Patrick Smith, and William Yule. 2000. 'Practitioner review: The assessment and treatment of post-traumatic stress disorder in children and adolescents', *The Journal of Child Psychology and Psychiatry and Allied Disciplines*, 41: 277-89.

Perry, Bruce D. 1994. 'Neurobiological sequelae of childhood trauma: Post-traumatic stress disorders in children', *Catecholamine function in post traumatic stress disorder: Emerging concepts*: 233-55.

Pervanidou, P. 2008. 'Biology of post‐traumatic stress disorder in childhood and adolescence', *Journal of neuroendocrinology*, 20: 632-38.

Pervanidou, Panagiota, and George P Chrousos. 2010. 'Neuroendocrinology of post-traumatic stress disorder', *Progress in brain research*, 182: 149-60.

Peterson, Kirtland C, Maurice F Prout, and Robert A Schwarz. 2013. *Post-traumatic stress disorder: A clinician’s guide* (Springer Science & Business Media).

Petrowski, Katja, Susann Wichmann, Jaroslav Pyrc, Susann Steudte-Schmiedgen, and Clemens Kirschbaum. 2020. 'Hair cortisol predicts avoidance behavior and depressiveness after first-time and single-event trauma exposure in motor vehicle crash victims', *Stress*, 23: 567-76.

Pierce, Zachary P, Emily R Johnson, Isabelle A Kim, Brianna E Lear, A Michaela Mast, and Jessica M Black. 2023. 'Therapeutic interventions impact brain function and promote post-traumatic growth in adults living with post-traumatic stress disorder: A systematic review and meta-analysis of functional magnetic resonance imaging studies', *Frontiers in psychology*, 14: 1074972.

Pillar, Giora, Atul Malhotra, and Peretz Lavie. 2000. 'Post-traumatic stress disorder and sleep—what a nightmare!', *Sleep medicine reviews*, 4: 183-200.

Pires, Tânia Sofia Fernandes, and Ângela da Costa Maia. 2013. 'Posttraumatic stress disorder among serious motor vehicle accident victims: analysis of predictors', *Archives of Clinical Psychiatry (São Paulo)*, 40: 211-14.

Pitman, Roger K, Ann M Rasmusson, Karestan C Koenen, Lisa M Shin, Scott P Orr, Mark W Gilbertson, Mohammed R Milad, and Israel Liberzon. 2012. 'Biological studies of post-traumatic stress disorder', *nature Reviews neuroscience*, 13: 769-87.

Powell, Trevor, Abigail Ekin-Wood, and Christine Collin. 2007. 'Post-traumatic growth after head injury: A long-term follow-up', *Brain Injury*, 21: 31-38.

PRAKASAM, ARUL, ANN JOSEPH, NANISHA ZACHARIAH, and SHARON JOY. 2020. 'Assess the impact of post traumatic stress disorder in road traffic accident survivors in Erode district, Tamil nadu', *International Journal of Pharmaceutical Research (09752366)*, 12.

Priebe, Stefan, Iolanda Grappasonni, Massimo Mari, Michael Dewey, Fabio Petrelli, and Ana Costa. 2009. 'Posttraumatic stress disorder six months after an earthquake: findings from a community sample in a rural region in Italy', *Social psychiatry and psychiatric epidemiology*, 44: 393-97.

Pynoos, Robert S, Armen Goenjian, Madeline Tashjian, Meline Karakashian, Raffi Manjikian, Gagik Manoukian, Alan M Steinberg, and Lynn A Fairbanks. 1993. 'Post-traumatic stress reactions in children after the 1988 Armenian earthquake', *The British Journal of Psychiatry*, 163: 239-47.

Qi, Wei, Martin Gevonden, and Arieh Shalev. 2016. 'Prevention of post-traumatic stress disorder after trauma: current evidence and future directions', *Current psychiatry reports*, 18: 1-11.

Qiu, Dan, Yilu Li, Ling Li, Jun He, Feiyun Ouyang, and Shuiyuan Xiao. 2021. 'Prevalence of post-traumatic stress symptoms among people influenced by coronavirus disease 2019 outbreak: A meta-analysis', *European Psychiatry*, 64: e30.

Ratnani, Devendra. 'Study of Post Traumatic Stress Disorder Among Survivors of Road Traffic Accident', *International journal of health sciences*, 6: 3762-67.

Ravn, Sophie Lykkegaard, Jan Hartvigsen, Maj Hansen, Michele Sterling, and Tonny Elmose Andersen. 2018. 'Do post-traumatic pain and post-traumatic stress symptomatology mutually maintain each other? A systematic review of cross-lagged studies', *Pain*, 159: 2159-69.

Realmuto, George M, Ann Masten, Linda Flies Carole, Jon Hubbard, Andrea Groteluschen, and Bunkhean Chhun. 1992. 'Adolescent survivors of massive childhood trauma in Cambodia: Life events and current symptoms', *Journal of Traumatic stress*, 5: 589-99.

Regel, Stephen. 2007. 'Post-trauma support in the workplace: the current status and practice of critical incident stress management (CISM) and psychological debriefing (PD) within organizations in the UK', *Occupational medicine*, 57: 411-16.

Resick, Patricia A, and Mark W Miller. 2009. 'Posttraumatic stress disorder: anxiety or traumatic stress disorder?', *Journal of Traumatic stress*, 22: 384-90.

Ressler, Kerry J, Sabina Berretta, Vadim Y Bolshakov, Isabelle M Rosso, Edward G Meloni, Scott L Rauch, and William A Carlezon Jr. 2022. 'Post-traumatic stress disorder: clinical and translational neuroscience from cells to circuits', *Nature Reviews Neurology*, 18: 273-88.

Reynolds, Kristin, Robert H Pietrzak, Corey S Mackenzie, Kee Lee Chou, and Jitender Sareen. 2016. 'Post-traumatic stress disorder across the adult lifespan: findings from a nationally representative survey', *The American Journal of Geriatric Psychiatry*, 24: 81-93.

Reynolds, Martina, and Chris R Brewin. 1998. 'Intrusive cognitions, coping strategies and emotional responses in depression, post-traumatic stress disorder and a non-clinical population', *Behaviour research and therapy*, 36: 135-47.

Reynolds, Martina, Gillian Mezey, Murray Chapman, Mike Wheeler, Colin Drummond, and Alex Baldacchino. 2005. 'Co-morbid post-traumatic stress disorder in a substance misusing clinical population', *Drug and alcohol dependence*, 77: 251-58.

Richardson, Lisa K, B Christopher Frueh, and Ronald Acierno. 2010. 'Prevalence estimates of combat-related post-traumatic stress disorder: critical review', *Australian & New Zealand Journal of Psychiatry*, 44: 4-19.

Riggs, David S, Constance V Dancu, Beth S Gershuny, Deborah Greenberg, and Edna B Foa. 1992. 'Anger and post‐traumatic stress disorder in female crime victims', *Journal of Traumatic stress*, 5: 613-25.

Riggs, David S, Barbara O Rothbaum, and Edna B Foa. 1995. 'A prospective examination of symptoms of posttraumatic stress disorder in victims of nonsexual assault', *Journal of Interpersonal Violence*, 10: 201-14.

Righy, Cassia, Regis Goulart Rosa, Rodrigo Teixeira Amancio da Silva, Renata Kochhann, Celina Borges Migliavaca, Caroline Cabral Robinson, Stefania Pigatto Teche, Cassiano Teixeira, Fernando Augusto Bozza, and Maicon Falavigna. 2019. 'Prevalence of post-traumatic stress disorder symptoms in adult critical care survivors: a systematic review and meta-analysis', *Critical care*, 23: 1-13.

Rissanen, Ritva, Hans-Yngve Berg, and Marie Hasselberg. 2017. 'Quality of life following road traffic injury: A systematic literature review', *Accident Analysis & Prevention*, 108: 308-20.

Rivera-Vélez, Giselle M, Maribella González-Viruet, Alfonso Martínez-Taboas, and Deborah Pérez-Mojica. 2014. 'Post-traumatic stress disorder, dissociation, and neuropsychological performance in Latina victims of childhood sexual abuse', *Journal of child sexual abuse*, 23: 55-73.

Roberts, Andrea L, Stephen E Gilman, Joshua Breslau, Naomi Breslau, and Karestan C Koenen. 2011. 'Race/ethnic differences in exposure to traumatic events, development of post-traumatic stress disorder, and treatment-seeking for post-traumatic stress disorder in the United States', *Psychological medicine*, 41: 71-83.

Rodríguez‐Muñoz, Alfredo, Bernardo Moreno‐Jiménez, Ana Isabel Sanz Vergel, and Eva Garrosa Hernández. 2010. 'Post‐Traumatic symptoms among victims of workplace bullying: Exploring gender differences and shattered assumptions', *Journal of Applied Social Psychology*, 40: 2616-35.

Rogers, Jeffrey M, and Christina A Read. 2007. 'Psychiatric comorbidity following traumatic brain injury', *Brain Injury*, 21: 1321-33.

Rogers, Mark A, Hidenori Yamasue, Osamu Abe, Haruyasu Yamada, Toshiyuki Ohtani, Akira Iwanami, Shigeki Aoki, Nobumasa Kato, and Kiyoto Kasai. 2009. 'Smaller amygdala volume and reduced anterior cingulate gray matter density associated with history of post-traumatic stress disorder', *Psychiatry Research: Neuroimaging*, 174: 210-16.

Roop, Kishen. 2006. 'Post-traumatic stress disorder in the critically ill patients', *Indian Journal of Critical Care Medicine*, 10: 163-66.

Rose, Suzanna, Chris R Brewin, Ben Andrews, and M Kirk. 1999. 'A randomized controlled trial of individual psychological debriefing for victims of violent crime', *Psychological medicine*, 29: 793-99.

Rose, Suzanna C, Jonathan Bisson, Rachel Churchill, Simon Wessely, and Cochrane Common Mental Disorders Group. 1996. 'Psychological debriefing for preventing post traumatic stress disorder (PTSD)', *Cochrane database of systematic reviews*, 2010.

Roth, Randy S, Michael E Geisser, and Rachel Bates. 2008. 'The relation of post-traumatic stress symptoms to depression and pain in patients with accident-related chronic pain', *The Journal of Pain*, 9: 588-96.

Roussis, Panos, and Adrian Wells. 2006. 'Post-traumatic stress symptoms: Tests of relationships with thought control strategies and beliefs as predicted by the metacognitive model', *Personality and Individual Differences*, 40: 111-22.

Rowan, Anderson B, and David W Foy. 1993. 'Post-traumatic stress disorder in child sexual abuse survivors: A literature review', *Journal of Traumatic stress*, 6: 3-20.

Ruini, Chiara, Francesca Vescovelli, and Elisa Albieri. 2013. 'Post-traumatic growth in breast cancer survivors: new insights into its relationships with well-being and distress', *Journal of clinical psychology in medical settings*, 20: 383-91.

Ryan, Joanne, Isabelle Chaudieu, Marie-Laure Ancelin, and Richard Saffery. 2016. 'Biological underpinnings of trauma and post-traumatic stress disorder: focusing on genetics and epigenetics', *Epigenomics*, 8: 1553-69.

Sabahi, Zahra, Parham Hasani, Hanieh Salehi-Pourmehr, Rasa Beheshti, and Homayoun Sadeghi-Bazargani. 2024. 'What are the predictors of post-traumatic stress disorder among road traffic accident survivors: a systematic review', *The Journal of Nervous and Mental Disease*, 212: 104-16.

Sakuma, Atsushi, Yoko Takahashi, Ikki Ueda, Hirotoshi Sato, Masahiro Katsura, Mikika Abe, Ayami Nagao, Yuriko Suzuki, Masako Kakizaki, and Ichiro Tsuji. 2015. 'Post-traumatic stress disorder and depression prevalence and associated risk factors among local disaster relief and reconstruction workers fourteen months after the Great East Japan Earthquake: a cross-sectional study', *BMC psychiatry*, 15: 1-13.

Salcioglu, Ebru, Metin Basoglu, and Maria Livanou. 2007. 'Post‐traumatic stress disorder and comorbid depression among survivors of the 1999 earthquake in Turkey', *Disasters*, 31: 115-29.

Samuelson, Kristin W. 2011. 'Post-traumatic stress disorder and declarative memory functioning: a review', *Dialogues in clinical neuroscience*, 13: 346-51.

Saunders, Benjamin E, Catalina Mandoki Arata, and Dean G Kilpatrick. 1990. 'Development of a crime-related post-traumatic stress disorder scale for women within the Symptom Checklist-90-Revised', *Journal of Traumatic stress*, 3: 439-48.

Sbordone, Robert J. 1999. 'Post-traumatic stress disorder: An overview and its relationship to closed head injuries', *NeuroRehabilitation*, 13: 69-78.

Schäfer, SK, N Becker, L King, A Horsch, and T Michael. 2019. 'The relationship between sense of coherence and post-traumatic stress: a meta-analysis', *European Journal of Psychotraumatology*, 10: 1562839.

Schindel-Allon, I, IM Aderka, G Shahar, M Stein, and E Gilboa-Schechtman. 2010. 'Longitudinal associations between post-traumatic distress and depressive symptoms following a traumatic event: a test of three models', *Psychological medicine*, 40: 1669-78.

Schnyder, Ulrich, Hanspeter Moergeli, Richard Klaghofer, and Claus Buddeberg. 2001. 'Incidence and prediction of posttraumatic stress disorder symptoms in severely injured accident victims', *American Journal of Psychiatry*, 158: 594-99.

Schnyder, Ulrich, Hanspeter Moergeli, Otmar Trentz, Richard Klaghofer, and Claus Buddeberg. 2001. 'Prediction of psychiatric morbidity in severely injured accident victims at one-year follow-up', *American journal of respiratory and critical care medicine*, 164: 653-56.

Schnyder, Ulrich, Lutz Wittmann, Josefina Friedrich-Perez, Urs Hepp, and Hanspeter Moergeli. 2008. 'Posttraumatic stress disorder following accidental injury: rule or exception in Switzerland?', *Psychotherapy and psychosomatics*, 77: 111-18.

Schöner, Johanna, Andreas Heinz, Matthias Endres, Karen Gertz, and Golo Kronenberg. 2017. 'Post‐traumatic stress disorder and beyond: an overview of rodent stress models', *Journal of cellular and molecular medicine*, 21: 2248-56.

Schreiber, S, and T Galai-Gat. 1993. 'Uncontrolled pain following physical injury as the core-trauma in post-traumatic stress disorder', *Pain*, 54: 107-10.

Schubert, Christine F, Ulrike Schmidt, and Rita Rosner. 2016. 'Posttraumatic growth in populations with posttraumatic stress disorder—A systematic review on growth‐related psychological constructs and biological variables', *Clinical psychology & psychotherapy*, 23: 469-86.

Schultebraucks, Katharina, Arieh Y Shalev, Vasiliki Michopoulos, Corita R Grudzen, Soo-Min Shin, Jennifer S Stevens, Jessica L Maples-Keller, Tanja Jovanovic, George A Bonanno, and Barbara O Rothbaum. 2020. 'A validated predictive algorithm of post-traumatic stress course following emergency department admission after a traumatic stressor', *Nature medicine*, 26: 1084-88.

Schuster, Michelle, and Patricia A Dwyer. 2020. 'Post‐traumatic stress disorder in nurses: An integrative review', *Journal of Clinical Nursing*, 29: 2769-87.

Schwarz, Eitan D, and Bruce D Perry. 1994. 'The post-traumatic response in children and adolescents', *Psychiatric Clinics*, 17: 311-26.

Ścigała, Dawid Konrad, and Elżbieta Zdankiewicz-Ścigała. 2019. 'The role in road traffic accident and anxiety as moderators attention biases in modified emotional stroop test', *Frontiers in psychology*, 10: 1575.

Seedat, Soraya, C Nyamai, F Njenga, Bc Vythilingum, and Dan J Stein. 2004. 'Trauma exposure and post-traumatic stress symptoms in urban African schools: Survey in Cape Town and Nairobi', *The British Journal of Psychiatry*, 184: 169-75.

Seedat, Soraya, Dan J Stein, and Paul D Carey. 2005. 'Post-traumatic stress disorder in women: epidemiological and treatment issues', *CNS drugs*, 19: 411-27.

Segman, RH, N Shefi, T Goltser-Dubner, N Friedman, N Kaminski, and AY Shalev. 2005. 'Peripheral blood mononuclear cell gene expression profiles identify emergent post-traumatic stress disorder among trauma survivors', *Molecular psychiatry*, 10: 500-13.

Sembi, Sundeep, Nicholas Tarrier, Paul O'Neill, Alistair Burns, and Brian Faragher. 1998. 'Does post‐traumatic stress disorder occur after stroke: a preliminary study', *International Journal of Geriatric Psychiatry*, 13: 315-22.

Seng, Julia S, Lisa Kane Low, Mickey Sperlich, David L Ronis, and Israel Liberzon. 2011. 'Post‐traumatic stress disorder, child abuse history, birthweight and gestational age: a prospective cohort study', *BJOG: An International Journal of Obstetrics & Gynaecology*, 118: 1329-39.

Sepahvand, Hossein, Mina Mokhtari Hashtjini, Mahmood Salesi, Hedayat Sahraei, and Gila Pirzad Jahromi. 2019. 'Prevalence of post-traumatic stress disorder (PTSD) in Iranian population following disasters and wars: A systematic review and meta-analysis', *Iranian Journal of Psychiatry and Behavioral Sciences*, 13.

Serrano-Ibanez, Elena R, Tania Corras, Mirtha Del Prado, Javier Diz, and Carmen Varela. 2023. 'Psychological variables associated with post-traumatic stress disorder in firefighters: A systematic review', *Trauma, Violence, & Abuse*, 24: 2049-66.

SHaH, Hetal JitendrabHai, SaPna Patel, and SandeeP Patel. 2024. 'Prevalence of Post-traumatic Stress Disorder Symptoms among Road Traffic Accident Victims: A Cross-sectional Study from Gujarat, India', *Journal of Clinical & Diagnostic Research*, 18.

Shakespeare-Finch, Jane E, SG Smith, Kathryn M Gow, Gary Embelton, and Leith Baird. 2003. 'The prevalence of post-traumatic growth in emergency ambulance personnel', *Traumatology*, 9: 58-71.

Shalev, Arieh, Israel Liberzon, and Charles Marmar. 2017. 'Post-traumatic stress disorder', *New England journal of medicine*, 376: 2459-69.

Shalev, Arieh Y. 2009. 'Posttraumatic stress disorder and stress-related disorders', *Psychiatric Clinics*, 32: 687-704.

Shalev, Arieh Y, Tuvia Peri, Laura Canetti, and Shaul Schreiber. 1996. 'Predictors of PTSD in injured trauma survivors: A prospective study', *American Journal of Psychiatry*, 153: 219-25.

Shalev, Arieh Y, Elizabeth J Videlock, Tamar Peleg, Ronen Segman, Roger K Pitman, and Rachel Yehuda. 2008. 'Stress hormones and post-traumatic stress disorder in civilian trauma victims: a longitudinal study. Part I: HPA axis responses', *International Journal of Neuropsychopharmacology*, 11: 365-72.

Shannon, Mitsuko P, Christopher J Lonigan, AJ Finch Jr, and Charlotte M Taylor. 1994. 'Children exposed to disaster: I. Epidemiology of post-traumatic symptoms and symptom profiles', *Journal of the American Academy of Child & Adolescent Psychiatry*, 33: 80-93.

Shea, Alison, Christine Walsh, Harriet MacMillan, and Meir Steiner. 2005. 'Child maltreatment and HPA axis dysregulation: relationship to major depressive disorder and post traumatic stress disorder in females', *Psychoneuroendocrinology*, 30: 162-78.

Sheikhbardsiri, Hojat, Mahdieh Sarhadi, Asma Abdollahyar, Majid Dastres, RABARI AKBAR SHEIKH, and Mohsen Aminizadeh. 2015. 'The relationship between personality traits and post-traumatic stress disorder among EMS personnel and hospital emergency staffs'.

Shen, Xin, Shijiao Yan, Heng Jiang, Hui Cao, Rowan Dowling, Jing Feng, Zihui Lei, Crystal Jingru Li, Xiaotong Han, and Chuanzhu Lv. 2021. 'Post-traumatic stress disorder and associated factors 1 year after the beginning of the COVID-19 pandemic among Chinese residents', *Frontiers in psychiatry*, 12: 766127.

Sherin, Jonathan E, and Charles B Nemeroff. 2011. 'Post-traumatic stress disorder: the neurobiological impact of psychological trauma', *Dialogues in clinical neuroscience*, 13: 263-78.

Shrestha, Roshana. 2015. 'Post-traumatic stress disorder among medical personnel after Nepal earthquake, 2015'.

Sijbrandij, Marit, Johannes B Reitsma, Neil P Roberts, Iris M Engelhard, Miranda Olff, Liza P Sonneveld, and Jonathan I Bisson. 2013. 'Self-report screening instruments for post-traumatic stress disorder (PTSD) in survivors of traumatic experiences', *Cochrane database of systematic reviews*, 2013: CD010575.

Silove, Derrick, Zachary Steel, Patrick McGorry, Vanessa Miles, and Juliette Drobny. 2002. 'The impact of torture on post-traumatic stress symptoms in war-affected Tamil refugees and immigrants', *Comprehensive psychiatry*, 43: 49-55.

Silva, Padmal De. 1993. 'Post-traumatic stress disorder: Cross-cultural aspects', *International Review of Psychiatry*, 5: 217-29.

Silver, Steven M. 2014. 'An inpatient program for post-traumatic stress disorder: Context as treatment.' in, *Trauma and its wake* (Routledge).

Silverman, Joel J. 1986. 'Post-traumatic stress disorder', *Advances in psychosomatic medicine*, 16: 115-40.

Simani, Leila, Mahtab Ramezani, Ilad Alavi Darazam, Mastooreh Sagharichi, Mohammad Amin Aalipour, Fatemeh Ghorbani, and Hossein Pakdaman. 2021. 'Prevalence and correlates of chronic fatigue syndrome and post-traumatic stress disorder after the outbreak of the COVID-19', *Journal of neurovirology*, 27: 154-59.

Skogstad, M, M Skorstad, A Lie, H Sl Conradi, T Heir, and L Weisæth. 2013. 'Work-related post-traumatic stress disorder', *Occupational medicine*, 63: 175-82.

Sloan, Patrick. 1988. 'Post-traumatic stress in survivors of an airplane crash-landing: A clinical and exploratory research intervention', *Journal of Traumatic stress*, 1: 211-29.

Smith, Meredith Y, William H Redd, Caroline Peyser, and Dan Vogl. 1999. 'Post‐traumatic stress disorder in cancer: a review', *Psycho‐Oncology: Journal of the Psychological, Social and Behavioral Dimensions of Cancer*, 8: 521-37.

Smith, Patrick, Sean Perrin, William Yule, and David M Clark. 2014. *Post traumatic stress disorder: Cognitive therapy with children and young people* (Routledge).

Smith, Sophia K, Sheryl Zimmerman, Christianna S Williams, John S Preisser, and Elizabeth C Clipp. 2008. 'Post-traumatic stress outcomes in non-Hodgkin's lymphoma survivors', *Journal of Clinical Oncology*, 26: 934-41.

Smyth, Joshua M, Jill R Hockemeyer, and Heather Tulloch. 2008. 'Expressive writing and post‐traumatic stress disorder: Effects on trauma symptoms, mood states, and cortisol reactivity', *British Journal of Health Psychology*, 13: 85-93.

Söderquist, Johan, Barbro Wijma, and Klaas Wijma. 2006. 'The longitudinal course of post-traumatic stress after childbirth', *Journal of Psychosomatic Obstetrics & Gynecology*, 27: 113-19.

Sojka, Peter, Britt-Marie Stålnacke, Ulf Björnstig, and Kurt Karlsson. 2006. 'One-year follow-up of patients with mild traumatic brain injury: occurrence of post-traumatic stress-related symptoms at follow-up and serum levels of cortisol, S-100B and neuron-specific enolase in acute phase', *Brain Injury*, 20: 613-20.

Solomon, Zahava, R Dekel, and M Mikulincer. 2008. 'Complex trauma of war captivity: A prospective study of attachment and post-traumatic stress disorder', *Psychological medicine*, 38: 1427-34.

Solomon, Zahava, Mario Mikulincer, and Rami Benbenishty. 1989. 'Locus of control and combat‐related post‐traumatic stress disorder: The intervening role of battle intensity, threat appraisal and coping', *British Journal of Clinical Psychology*, 28: 131-44.

Sonne, Susan C, Sudie E Back, Claudia Diaz Zuniga, Carrie L Randall, and Kathleen T Brady. 2003. 'Gender differences in individuals with comorbid alcohol dependence and post-traumatic stress disorder', *American Journal on Addictions*, 12: 412-23.

Speckens, Anne EM, Anke Ehlers, Ann Hackmann, Florian A Ruths, and David M Clark. 2007. 'Intrusive memories and rumination in patients with post-traumatic stress disorder: A phenomenological comparison', *Memory*, 15: 249-57.

Spence Laschinger, Heather K, and Amanda Nosko. 2015. 'Exposure to workplace bullying and post‐traumatic stress disorder symptomology: the role of protective psychological resources', *Journal of nursing management*, 23: 252-62.

Spinhoven, Philip, Brenda W Penninx, Andriana Krempeniou, Albert M van Hemert, and Bernet Elzinga. 2015. 'Trait rumination predicts onset of Post-Traumatic Stress Disorder through trauma-related cognitive appraisals: A 4-year longitudinal study', *Behaviour research and therapy*, 71: 101-09.

Spitzer, Carsten, Manuela Dudeck, Heike Liss, Stefan Orlob, Michael Gillner, and Harald J Freyberger. 2001. 'Post-traumatic stress disorder in forensic inpatients', *Journal of Forensic Psychiatry*, 12: 63-77.

Spoormaker, Victor I, and Paul Montgomery. 2008. 'Disturbed sleep in post-traumatic stress disorder: secondary symptom or core feature?', *Sleep medicine reviews*, 12: 169-84.

Stallard, Paul, and Elisabeth Smith. 2007. 'Appraisals and cognitive coping styles associated with chronic post‐traumatic symptoms in child road traffic accident survivors', *Journal of child psychology and psychiatry*, 48: 194-201.

Stallard, Paul, R Velleman, J Langsford, and S Baldwin. 2001. 'Coping and psychological distress in children involved in road traffic accidents', *British Journal of Clinical Psychology*, 40: 197-208.

Stallard, Paul, Richard Velleman, and Sarah Baldwin. 1998. 'Prospective study of post-traumatic stress disorder in children involved in road traffic accidents', *Bmj*, 317: 1619-23.

Stankovic, Lisa. 2011. 'Transforming trauma: a qualitative feasibility study of integrative restoration (iRest) yoga Nidra on combat-related post-traumatic stress disorder', *International journal of yoga therapy*, 21: 23-37.

Stark, Eloise A, CE Parsons, TJ Van Hartevelt, M Charquero-Ballester, Hugh McManners, A Ehlers, A Stein, and ML Kringelbach. 2015. 'Post-traumatic stress influences the brain even in the absence of symptoms: a systematic, quantitative meta-analysis of neuroimaging studies', *Neuroscience & Biobehavioral Reviews*, 56: 207-21.

Stein, Dan J, Soraya Seedat, Amy Iversen, and Simon Wessely. 2007. 'Post-traumatic stress disorder: medicine and politics', *The Lancet*, 369: 139-44.

Stein, Murray B, and Colleen Kennedy. 2001. 'Major depressive and post-traumatic stress disorder comorbidity in female victims of intimate partner violence', *Journal of affective disorders*, 66: 133-38.

Steinglass, Peter, and Ellen Gerrity. 1990. 'Natural Disasters and Post‐traumatic Stress Disorder Short‐Term versus Long‐Term Recovery in Two Disaster‐Affected Communities 1', *Journal of Applied Social Psychology*, 20: 1746-65.

Steketee, Gail, and Edna B Foa. 1987. 'Rape victims: Post-traumatic stress responses and their treatment:: A review of the literature', *Journal of anxiety disorders*, 1: 69-86.

Stevens, Jennifer S, and Tanja Jovanovic. 2019. 'Role of social cognition in post‐traumatic stress disorder: A review and meta‐analysis', *Genes, Brain and Behavior*, 18: e12518.

Stewart, Sherry H, Terry L Mitchell, Kristi D Wright, and Pamela Loba. 2004. 'The relations of PTSD symptoms to alcohol use and coping drinking in volunteers who responded to the Swissair Flight 111 airline disaster', *Journal of anxiety disorders*, 18: 51-68.

Stoll, Christian, Gustav Schelling, Alwin E Goetz, Erich Kilger, Andreas Bayer, Hans-Peter Kapfhammer, Hans-Bernd Rothenhäusler, Eckart Kreuzer, Bruno Reichart, and Klaus Peter. 2000. 'Health-related quality of life and post-traumatic stress disorder in patients after cardiac surgery and intensive care treatment', *The Journal of thoracic and cardiovascular surgery*, 120: 505-12.

Suliman, Sharain, Zyrhea Troeman, Dan J Stein, and Soraya Seedat. 2013. 'Predictors of acute stress disorder severity', *Journal of affective disorders*, 149: 277-81.

Sullivan, Michael JL, Pascal Thibault, Maureen J Simmonds, Maria Milioto, André-Philippe Cantin, and Ana M Velly. 2009. 'Pain, perceived injustice and the persistence of post-traumatic stress symptoms during the course of rehabilitation for whiplash injuries', *Pain*, 145: 325-31.

Summerfield, Derek. 2001. 'The invention of post-traumatic stress disorder and the social usefulness of a psychiatric category', *Bmj*, 322: 95-98.

Sumner, Jennifer A, Laura D Kubzansky, Andrea L Roberts, Paola Gilsanz, Qixuan Chen, Ashley Winning, John P Forman, Eric B Rimm, and Karestan C Koenen. 2016. 'Post-traumatic stress disorder symptoms and risk of hypertension over 22 years in a large cohort of younger and middle-aged women', *Psychological medicine*, 46: 3105-16.

Sun, Yawen, Zhen Wang, Weina Ding, Jieqing Wan, Zhiguo Zhuang, Yong Zhang, Yijun Liu, Yan Zhou, and Jianrong Xu. 2013. 'Alterations in white matter microstructure as vulnerability factors and acquired signs of traffic accident-induced PTSD', *PLoS One*, 8: e83473.

Sutherland, Kylie, and Richard A Bryant. 2005. 'Self‐defining memories in post‐traumatic stress disorder', *British Journal of Clinical Psychology*, 44: 591-98.

Symes, Lene. 1995. 'Post traumatic stress disorder: An evolving concept', *Archives of Psychiatric Nursing*, 9: 195-202.

Taal, LA, and AW Faber. 1997. 'Post-traumatic stress, pain and anxiety in adult burn victims', *Burns*, 23: 545-49.

Tang, Bihan, Qiangyu Deng, Deborah Glik, Junqiang Dong, and Lulu Zhang. 2017. 'A meta-analysis of risk factors for post-traumatic stress disorder (PTSD) in adults and children after earthquakes', *International journal of environmental research and public health*, 14: 1537.

Tang, Chulei, Lloyd Goldsamt, Jingjing Meng, Xueling Xiao, Li Zhang, Ann Bartley Williams, and Honghong Wang. 2020. 'Global estimate of the prevalence of post-traumatic stress disorder among adults living with HIV: a systematic review and meta-analysis', *BMJ open*, 10: e032435.

Tang, Liling, Lingling Pan, Liping Yuan, and Lei Zha. 2017. 'Prevalence and related factors of post-traumatic stress disorder among medical staff members exposed to H7N9 patients', *International journal of nursing sciences*, 4: 63-67.

Tanveer, Urwa, Numan Liaquat, Taimoor Anwar, and Hira Ambreen. 2023. 'Post-Traumatic Stress Disorder and Its Correlates among Road Traffic Accident Victims', *Pak-Euro Journal of Medical and Life Sciences*, 6: 01-08.

Tareen, Amina, M Elena Garralda, and Matthew Hodes. 2007. 'Post-traumatic stress disorder in childhood', *Archives of Disease in Childhood-Education and Practice*, 92: ep1-ep6.

Tarrier, Nicholas, Claire Sommerfield, Hazel Pilgrim, and Brian Faragher. 2000. 'Factors associated with outcome of cognitive-behavioural treatment of chronic post-traumatic stress disorder', *Behaviour research and therapy*, 38: 191-202.

Tarsitani, Lorenzo, Paolo Vassalini, Alexia Koukopoulos, Cristian Borrazzo, Federica Alessi, Chiara Di Nicolantonio, Riccardo Serra, Francesco Alessandri, Giancarlo Ceccarelli, and Claudio Maria Mastroianni. 2021. 'Post-traumatic stress disorder among COVID-19 survivors at 3-month follow-up after hospital discharge', *Journal of general internal medicine*, 36: 1702-07.

Taubman-Ben-Ari, Orit, Justin Rabinowitz, David Feldman, and R Vaturi. 2001. 'Post-traumatic stress disorder in primary-care settings: prevalence and physicians' detection', *Psychological medicine*, 31: 555-60.

Tay, Alvin Kuowei, Susan Rees, Jack Chen, Moses Kareth, and Derrick Silove. 2015. 'The structure of post-traumatic stress disorder and complex post-traumatic stress disorder amongst West Papuan refugees', *BMC psychiatry*, 15: 1-17.

Tehrani, Noreen. 2004. 'Bullying: a source of chronic post traumatic stress?', *British journal of guidance & counselling*, 32: 357-66.

Telles, Shirley, Nilkamal Singh, Meesha Joshi, and Acharya Balkrishna. 2010. 'Post traumatic stress symptoms and heart rate variability in Bihar flood survivors following yoga: a randomized controlled study', *BMC psychiatry*, 10: 1-10.

Thabet, Abdel Aziz Mousa, and Panos Vostanis. 1999. 'Post‐traumatic stress reactions in children of war', *Journal of child psychology and psychiatry*, 40: 385-91.

Thabet, Abdel Aziz, and Panos Vostanis. 2000. 'Post traumatic stress disorder reactions in children of war: a longitudinal study', *Child abuse & neglect*, 24: 291-98.

Thapa, Prakash, Lumeshor Acharya, Bhup Dev Bhatta, Suman Bhatta Paneru, Jai Bahadur Khattri, Prashant Kumar Chakraborty, and Rajasee Sharma. 2018. 'Anxiety, depression and post-traumatic stress disorder after earthquake', *Journal of Nepal Health Research Council*, 16: 53-57.

Thompson, JA, PFC Charlton, R Kerry, D Lee, and SW Turner. 1995. 'An open trial of exposure therapy based on deconditioning for post‐traumatic stress disorder', *British Journal of Clinical Psychology*, 34: 407-16.

Thrasher, SM, Tim Dalgleish, and William Yule. 1994. 'Information processing in post-traumatic stress disorder', *Behaviour research and therapy*, 32: 247-54.

Tomb, David A. 1994. 'The phenomenology of post-traumatic stress disorder', *Psychiatric Clinics*, 17: 237-50.

Trickey, David, Andy P Siddaway, Richard Meiser-Stedman, Lucy Serpell, and Andy P Field. 2012. 'A meta-analysis of risk factors for post-traumatic stress disorder in children and adolescents', *Clinical psychology review*, 32: 122-38.

Truszczyńska-Baszak, Aleksandra, Monika Guszkowska, Emilia Dadura, and Adam Tarnowski. 2023. 'Prognostic factors of post-traumatic stress disorder risk in patients with surgical treatment of hip acetabular fracture. Original study', *Current Psychology*, 42: 1034-43.

Tucker, Phebe, Betty Pfefferbaum, Sara Jo Nixon, and Warren Dickson. 2000. 'Predictors of post-traumatic stress symptoms in Oklahoma City: Exposure, social support, peri-traumatic responses', *The Journal of Behavioral Health Services & Research*, 27: 406-16.

Turnbull, Gordon J. 1998. 'A review of post-traumatic stress disorder. Part I: Historical development and classification', *Injury*, 29: 87-91.

Uddo, Madeline, Jennifer J Vasterling, Kevin Brailey, and Patricia B Sutker. 1993. 'Memory and attention in combat-related post-traumatic stress disorder (PTSD)', *Journal of Psychopathology and Behavioral Assessment*, 15: 43-52.

Udomratn, Pichet. 2008. 'Mental health and the psychosocial consequences of natural disasters in Asia', *International Review of Psychiatry*, 20: 441-44.

Udwin, Orlee, Stephanie Boyle, William Yule, Derek Bolton, and Dominic O'Ryan. 2000. 'Risk factors for long-term psychological effects of a disaster experienced in adolescence: Predictors of post traumatic stress disorder', *The Journal of Child Psychology and Psychiatry and Allied Disciplines*, 41: 969-79.

Ullman, Sarah E, Cynthia J Najdowski, and Henrietta H Filipas. 2009. 'Child sexual abuse, post-traumatic stress disorder, and substance use: Predictors of revictimization in adult sexual assault survivors', *Journal of child sexual abuse*, 18: 367-85.

Ursano, Robert J, Carol S Fullerton, Richard S Epstein, Brian Crowley, Tzu-Cheg Kao, Kelley Vance, Karrie J Craig, Angela L Dougall, and Andrew Baum. 1999. 'Acute and chronic posttraumatic stress disorder in motor vehicle accident victims', *American Journal of Psychiatry*, 156: 589-95.

Utzon-Frank, Nicolai, Nina Breinegaard, Mette Bertelsen, Marianne Borritz, Nanna Hurwitz Eller, Merete Nordentott, Kasper Olesen, Naja Hulvej Rod, Reiner Rugulies, and Jens Peter Bonde. 2014. 'Occurrence of delayed-onset post-traumatic stress disorder: a systematic review and meta-analysis of prospective studies', *Scandinavian Journal of Work, Environment & Health*: 215-29.

Vaiva, Guillaume, Pierre Thomas, François Ducrocq, Monique Fontaine, Virginie Boss, Patrick Devos, Claire Rascle, Olivier Cottencin, Alain Brunet, and Philippe Laffargue. 2004. 'Low posttrauma GABA plasma levels as a predictive factor in the development of acute posttraumatic stress disorder', *Biological psychiatry*, 55: 250-54.

van den Heuvel, Leigh, Sharain Suliman, Stefanie Malan-Müller, Sian Hemmings, and Soraya Seedat. 2016. 'Brain-derived neurotrophic factor Val66met polymorphism and plasma levels in road traffic accident survivors', *Anxiety, Stress, & Coping*, 29: 616-29.

van Der Kolk, Bessel, Julian D Ford, and Joseph Spinazzola. 2019. 'Comorbidity of developmental trauma disorder (DTD) and post-traumatic stress disorder: Findings from the DTD field trial', *European Journal of Psychotraumatology*, 10: 1562841.

Van Emmerik, Arnold AP, Jan H Kamphuis, Alexander M Hulsbosch, and Paul MG Emmelkamp. 2002. 'Single session debriefing after psychological trauma: A meta-analysis', *The Lancet*, 360: 766-71.

Van Praag, Dominique LG, Maryse C Cnossen, Suzanne Polinder, Lindsay Wilson, and Andrew IR Maas. 2019. 'Post-traumatic stress disorder after civilian traumatic brain injury: a systematic review and meta-analysis of prevalence rates', *Journal of neurotrauma*, 36: 3220-32.

Varma, Kajal, Gayathri Paturi, Juluri Sumana Sri, Gopireddy Shiva Reddy, and P Rajashekar. 2024. 'POST-TRAUMATIC STRESS DISORDER–AN OVERVIEW'.

Vera, Maria Paz García, and Jesús Sanz. 2017. 'Psychopathological consequences of terrorism: the prevalence of post-traumatic stress disorder in victims of terrorist attacks.' in, *Human Rights Issues and Vulnerable Groups* (Bentham Science Publishers).

Verger, Pierre, William Dab, Donna L Lamping, Jean-Yves Loze, Céline Deschaseaux-Voinet, Lucien Abenhaim, and Frédéric Rouillon. 2004. 'The psychological impact of terrorism: an epidemiologic study of posttraumatic stress disorder and associated factors in victims of the 1995–1996 bombings in France', *American Journal of Psychiatry*, 161: 1384-89.

Videlock, Elizabeth J, Tamar Peleg, Ronen Segman, Rachel Yehuda, Roger K Pitman, and Arieh Y Shalev. 2008. 'Stress hormones and post-traumatic stress disorder in civilian trauma victims: a longitudinal study. Part II: the adrenergic response', *International Journal of Neuropsychopharmacology*, 11: 373-80.

Villain, Hélène, Aïcha Benkahoul, Philippe Birmes, Barbara Ferry, and Pascal Roullet. 2018. 'Influence of early stress on memory reconsolidation: implications for post-traumatic stress disorder treatment', *PLoS One*, 13: e0191563.

Villamor, Ana, and Estibaliz Sáez de Adana. 2014. 'Gender differences in post-traumatic stress disorder.' in, *Psychopathology in women: Incorporating gender perspective into descriptive psychopathology* (Springer).

Vincent, Heather K, MaryBeth Horodyski, Kevin R Vincent, Sonya T Brisbane, and Kalia K Sadasivan. 2015. 'Psychological distress after orthopedic trauma: prevalence in patients and implications for rehabilitation', *PM&R*, 7: 978-89.

Vincent, Jean-Louis, J Griffiths, AM Hull, and BH Cuthbertson. 2008. "Post-traumatic stress disorder in intensive care unit survivors." In *Yearbook of Intensive Care and Emergency Medicine*, 891-905. Springer.

Vukojevic, Vanja, Iris-T Kolassa, Matthias Fastenrath, Leo Gschwind, Klara Spalek, Annette Milnik, Angela Heck, Christian Vogler, Sarah Wilker, and Philippe Demougin. 2014. 'Epigenetic modification of the glucocorticoid receptor gene is linked to traumatic memory and post-traumatic stress disorder risk in genocide survivors', *Journal of Neuroscience*, 34: 10274-84.

Wadsworth, Martha E, Catherine DeCarlo Santiago, and Lindsey Einhorn. 2009. 'Coping with displacement from Hurricane Katrina: predictors of one-year post-traumatic stress and depression symptom trajectories', *Anxiety, Stress, & Coping*, 22: 413-32.

Wahab, Suzaily, Li Ling Yong, Wei Keong Chieng, Myristica Yamil, Noor Azah Sawal, Nurul Qiyaam Abdullah, Cyntiya Rahmawati Muhdisin Noor, Siti Mardiyah Wd Wiredarma, Rosnah Ismail, and Aisya Hanim Othman. 2021. 'Post-traumatic stress symptoms in adolescents exposed to the earthquake in Lombok, Indonesia: Prevalence and association with maladaptive trauma-related cognition and resilience', *Frontiers in psychiatry*, 12: 680393.

Walker, Lenore E. 1991. 'Post-traumatic stress disorder in women: Diagnosis and treatment of battered woman syndrome', *Psychotherapy: Theory, Research, Practice, Training*, 28: 21.

Walshe, David G, Elizabeth J Lewis, Sun I Kim, Kathleen O'Sullivan, and Brenda K Wiederhold. 2003. 'Exploring the use of computer games and virtual reality in exposure therapy for fear of driving following a motor vehicle accident', *Cyberpsychology & behavior*, 6: 329-34.

Walters, James TR, Jonathan I Bisson, and Jonathan P Shepherd. 2007. 'Predicting post-traumatic stress disorder: validation of the Trauma Screening Questionnaire in victims of assault', *Psychological medicine*, 37: 143-50.

Wang, Ching‐Hui, Shiow‐Luan Tsay, and A Elaine Bond. 2005. 'Post‐traumatic stress disorder, depression, anxiety and quality of life in patients with traffic‐related injuries', *Journal of advanced nursing*, 52: 22-30.

Wang, Li, Zhanbiao Shi, Yuqing Zhang, and Zhen Zhang. 2010. 'Psychometric properties of the 10‐item Connor–Davidson Resilience Scale in Chinese earthquake victims', *Psychiatry and clinical neurosciences*, 64: 499-504.

Wang, Li, Yuqing Zhang, Wenzhong Wang, Zhanbiao Shi, Jianhua Shen, Ming Li, and Yong Xin. 2009. 'Symptoms of posttraumatic stress disorder among adult survivors three months after the Sichuan earthquake in China', *Journal of Traumatic stress*, 22: 444-50.

Wang, Xin, Hong Xie, Andrew S Cotton, Kristopher R Brickman, Terrence J Lewis, John T Wall, Marijo B Tamburrino, William R Bauer, Kenny Law, and Samuel A McLean. 2017. 'Early changes in cortical emotion processing circuits after mild traumatic brain injury from motor vehicle collision', *Journal of neurotrauma*, 34: 273-80.

Wang, Xue, Xiahong Li, Miao Qi, Xiuli Hu, Huiping Zhu, and Xiuquan Shi. 2022. 'Incidence of post-traumatic stress disorder in survivors of traumatic fracture: A systematic review and meta-analysis', *Psychology, Health & Medicine*, 27: 902-16.

Watson, Paddy Burges. 1987. 'Post‐traumatic stress disorder in Australia and New Zealand: a clinical review of the consequences of inescapable horror', *Medical journal of Australia*, 147: 443-47.

Weems, Carl F, Leslie K Taylor, Melinda F Cannon, Reshelle C Marino, Dawn M Romano, Brandon G Scott, Andre M Perry, and Vera Triplett. 2010. 'Post traumatic stress, context, and the lingering effects of the Hurricane Katrina disaster among ethnic minority youth', *Journal of abnormal child psychology*, 38: 49-56.

Wei, Chuguang, Jin Han, Yuqing Zhang, Walter Hannak, and Zhengkui Liu. 2017. 'The characteristics of emotional response of post-traumatic stress disorder and post-traumatic growth among Chinese adults exposed to an explosion incident', *Frontiers in public health*, 5: 3.

Weisæth, Lars. 1989a. 'Importance of high response rates in traumatic stress research', *Acta Psychiatrica Scandinavica*, 80: 131-37.

Wentworth, Bailey A, Murray B Stein, Laura S Redwine, Yang Xue, Pam R Taub, Paul Clopton, Keshav R Nayak, and Alan S Maisel. 2013. 'Post-traumatic stress disorder: a fast track to premature cardiovascular disease?', *Cardiology in review*, 21: 16-22.

Whiting, Diane, and Richard A Bryant. 2007. 'Role of appraisals in expressed anger after trauma', *Clinical Psychologist*, 11: 33-36.

Wiederhold, Brenda K, and Mark D Wiederhold. 2010. 'Virtual reality treatment of posttraumatic stress disorder due to motor vehicle accident', *Cyberpsychology, behavior, and social networking*, 13: 21-27.

Wilcoxon, Lucy A, Richard Meiser-Stedman, and Aaron Burgess. 2021. 'Post-traumatic stress disorder in parents following their child’s single-event trauma: A meta-analysis of prevalence rates and risk factor correlates', *Clinical child and family psychology review*, 24: 725-43.

Wild, J, KV Smith, E Thompson, F Béar, MJJ Lommen, and A Ehlers. 2016. 'A prospective study of pre-trauma risk factors for post-traumatic stress disorder and depression', *Psychological medicine*, 46: 2571-82.

Williams, Monnica T, S Cahill, and E Foa. 2010. 'Psychotherapy for post-traumatic stress disorder', *Textbook of anxiety disorders*: 603-27.

Williams, Shehan S, Chamara A Wijesinghe, Shaluka F Jayamanne, Nicholas A Buckley, Andrew H Dawson, David G Lalloo, and H Janaka de Silva. 2011. 'Delayed psychological morbidity associated with snakebite envenoming', *PLoS neglected tropical diseases*, 5: e1255.

Wilson, John P. 1994. 'The historical evolution of PTSD diagnostic criteria: From Freud to DSM-IV', *Journal of Traumatic stress*, 7: 681-98.

Wilson, John P, W Ken Smith, and Suzanne K Johnson. 2013. 'A comparative analysis of PTSD among various survivor groups.' in, *Trauma and its wake* (Routledge).

Wilson, John P, and Sheldon D Zigelbaum. 1983. 'The Vietnam veteran on wal: The relation of post‐traumatic stress disorder to criminal behavior', *Behavioral Sciences & the Law*, 1: 69-83.

Wilson, Priscilla, Gladys Dzansi, and Lilian Akorfa Ohene. 2020. '‘I don’t want to think about it’: Psychosocial experiences of road traffic accident survivors in Ghana', *International Emergency Nursing*, 53: 100935.

Wise, Anna E, and Douglas L Delahanty. 2017. 'Parental factors associated with child post-traumatic stress following injury: A consideration of intervention targets', *Frontiers in psychology*, 8: 1412.

Wiseman, Taneal, Kim Foster, and Kate Curtis. 2013. 'Mental health following traumatic physical injury: an integrative literature review', *Injury*, 44: 1383-90.

Wu, Hui‐Ching. 2011. 'The protective effects of resilience and hope on quality of life of the families coping with the criminal traumatisation of one of its members', *Journal of Clinical Nursing*, 20: 1906-15.

Wu, Zhibin, Jiuping Xu, and Lili He. 2014. 'Psychological consequences and associated risk factors among adult survivors of the 2008 Wenchuan earthquake', *BMC psychiatry*, 14: 1-11.

Xiong, Xu, Emily W Harville, Donald R Mattison, Karen Elkind-Hirsch, Gabriella Pridjian, and Pierre Buekens. 2010. 'Hurricane Katrina experience and the risk of post-traumatic stress disorder and depression among pregnant women', *American journal of disaster medicine*, 5: 181.

Yaalaoui, S, K Chihabeddine, E Boukind, and D Moussaoui. 2002. 'Post-traumatic stress disorder in burned patients', *Burns*, 28: 647-50.

Yang, Luodong, Haohao Li, Yao Meng, Yan Shi, Anxin Ge, Guiqing Zhang, and Chaomeng Liu. 2022. 'Dynamic changes in brain structure in patients with post-traumatic stress disorder after motor vehicle accident: A voxel-based morphometry-based follow-up study', *Frontiers in psychology*, 13: 1018276.

Yang, Wanqiu, Ke Cui, Timothy Sim, Jun Zhang, Yanchun Yang, and Xiaohong Ma. 2020. 'Health-related quality of life and post-traumatic stress disorder in inpatients injured in the Ludian earthquake: a longitudinal study', *Health and quality of life outcomes*, 18: 1-11.

Yehuda, Rachel. 1998. 'Psychoneuroendocrinology of post-traumatic stress disorder', *Psychiatric Clinics of North America*, 21: 359-79.

Yehuda, Rachel, Julia A Golier, Lisa Tischler, Philip D Harvey, Randall Newmark, Ren Kui Yang, and Monte S Buchsbaum. 2007. 'Hippocampal volume in aging combat veterans with and without post-traumatic stress disorder: relation to risk and resilience factors', *Journal of psychiatric research*, 41: 435-45.

Yehuda, Rachel, Charles W Hoge, Alexander C McFarlane, Eric Vermetten, Ruth A Lanius, Caroline M Nievergelt, Stevan E Hobfoll, Karestan C Koenen, Thomas C Neylan, and Steven E Hyman. 2015. 'Post-traumatic stress disorder', *Nature reviews Disease primers*, 1: 1-22.

Yehuda, Rachel, AlexanderC McFarlane, and AriehY Shalev. 1998. 'Predicting the development of posttraumatic stress disorder from the acute response to a traumatic event', *Biological psychiatry*, 44: 1305-13.

Yi, Lei, Yunling Lian, Ning Ma, and Ni Duan. 2022. 'A randomized controlled trial of the influence of yoga for women with symptoms of post-traumatic stress disorder', *Journal of translational medicine*, 20: 162.

Young, Allan. 1997. *The harmony of illusions: Inventing post-traumatic stress disorder* (Princeton University Press).

Young, Bruce, and Dudley Blake. 2020. *Group Treatment for Post Traumatic Stress Disorder: Conceptualization, Themes and Processes* (Routledge).

Yrondi, Antoine, Raphael DerKasbarian, Adeline Gallini, Vincent Max, Christophe Pauron, Audrey Joubin, Jacky Laguerre, Christian Virenque, and Philippe Birmes. 2019. 'Symptoms of depression and post-traumatic stress in a group of lightning strike victims', *Journal of psychosomatic research*, 120: 90-95.

Yule, William. 2001. 'Post-traumatic stress disorder in children and adolescents', *International Review of Psychiatry*, 13: 194-200.

Yule, William, Derek Bolton, Orlee Udwin, Stephanie Boyle, Dominic O'Ryan, and Julie Nurrish. 2000. 'The long-term psychological effects of a disaster experienced in adolescence: I: The incidence and course of PTSD', *The Journal of Child Psychology and Psychiatry and Allied Disciplines*, 41: 503-11.

Yule, William, and Patrick Smith. 2015. 'Post traumatic stress disorder', *Rutter's child and adolescent psychiatry*: 806-21.

Yule, William, and Orlee Udwin. 1991. 'Screening child survivors for post‐traumatic stress disorders: Experiences from the ‘Jupiter’sinking', *British Journal of Clinical Psychology*, 30: 131-38.

Yule, William, and Ruth M Williams. 1990. 'Post‐traumatic stress reactions in children', *Journal of Traumatic stress*, 3: 279-95.

Zatzick, Douglas F, Gregory J Jurkovich, Larry Gentilello, David Wisner, and Fredrick P Rivara. 2002. 'Posttraumatic stress, problem drinking, and functional outcomes after injury', *Archives of Surgery*, 137: 200-05.

Zatzick, Douglas F, Frederick P Rivara, Avery B Nathens, Gregory J Jurkovich, JIN Wang, Ming-Yu Fan, Joan Russo, David S Salkever, and Ellen J Mackenzie. 2007. 'A nationwide US study of post-traumatic stress after hospitalization for physical injury', *Psychological medicine*, 37: 1469-80.

Zeidner, Moshe, and Hasida Ben-Zur. 1994. 'Individual differences in anxiety, coping, and post-traumatic stress in the aftermath of the Persian Gulf War', *Personality and Individual Differences*, 16: 459-76.

Zen, Angelica L, Mary A Whooley, Shoujun Zhao, and Beth E Cohen. 2012. 'Post-traumatic stress disorder is associated with poor health behaviors: findings from the heart and soul study', *Health psychology*, 31: 194.

Zhang, Weiqing, Hui Liu, Xiaolian Jiang, Dongmei Wu, and Yali Tian. 2014. 'A longitudinal study of posttraumatic stress disorder symptoms and its relationship with coping skill and locus of control in adolescents after an earthquake in China', *PLoS One*, 9: e88263.

Zhang, Yan, Fanchang Kong, Lin Wang, Hong Chen, Xiao Gao, Xiaohong Tan, Han Chen, Jianguo Lv, and Yong Liu. 2010. 'Mental health and coping styles of children and adolescent survivors one year after the 2008 Chinese earthquake', *Children and Youth Services Review*, 32: 1403-09.

Zhang, Yuqing, and Samuel MY Ho. 2011. 'Risk factors of posttraumatic stress disorder among survivors after the 512 Wenchuan earthquake in China', *PLoS One*, 6: e22371.

Zhang, Z, Z Shi, L Wang, and M Liu. 2011. 'One year later: Mental health problems among survivors in hard-hit areas of the Wenchuan earthquake', *Public health*, 125: 293-300.

Zhou, Xiao, Xinchun Wu, and Rui Zhen. 2018. 'Self-esteem and hope mediate the relations between social support and post-traumatic stress disorder and growth in adolescents following the Ya’an earthquake', *Anxiety, Stress, & Coping*, 31: 32-45.

Zlotnick, Caron, Jennifer Johnson, Robert Kohn, Benjamin Vicente, Pedro Rioseco, and Sandra Saldivia. 2006. 'Epidemiology of trauma, post-traumatic stress disorder (PTSD) and co-morbid disorders in Chile', *Psychological medicine*, 36: 1523-33.

Zohar, Joseph, Alzbeta Juven-Wetzler, Rachel Sonnino, Shlomit Cwikel-Hamzany, Evgenya Balaban, and Hagit Cohen. 2011. 'New insights into secondary prevention in post-traumatic stress disorder', *Dialogues in clinical neuroscience*, 13: 301-09.

Zoladz, Phillip R, Cheryl D Conrad, Monika Fleshner, and David M Diamond. 2008. 'Acute episodes of predator exposure in conjunction with chronic social instability as an animal model of post-traumatic stress disorder', *Stress*, 11: 259-81.

Zoladz, Phillip R, and David M Diamond. 2013. 'Current status on behavioral and biological markers of PTSD: a search for clarity in a conflicting literature', *Neuroscience & Biobehavioral Reviews*, 37: 860-95.
