# Supplementary material for: Post-traumatic stress disorder and associated factors among road traffic accident survivors in Sub-Saharan Africa: A systematic review and meta-analysis
Source: PLoS One. 2025 Feb 24;20(2):e0318714. doi: 10.1371/journal.pone.0318714 (PMC11849852; doi:10.1371/journal.pone.0318714)
Supplement: S2 Table — (DOCX) [file pone.0318714.s003.docx]

**Supplemental Table 2:** Quality assessment of the included studies using the Joanna Briggs Institute (JBI) quality appraisal criteria

| 1. **For cross-sectional studies** | | | | | | | | | | | | | | | | | | | |  |
| --- | --- | --- | --- | --- | --- | --- | --- | --- | --- | --- | --- | --- | --- | --- | --- | --- | --- | --- | --- | --- |
| **S/N** | | | | **Author [Year]** | **Criteria** | | | | | | | | | | | **Scores** | | | **Overall quality** |  |
|  | | | |  | Clearly defined inclusion criteria | Describing the study settings participants | Valid &reliable exposure measurement | Objective &standard criteria for measurement | Identified confounder | Strategies to deal with confounder | | Valid & reliable outcome measurement | Appropriate statistical analysis | |  | | |  | |  |
|  | | | | Ajibade BL [2015] | Y | Y | Y | Y | N | N | | Y | Y | | 6 | | | Low risk | |  |
|  | | | | Alenko A [2019] | Y | Y | Y | Y | N | Y | | Y | Y | | 7 | | | Low risk | |  |
|  | | | | Atwoli L [2013] | Y | Y | Y | Y | N | N | | Y | Y | | 6 | | | Low risk | |  |
|  | | | | Bedaso A [2020] | Y | Y | Y | Y | N | Y | | Y | Y | | 7 | | | Low risk | |  |
|  | | | | Daddah D [2022] | Y | Y | Y | Y | N | Y | | Y | Y | | 7 | | | Low risk | |  |
|  | | | | Fekadu W [2019] | Y | Y | Y | Y | N | Y | | Y | Y | | 7 | | | Low risk | |  |
|  | | | | Golja EA [2020] | Y | Y | Y | Y | N | Y | | Y | Y | | 7 | | | Low risk | |  |
|  | | | | Isabirye RA [2022] | Y | Y | Y | Y | N | Y | | Y | Y | | 7 | | | Low risk | |  |
|  | | | | Mosaku K [2014] | Y | Y | Y | Y | N | Y | | Y | Y | | 7 | | | Low risk | |  |
|  | | | | Ongecha-Owuor F | Y | Y | Y | Y | N | N | | Y | N | | 5 | | | Low risk | |  |
|  | | | | Stein DJ [2016] | Y | Y | Y | Y | N | Y | | Y | Y | | 7 | | | Low risk | |  |
|  | | | | Suliman S [2014] | Y | Y | Y | Y | N | Y | | N | Y | | 7 | | | Low risk | |  |
|  | | | | Tamirr TT [2022] | Y | Y | Y | Y | N | Y | | Y | Y | | 7 | | | Low risk | |  |
|  | | | | Yohannes K, 2018 | Y | Y | Y | Y | N | Y | | Y | Y | | 7 | | | Low risk | |  |
|  | | | 1. **For case-control studies** | | | | | | | | | | | | | | | | | |
|  |  |  | **S/N** | **Criteria** | | | | | | | | Asukuo JE | | | Iteke O | | | Yimer GM | | |
|  |  |  | 1. 1. | Two groups are similar and recruited from the same population | | | | | | | | Y | | |  | | | Y | | |
| 1. 2. | | Similar measurement of exposure both for exposed and unexposed groups | | | | | | | | Y | | | Y | | | Y | | |  |  |
| 1. 3. | | Valid and reliable measurement of exposure | | | | | | | | Y | | | Y | | | Y | | |  |  |
| - 1. 4 | | Identifying confounders | | | | | | | | N | | | N | | | N | | |  |  |
| 1. 5. | | Strategies to deal with confounders | | | | | | | | N | | | N | | | N | | |  |  |
| 1. 6. | | Groups are free of the outcomes at the beginning | | | | | | | | Y | | | Y | | | Y | | |  |  |
| 1. 7. | | Valid and reliable measurement of outcomes | | | | | | | | Y | | | Y | | | Y | | |  |  |
| 1. 8. | | Long enough follow-up time for the occurrence of outcomes | | | | | | | | Y | | | Y | | | Y | | |  |  |
| 1. 9. | | Complete follow-up time | | | | | | | | Y | | | Y | | | Y | | |  |  |
| 1. 10. | | Strategies to address lost follow-up | | | | | | | | N | | | Y | | | Y | | |  |  |
| *Percentage (%) of ʺYesʺ* | | | | | | | | | | *7/10=80%* | | | *8/10=80%* | | | *8/10=70%* | | |  |  |

*Note: Y, yes; N, No*
