# Supplementary material for: Post-traumatic stress disorder and associated factors among road traffic accident survivors in Sub-Saharan Africa: A systematic review and meta-analysis
Source: PLoS One. 2025 Feb 24;20(2):e0318714. doi: 10.1371/journal.pone.0318714 (PMC11849852; doi:10.1371/journal.pone.0318714)
Supplement: S3 Table — (DOCX) [file pone.0318714.s004.docx]

**Supplemental Table 3:** Risk of bias assessment of the included studies

| **S/N** | **Author [Year]** | **Criteria** | | | | | | | | | | **Scores** | **Overall risk of bias** |
| --- | --- | --- | --- | --- | --- | --- | --- | --- | --- | --- | --- | --- | --- |
|  |  | **External validity** | | | | **Internal validity** | | | | | | |  |
|  |  | **Q1** | **Q2** | **Q3** | **Q4** | **Q5** | **Q6** | **Q7** | **Q8** | **Q9** | **Q10** | |  |
|  | Ajibade BL [2015] | N | Y | Y | Y | Y | N | N | Y | Y | Y | 7 | Moderate risk |
|  | Alenko A [2019] | Y | Y | Y | Y | Y | Y | N | Y | N | Y | 8 | Low risk |
|  | Asukuo JE [2017] | Y | Y | Y | Y | Y | N | Y | Y | N | Y | 8 | Low risk |
|  | Atwoli L [2013] | N | Y | Y | Y | Y | N | N | Y | Y | Y | 7 | Moderate risk |
|  | Bedaso A [2020] | Y | Y | Y | Y | Y | N | N | Y | N | Y | 8 | Low risk |
|  | Daddah D [2022] | N | Y | Y | Y | N | Y | Y | Y | Y | Y | 8 | Low risk |
|  | Fekadu W [2019] | Y | Y | Y | Y | Y | Y | N | Y | N | Y | 8 | Low risk |
|  | Golja EA [2020] | Y | Y | Y | Y | N | N | Y | Y | Y | Y | 8 | Low risk |
|  | Isabirye RA [2022] | Y | Y | Y | Y | Y | N | N | Y | Y | Y | 8 | Low risk |
|  | Iteke O [2011] | N | Y | Y | Y | Y | Y | Y | Y | Y | N | 8 | Low risk |
|  | Mosaku K [2014] | N | Y | Y | Y | Y | Y | Y | Y | Y | N | 8 | Low risk |
|  | Ongecha-Owuor FA | N | Y | Y | N | Y | N | N | Y | Y | Y | 6 | Moderate risk |
|  | Stein DJ [2016] | Y | Y | Y | Y | Y | N | Y | Y | N | Y | 8 | Low risk |
|  | Suliman S [2014] | Y | Y | Y | Y | Y | Y | N | Y | N | Y | 8 | Low risk |
|  | Tamirr TT [2022] | Y | Y | Y | Y | Y | N | N | Y | Y | Y | 8 | Low risk |
|  | Yimer GM [2023] | Y | Y | Y | Y | Y | Y | N | N | Y | Y | 8 | Low risk |
|  | Yohannes K [2018] | Y | Y | Y | Y | Y | N | Y | Y | N | Y | 8 | Low risk |

Note: Y, Yes; N, No; Q1, Representatives of the target population; Q2, Representativeness of the sampling frame; Q3, Random sampling or census; Q4, Minimal response bias; Q5, Data were collected directly; Q6, Acceptable case definition used in the study; Q7, Valid and reliable measurement; Q8, The same mode of data collection for all study subject; Q9, Appropriate length of prevalence period for parameter of interest and Q10, Appropriate numerators and denominators of interest.
