# Supplementary material for: Post-traumatic stress disorder and associated factors among road traffic accident survivors in Sub-Saharan Africa: A systematic review and meta-analysis
Source: PLoS One. 2025 Feb 24;20(2):e0318714. doi: 10.1371/journal.pone.0318714 (PMC11849852; doi:10.1371/journal.pone.0318714)
Supplement: Attachment — Submitted filename: Response_to_Reviewers_auresp_2.docx [file pone.0318714.s007.docx]

**Dear Editors and reviewers:**

We sincerely appreciate the valuable comments and suggestions you raised. The thorough review helped immensely in the shaping of the manuscript. The comments and suggestions have been closely followed and revisions have been made accordingly. The following are the questions that have been extracted from the Editor and Reviewers’ comments along with our summarized responses. Thank you very much for your constructive comments. We tried to inculcate your comments and questions as described below. The changes will be attached with

Title: Post-traumatic stress disorder and associated factors among road traffic accident survivors in Sub-Saharan Africa: A systematic review and meta-analysis

**Authors:**

*TMA:* [*tigabumunye21@gmail.com*](mailto:tigabumunye21@gmail.com)

GL: [getasewlegas@gmail.com](mailto:getasewlegas@gmail.com)

SDK: [solomondemis@gmail.com](mailto:solomondemis@gmail.com)

AK: [amarekassaw2009@gmail.com](mailto:amarekassaw2009@gmail.com)

BD: [brookmelse2022@gmail.com](mailto:brookmelse2022@gmail.com)

ABN: [adanebirhanu23@gmail.com](mailto:adanebirhanu23@gmail.com)

YA: [yirgalemabere12@gmail.com](mailto:yirgalemabere12@gmail.com)

DK: [demewozk@yahoo.com](mailto:demewozk@yahoo.com)

BMM: [biremengist21@gmail.com](mailto:biremengist21@gmail.com)

**Editor Comment #01:** Please review your reference list to ensure that it is complete and correct. If you have cited papers that have been retracted, please include the rationale for doing so in the manuscript text, or remove these references and replace them with relevant current references. Any changes to the reference list should be mentioned in the rebuttal letter that accompanies your revised manuscript. If you need to cite a retracted article, indicate the article’s retracted status in the References list and also include a citation and full reference for the retraction notice.

**Authors’ response:** Recognizing your comment, we have explored and ensured that the reference lists are complete, and we have not cited a retracted articled in the study.

**Editor Comment #02:** Line 45: Please replace depression with depression symptoms. Please consider this throughout the manuscript.

**Authors’ response:** Accepting your constructive comment, we have replaced depression with depression symptoms throughout the manuscript.

**Editor Comment #03:** Line 60: Please spell out RTA at first use in the main body of the manuscript

**Authors’ response:** Thank you for your constructive feedback! We have spelled out RTA at first use in the main body of the manuscript.

**Editor Comment #04:** Line 62: Please spell out LMICs and WHO at first use in the main body of the manuscript.

**Authors’ response:** Recognizing your constructive feedback, we have spelled out LMICs and WHO at first use in the main body of the manuscript.

**Editor Comment #05:** Line 70: Please spell out PTSD at first use in the main body of the manuscript.

**Authors’ response:** Thank you for your valuable feedback! We have spelled out PTSD at first use in the main body of the manuscript too.

**Reviewer #2:**

**Reviewer #2 comment and suggestion #01:** In line 297, the following was revised: “Furthermore, even though the AOR estimated by the case numbers could be suffered from the inflation caused by the multicollinearity of repeated counting of cases in the included studies, the results of this study revealed that female survivors had a 2.33 times higher risk of developing PTSD, according to the study’s findings…” The author indeed attempted to suggest the point that “case numbers could be suffered from the inflation…” but with “even though”. This, in English, seems to tell the reader that the next result is not a problem. I am not sure whether this is what the author intends to express because, in fact, there is a problem with the results. That is, the 2.33 times odds are inflated and so inaccurate (not incorrect but inaccurate). The author needs to be very careful about making such a statement. All the odds that the author estimated here are not absolute values but only relative values. Instead, the author should consider stating that, for example, the current data suggest that female survivors had a 2.33 times higher risk of developing PTSD, whereas the estimation of the AORs would be inflated by…and thus, the results need to be interpreted with caution.

**Authors’ response:** Recognizing your constructive feedback, we have revised this session accordingly.

**Reviewer #2 comment and suggestion #02:** The author needs to address the overestimation of AORs in the limitation. It is scientifically sound to say these factors are associated, but these estimated AORs are not. This is important. (for example, someone could quote this paper like: the author said women are twice as likely to have PTSD. But that is not accurate. It is because women are more likely to get depression. Depression holds up most of the variance of PTSD. So maybe being female is related to PTSD, but that 2.33 times odds is not the precise number to suggest. And so do all other estimated AORs.

**Authors’ response:** Thank you for your valuable feedback again! We have also addressed it in the limitation section of the manuscript.
