# Supplementary material for: Post-traumatic stress disorder and associated factors among road traffic accident survivors in Sub-Saharan Africa: A systematic review and meta-analysis
Source: PLoS One. 2025 Feb 24;20(2):e0318714. doi: 10.1371/journal.pone.0318714 (PMC11849852; doi:10.1371/journal.pone.0318714)
Supplement: Attachment — Submitted filename: Response to Reviewers.docx [file pone.0318714.s006.docx]

**Dear Editors and reviewers:**

We sincerely appreciate the valuable comments and suggestions you raised. The thorough review helped immensely in the shaping of the manuscript. The comments and suggestions have been closely followed and revisions have been made accordingly. The following are the questions that have been extracted from the Editor and Reviewers’ comments along with our summarized responses. Thank you very much for your constructive comments. We tried to inculcate your comments and questions as described below. The changes will be attached with

Title: Post-traumatic stress disorder and associated factors among road traffic accident survivors in Sub-Saharan Africa: A systematic review and meta-analysis

**Authors:**

*TMA:* [*tigabumunye21@gmail.com*](mailto:tigabumunye21@gmail.com)

GL: [getasewlegas@gmail.com](mailto:getasewlegas@gmail.com)

SDK: [solomondemis@gmail.com](mailto:solomondemis@gmail.com)

AK: [amarekassaw2009@gmail.com](mailto:amarekassaw2009@gmail.com)

BD: [brookmelse2022@gmail.com](mailto:brookmelse2022@gmail.com)

ABN: [adanebirhanu23@gmail.com](mailto:adanebirhanu23@gmail.com)

YA: [yirgalemabere12@gmail.com](mailto:yirgalemabere12@gmail.com)

DK: [demewozk@yahoo.com](mailto:demewozk@yahoo.com)

BMM: [biremengist21@gmail.com](mailto:biremengist21@gmail.com)

**Editor Comment #01:** Please ensure that your manuscript meets PLOS ONE's style requirements, including those for file naming. The PLOS ONE style templates can be found at

<https://journals.plos.org/plosone/s/file?id=wjVg/PLOSOne_formatting_sample_main_body.pdf> and 
<https://journals.plos.org/plosone/s/file?id=ba62/PLOSOne_formatting_sample_title_authors_affiliations.pdf>

**Authors’ response:** Recognizing your comment, we have looked at the PLOS ONE style templates using the given link and we have ensured that our manuscript meets PLOS ONE's style requirements, including those for file naming. The requested corrections have been included throughout the revised version of the manuscript.

**Editor Comment #02:** We noticed you have some minor occurrence of overlapping text with the following previous publication(s), which needs to be addressed:

<https://bmcpublichealth.biomedcentral.com/counter/pdf/10.1186/s12889-024-19684-3.pdf>?
<https://karger.com/dem/article/doi/10.1159/000539449/909222/Predictors-of-Poststroke-Cognitive-Decline-among>

In your revision ensure you cite all your sources (including your own works), and quote or rephrase any duplicated text outside the methods section. Further consideration is dependent on these concerns being addressed.

In your revision ensure you cite all your sources (including your own works), and quote or rephrase any duplicated text outside the methods section.

**Authors’ response:** Accepting your constructive comment, we have paraphrased the overlapping texts throughout the manuscript.

**Editor Comment #03:** Please upload a new copy of Figures 1 and 3 to 7 as the detail is not clear. Please follow the link for more information: <https://blogs.plos.org/plos/2019/06/looking-good-tips-for-creating-your-plos-figures-graphics/> <https://blogs.plos.org/plos/2019/06/looking-good-tips-for-creating-your-plos-figures-graphics/>

**Authors’ response:** Thank you! We have uploaded the figures with the required style.

**Editor Comment #04:** As required by our policy on Data Availability, please ensure your manuscript or supplementary information includes the following:

A numbered table of all studies identified in the literature search, including those that were excluded from the analyses.

For every excluded study, the table should list the reason(s) for exclusion.

If any of the included studies are unpublished, include a link (URL) to the primary source or detailed information about how the content can be accessed.

A table of all data extracted from the primary research sources for the systematic review and/or meta-analysis. The table must include the following information for each study:

Name of data extractors and date of data extraction

Confirmation that the study was eligible to be included in the review

All data extracted from each study for the reported systematic review and/or meta-analysis that would be needed to replicate your analyses.

If data or supporting information were obtained from another source (e.g. correspondence with the author of the original research article), please provide the source of data and dates on which the data/information were obtained by your research group.

If applicable for your analysis, a table showing the completed risks of bias and quality/certainty assessments for each study or outcome.  Please ensure this is provided for each domain or parameter assessed. For example, if you used the Cochrane risk-of-bias tool for randomized trials, provide answers to each of the signaling questions for each study. If you used GRADE to assess certainty of evidence, provide judgments about each of the quality of evidence factor. This should be provided for each outcome.

An explanation of how missing data were handled.

 This information can be included in the main text, supplementary information, or relevant data repository. Please note that providing these underlying data is a requirement for publication in this journal, and if these data are not provided your manuscript might be rejected.

**Authors’ response:** Recognizing your constructive feedback, we have uploaded the supplemental tables with their relevant information.

**Editor Comment #05:** It would be preferable to change posttraumatic to post-traumatic throughout the manuscript, including title.

**Authors’ response:** Thank you for your valuable feedback. We have changed posttraumatic to post-traumatic throughout the manuscript, including the title.

**Editor Comment #06:** In the abstract add the abbreviations for low and middle-income countries (LMICs) and post-traumatic stress disorder (PTSD) (line 34). Please use the abbreviated form in later sections of the abstract.

**Authors’ response:** Recognizing your feedback, we have revised the sessions.

**Editor Comment #07:** In the introduction, add the abbreviations for low and middle-income countries (LMICs) (line 71). Please use the abbreviated form in other parts of the introduction and other sections of the manuscript.

**Authors’ response:** Recognizing your feedback, we have revised the sessions.

**Editor Comment #08:** Major point: The prevalence of PTSD may vary at different time aftermath of a trauma (e.g., 1 month, 3 months, 6 months, and 12 months). How do the authors address this issue? How late was PTSD assessed after RTA in the original studies? It would be preferable to address this issue in subgroup analysis.

**Authors’ response:** Sure! The likelihood of developing PTSD is primarily explained within the short duration/the first months of the event/trauma. However, the majority of the included studies missed to report the prevalence of PTSD at 1^st^, 2^nd^, 3rd, 6^th^ and 12^th^ months of the event. These studies have reported only the lifelong PTSD following RTA.

**Editor Comment #09:** In Table 1, did all studies used PCL-civilian version for PTSD diagnosis?

**Authors’ response:** Thank you for your interesting question. The studies didn’t use only PCL-civilian version for PTSD diagnosis. I have revised the table by including all the diagnostic tools (CIDI, Composite International Diagnostic Interview; CPSSI, Child PTSD Symptom Scale Interview; IES-R, Revised Impact of Event Scale; MINI, Mini-International Neuro-psychiatric Interview; TSQ, Trauma Screening Questionnaire).

**Editor Comment #10:** The discussion needs a bit more work. Recommend for each key observations. It is better to compare the findings of this systematic review with other systematic reviews. The authors have made comparisons mostly with cross-sectional studies. I also encourage you to copyedit your article by a native English speaker (particularly the discussion section).

**Authors’ response:** Thank you for your constructive feedback! We have revised the manuscript, especially the discussion session by a language expert, and we have also compared this finding with previous findings of systematic review and meta-analysis.

**Reviewer #1:**

**Reviewer #1 comment and suggestion #01:** Abstract: Line 32: "Although several primary studies..." You mentioned "paucity of data" in line 36, which refers to scarcity. These statements appear contradictory. Could you consider rephrasing or clarifying your objective to resolve this?

**Authors’ response**: Thank you for your critical view of our manuscript! We initially perceived the "paucity of data" to mean multiple data, however, upon your valuable feedback, we have revised this session accordingly.

**Reviewer #1 comment and suggestion #02:** Introduction: "RTAs have become a global public health issue...” The data cited is from seven years ago. If possible, please provide more recent statistics to update the reference to the 2017 WHO report.

**Authors’ response:** Thank you for your critical view! We have used a recent report relatively.

**Reviewer #1 comment and suggestion #03:** Line 72: "More than 90% of road traffic deaths occur in low and middle-income countries..." Could you expand on the disparity between Sub-Saharan Africa and other regions?

**Authors’ response:** Thank you for your constructive feedback! We have mentioned the specific magnitude of PTSD among RTA survivors in SSA. .

**Reviewer #1 comment and suggestion #04:** Line 76: "In particular, PTSD is now a significant public health concern..." However, you cited references from 2006, 2015, and 2005, making it hard to support the "now" in your statement. Could you include more recent studies or reports to emphasize the current relevance of this concern, especially in the context of mental health trends in Sub-Saharan Africa?

**Authors’ response:** Thank you very much for your critical view! We have used recent references on their behalf.

**Reviewer #1 comment and suggestion #05:** While the introduction effectively outlines the issue, it could benefit from more focus on the factors driving the differences in PTSD rates globally.

**Authors’ response:** Thank you! In addition to narrating the magnitude and burden of PTSD among RTA survivors, we have also narrated the potential risk factors of PTSD among RTA survivors.

**Reviewer #1 comment and suggestion #06:** Line 119: "The studies were accessed through..." Could you explain why these databases were chosen? Were others, such as African Journals Online, considered?

**Authors’ response:** Thank you! We have chosen these search engines and databases because we could easily access all the relevant studies. We have also searched African Journals Online and Google to retrieve unpublished articles from their repositories.

**Reviewer #1 comment and suggestion #07:** Did you pretest the search strategy in the databases before conducting the actual search? If so, could you mention what adjustments were made?

**Authors’ response:** Yes! Initially we have used ʺPost-traumatic stress disorder AND ʺSub-Saharan Africaʺ MeSH terms to retrieve the relevant studies as a pretest. Finally, we have used ʺPost-traumatic stress disorder [MeSH term] AND (ʺPredictorsʺ [MeSH term] OR ʺAssociated factorsʺ [MeSH term] OR ʺRisk factorsʺ [MeSH term] OR ʺDeterminantsʺ [MeSH term]) AND ʺRoad traffic accident survivorsʺ [MeSH term] AND ʺSub-Saharan Africaʺ) to retrieve the relevant studies.

**Reviewer #1 comment and suggestion #08:** Line 132 (Eligibility criteria): Since the title is a systematic review and meta-analysis, could you clarify why qualitative studies were excluded?

**Authors’ response:** Thank you for your critical view! Even though we have mentioned it as exclusion criteria, unfortunately, we didn’t exclude a qualitative study from this study, and we have revised this session too.

**Reviewer #1 comment and suggestion #09:** Lines 141-146 (Data extraction): Could you specify what tools were used to assess inter-rater reliability between the independent reviewers?

**Authors’ response:** Thank you! Two independent reviewers (TMA and BMM) extracted the data using structured Microsoft Excel independently, and cross-checking was held each other. When discrepancies or missing between the extracted data were detected, the phase was repeated. When discrepancies between the data were continued again, the third reviewer (NE) was involved.

**Reviewer #1 comment and suggestion #10:** Lines 214-216 (Risk of bias assessment): You note that some studies raised concerns about the risk of bias but don’t elaborate on how this impacted the findings. A more detailed discussion here could strengthen this section.

**Authors’ response:** Thank you for your constructive feedback! We have performed the risk of bias assessment in detail, and attached as supplemental table.

**Reviewer #1 comment and suggestion #11:** Results: Search results: Why were 124 studies excluded after full-text review? Including a table with reasons for exclusion would improve transparency.

**Authors’ response:** Thank you! We have included a supplemental table stating the reasons why these studies have been excluded.

**Reviewer #1 comment and suggestion #12:** Discussion: Line 270: "The pooled prevalence of PTSD was 23.36%": You state that this is low compared to studies outside Africa but don’t explore why. Including a hypothesis or explanation would add depth to the discussion.

**Authors’ response:** Recognizing your valuable comment, we have elaborated it in the discussion session why this discrepancy occurred.

**Reviewer #1 comment and suggestion #13:** Lines 274-276: "The possible reasons for this difference..." Instead of listing possible reasons, could you elaborate on which explanation is most likely based on your data or literature?

**Authors’ response:** Thank you! We have elaborated the most likely reasons.

**Reviewer #1 comment and suggestion #14:** Line 287: "Similarly, the findings of this study..." Did you consider analyzing data by age group or by rural versus urban settings? These factors could potentially influence PTSD rates.

**Authors’ response:** Thank you! We have faced a difficulty to compare the data based on age group and rural versus urban because the majority of these studies didn’t have aggregate report PTSD in terms of these variables.

**Reviewer #1 comment and suggestion #15:** Line 318: "Special attention should be given..." Could you be more specific about the types of interventions or attention required for these populations?

**Authors’ response:** Recognizing your constructive comment, we have specified the specific intervention strategies.

**Reviewer #1 comment and suggestion #16:** Lines 319-320: "Future researchers shall..." This is a strong recommendation, but providing concrete examples of triangulated study designs would strengthen it.

**Authors’ response:** Thank you for your valuable feedback! We have also revised this session specifically.

**Reviewer #1 comment and suggestion #17:** General comments: Some sections are dense with technical terms. Simplifying the language in key parts could make the manuscript more accessible to a broader audience.
Many of the references are outdated. Please consider updating them with more recent studies, including the latest WHO data.

**Authors’ response:** Thank you! We have revised the whole manuscript by consulting language expert, and used updated references relatively.

**Reviewer #2:**

**Reviewer #2 comment and suggestion #01:** In Line 80 and Line 274, the authors cited and quoted a primary study from “Taiwan”. I would strongly recommend the author replace the term with “Chinese Taiwan”. I don’t mean to promote any political argument here, but please do consider how other Chinese scholars would cite this article if it were published. To avoid controversy in terms of storytelling and keep the reputation of journal, I would strongly recommend the authors follow the conduct of the UN.

**Authors’ response:** Recognizing your feedback, we have revised these sessions.

**Reviewer #2 comment and suggestion #02:** In the “Databases and search strategy” section, the authors first used the term PECO in line 105, yet changed it to PICO in line 115. Although they are essentially the same, I would recommend you unify the term for better storytelling.

**Authors’ response:** Thank you for your critical view! It was typo, and we have revised it.

**Reviewer #2 comment and suggestion #03:** The “Primary outcome measure of interest” and “Operational definition of variables” sections only defined the outcome of the prevalence of PTSD. I wonder whether it is possible to add another section explaining how you define the outcomes of potential risk factors. Apparently, the authors counted the number of cases in later analysis to quantify each risk factor. It would be better to state this point clearly.

**Authors’ response:** Thank you for your critical feedback again! We have revised this session too.

**Reviewer #2 comment and suggestion #04:** The main results of PTSD prevalence still show high heterogeneity after dividing by subgroups. Generally, such high heterogeneity indicates inconsistent results, as the authors suggested in the beginning. It might be okay if the author failed to resolve high heterogeneity, but please address this as a limitation.

**Authors’ response:** Sure! We were unable to demonstrate the potential source of heterogeneity even if we had performed subgroup analyses using the study area and period. Thus, we have mentioned it as a limitation.

**Reviewer #2 comment and suggestion #05:** Following comment 3, the reason I strongly recommend the authors define how they quantify risk factors clearly is that the measurement of the risk factors might vary. For depression, for example, some studies you identified used PHQ-9, and some used Self-Reporting Questionnaire 20. Their criteria for diagnosing depression vary. Furthermore, please can you confirm whether the depression measured in each study was depression post-road traffic accident or pre-existing depression? And whether this is consistent across all studies? Depression is a common comorbid of PTSD, such that only pre-existing depression could be regarded as a risk factor (not to mention whether they had potential baseline PTSD). For example, in Fekadu 2018, the study population was adult survivors already. So, depression was measured among survivors of post-road traffic accidents, which is a comorbid of PTSD, not a risk factor. As a result, I have strong reservations about the authors’ results on depression as a risk factor.
a. Therefore, please also elaborate on the study summary table (table 1) regarding this information (case number in terms of sex, depression, means of measurement of risk factors, and so on).
b. I would strongly recommend the authors at least carefully discuss these points in the discussion section and state limitations.

**Authors’ response:** Thank you for your valuable comment! The author used different measuring tools (Thank you for your interesting question. The studies didn’t use only PCL-civilian version for PTSD diagnosis. I have revised the table by including all the diagnostic tools (CIDI, Composite International Diagnostic Interview; CPSSI, Child PTSD Symptom Scale Interview; IES-R, Revised Impact of Event Scale; MINI, Mini-International Neuro-psychiatric Interview; TSQ, Trauma Screening Questionnaire) to quantify the primary and secondary outcomes. However, specifically to depression the majority of included studies were vague (unclear whether it is pre-existing or comorbid). According to their discussion session it seems to be pre-existing mental illnesses, including depression, and potentially it will be pre-existing depression to be a risk factor. We have also mentioned it as a limitation.

**Reviewer #2 comment and suggestion #06:** I Furthermore, the AOR estimated by the case numbers also suffered from the inflation caused by the multicollinearity of repeated counting of cases. That is, none of the studies distinguished whether the case had only one risk factor or several risk factors. For example, a female patient who had depression and substance use issues would be counted as a case for each factor. However, it is unclear which one was the key issue or whether there was interaction from other factors. This does not mean the results became completely unreliable, but please at least discuss this issue in the discussion section.

**Authors’ response:** Thank you for your constructive comment! It was unclear whether there was an inflation of AORs caused by the multicollinearity of repeated counting of cases in the included studies, and we have mentioned it in the discussion session.

**Reviewer #2 comment and suggestion #07:** I have reservations about the latter 3 factors (duration since the accident, social support and substance use). Generally, it requires at least four datasets to perform data synchronization in meta-analysis. None of these factors excessed 4. I would consider deleting them by saying there were not enough valid data or at least addressing the limitation.

**Authors’ response:** Thank you for your constructive comment! We have mentioned it as a limitation in the manuscript stating as “the majority of the associated factors (duration since the accident, social support, and substance use) lacked the minimum datasets needed for data synchronization in the meta-analysis”.

**Reviewer #2 comment and suggestion #08:** Considering the above, the conclusion and abstract should also be amended accordingly.

**Authors’ response:** Thank you for your constructive feedback! We have amended/revised both the abstract and conclusion sessions of the manuscript accordingly based on the given feedbacks.
